# Genetic diversity analysis of proso millet (*Panicum miliaceum* L.) germplasm resources based on phenotypic traits and SSR markers

**DOI:** 10.3389/fpls.2025.1649200

**Published:** 2025-09-08

**Authors:** Ziyang Lv, Yixuan Yang, Hanghang Hou, Shangkun Yang, Zhijia Cui, Xi Zhang, Jing Li, Yuhao Yuan, Minxuan Liu, Baili Feng

**Affiliations:** ^1^ State Key Laboratory of Crop Stress Biology in Arid Areas, College of Agronomy, Northwest A&F University, Yangling, Shaanxi, China; ^2^ School of Life Sciences, Shaanxi Key Laboratory of Research and Utilization of Resource Plants on the Loess Plateau, Yan’an University, Yan’an, Shaanxi, China; ^3^ College of Agronomy, Henan Agricultural University, Zhengzhou, Henan, China; ^4^ Institute of Crop Sciences, Chinese Academy of Agricultural Sciences, Beijing, China

**Keywords:** proso millet, germplasm resources, SSR marker, genetic diversity, genetic structure

## Abstract

**Introduction:**

Germplasm resources are vital for food security and agricultural sustainability, providing the basis for seed industry innovation.

**Methods:**

In the present study, 1,582 proso millet (*Panicum miliaceum* L.) germplasm resources, comprising landraces, cultivars, wild varieties, and foreign varieties, were sown in early June 2024 at the Yulin Minor Grain Comprehensive Experimental Demonstration Station. Subsequently, the genetic diversity was analyzed according to 14 agronomic, 5 yield, and 3 grain traits.

**Results:**

Significant phenotypic diversity was observed: agronomic trait diversity indices ranged from 0.15 to 2.10, with straw weight per plant showing the highest variation coefficients (60.03%). Yield traits exhibited diversity indices of 1.95-2.08 and variation coefficients of 14.94-37.37%. Grain traits had diversity indices exceeding 2, with the lowest variation coefficients (5.22-6.61%). Principal component analysis identified 4 key components, with tiller number and panicle length having the highest loading, leading to the selection of 10 superior germplasms. Cluster analysis grouped 1,582 samples into 5 categories, from which 147 representative germplasms were chosen. Then, 80 SSR primers were designed, 15 of which revealed polymorphism, confirming high genetic variation among these 147 germplasms. Varieties from Northwest China and Loess Plateau region showed the greater diversity.

**Conclusion:**

These findings provide a scientific foundation for the efficient utilization and breeding of proso millet germplasm resources.

## Highlights

- The diversity of 1,582 global proso millet germplasm resources was evaluated.
- Phenotypic and molecular data were combined to assess genetic diversity.
- SSR markers were developed to provide a foundation for genetic breeding.
- Fingerprint maps were constructed to facilitate digital management.

## Introduction

Proso millet (*Panicum miliaceum* L.) is one of the oldest domesticated crops worldwide, with its history of cultivation dating back to 10,000 years ago in China (Wang et al., 2016a, b; Shi et al., 2019; Jiang et al., 2024). Owing to its long cultivation history, extensive geographical spread, and prolonged domestication, proso millet germplasm resources are particularly rich and diverse (Wang et al., 2016a). Globally, approximately 29,000 germplasm resources have been conserved, and high variability exists among them (Vetriventhan et al., 2019). In China, a total of 9,885 germplasm resources have been documented, with 8,515 collected over a 22-year span from 1982 to 2003 (Wang et al., 2005). Of these 8,515 resources, 98% originate from 23 provinces of China, while 2% are from 14 other countries (Wang et al., 2016a). Its short growth period, drought resistance, and tolerance to poor soils makes it a pioneer crop suitable for disaster relief, famine preparation, and yield enhancement (Zhao, 2011), and it thus plays a pivotal role in enhancing food security and fostering agricultural diversification (Rajput and Santra, 2016; Zhang et al., 2019; Yang et al., 2023; Khound et al., 2024). Recent studies have advanced from core germplasm construction (Upadhyaya et al., 2011) and phenotypic-genetic analysis (Rajput and Santra, 2016) to integrated multi-trait evaluation (Vetriventhan and Upadhyaya, 2018, 2019), successfully identifying elite germplasm groups with high-yield potential, large-grain characteristics, and multi-nutrient enrichment. However, these breakthroughs remain constrained by substantial geographical bias, fragmented trait analysis, and homogenized germplasm coverage, critically impeding the in-depth exploration of genome-wide adaptive mechanisms of proso millet.

Simple sequence repeat (SSR) markers can be used to directly detect DNA molecular structure variation (Weber, 1990), reflecting differences with high sensitivity, stability, and ease of use. It is thus commonly used in genetic diversity analysis (Badiane et al., 2012; Wang et al., 2012; Rajput et al., 2014; Krishna et al., 2018; He et al., 2024), germplasm identification, and DNA fingerprinting. This technology is minimally impacted by environmental factors, ensuring stable and reliable results (Grover and Sharma, 2016), and it has been widely applied to crops, including foxtail millet (Li et al., 1998; Wang et al., 2012), maize (Lilia et al., 2008), soybean (Wen et al., 2009), sorghum (Li et al., 2010), and cowpea (Badiane et al., 2012). Rajput et al. (2014) analyzed 548 switchgrass SSR markers and identified 339 that could be applied to proso millet, with 254 exhibiting polymorphism. Hunt et al. (2011, 2018) conducted a genomic analysis of proso millet accessions collected from across the Eurasian continent, determining that genetic clustering aligned with their geographic origins and confirming the western Loess Plateau as the primary domestication center for proso millet. Liu et al. (2016) used 67 SSR markers to analyze 88 proso millet varieties, detecting 179 alleles and revealing rich genetic variation within ecological zones, along with complex intergroup relationships. DNA molecular identifiers (IDs) convert genetic differences into numerical codes, thereby assigning a distinct digital identifier to each variety (Tian et al., 2015). This method has been widely applied to sweet potatoes (Meng et al., 2018), potatoes (Duan et al., 2019), and mustard (Panigrahi et al., 2018) since its initial development. However, there have been no published studies reporting DNA molecular IDs for proso millet.

The present study initially utilized phenotypic traits to evaluate the diversity of 1,582 germplasms, comprising 1,516 landraces, 32 cultivars, 9 wild varieties and 25 foreign varieties. The 1,577 germplasms from China were specifically sourced from the following provinces: Inner Mongolia, Heilongjiang, Jilin, Liaoning, Hebei, Shandong, Gansu, Qinghai, Xinjiang, Shanxi, Shaanxi, and Ningxia. Based on the clustering analysis of phenotypic traits, the 1,582 germplasms were subdivided into five groups that were then ranked in descending order based on comprehensive F values and compared against qualitative traits. This approach resulted in the identification of 147 representative superior germplasms. These germplasms demonstrated outstanding overall phenotypic traits while representing a substantial proportion of the genetic diversity among all germplasms. Subsequently, genetic diversity analysis was performed on these 147 germplasms using 15 highly polymorphic SSR markers alongside the construction of their molecular IDs. This work provides an empirical foundation for the protection and utilization of proso millet germplasm.

## Materials and methods

### Material source

**Figure 1:**
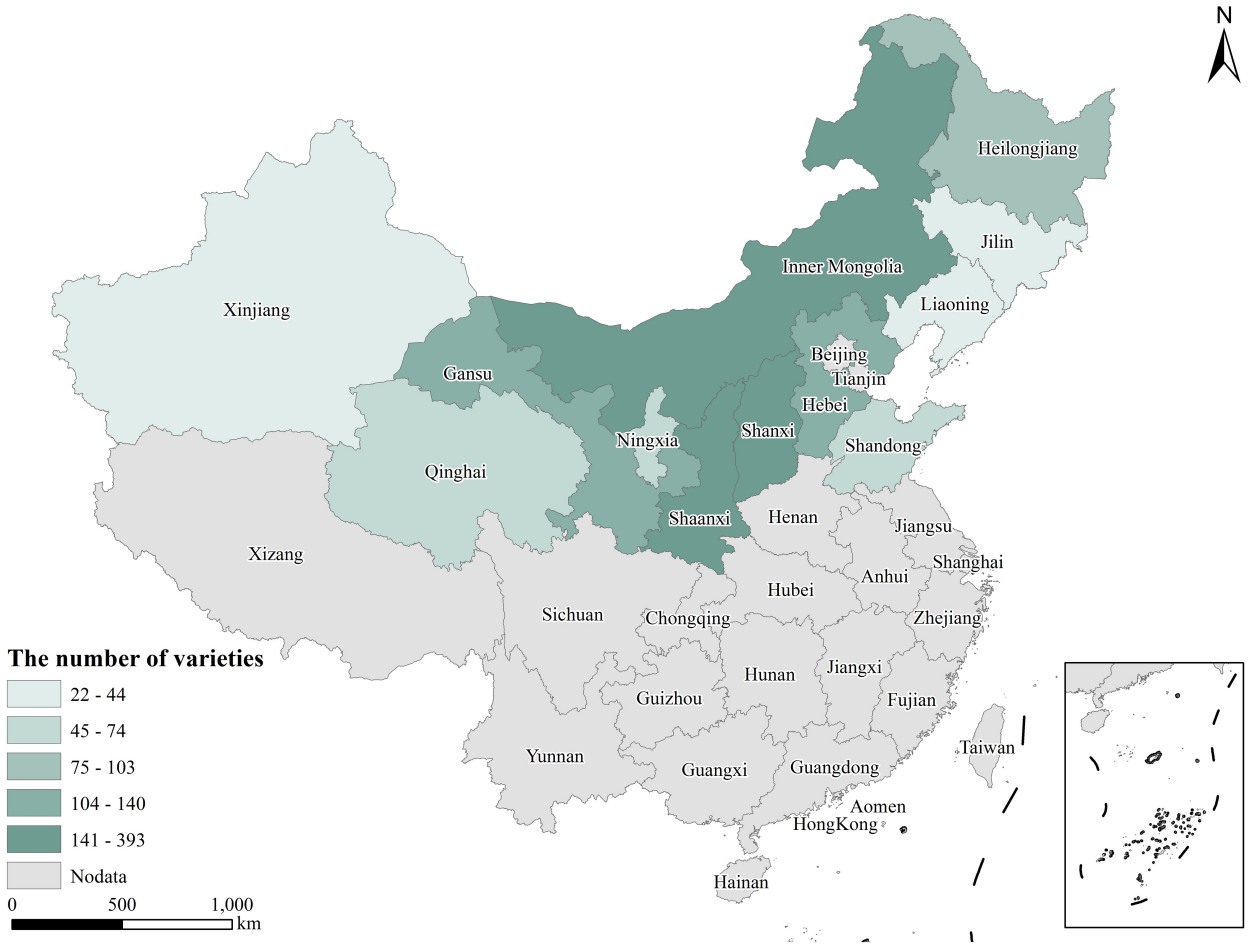
The sources of 1,557 proso millet germplasm resources.

A total of 1,582 proso millet germplasm resources (including 1,516 landraces, 32 cultivars, 9 wild varieties from China, and 25 foreign varieties, with the accessions from China specifically sourced from Inner Mongolia, Heilongjiang, Jilin, Liaoning, Hebei, Shandong, Gansu, Qinghai, Xinjiang, Shanxi, Shaanxi, and Ningxia), were obtained from the Crop Science Institute of the Chinese Academy of Agricultural Sciences (**Figure 1**, **Supplementary Tables S1**, **S2**).

### Field phenotypic trait determination

#### Field experiment design

The materials were sown in early June 2024 at the Yulin Minor Grain Comprehensive Experimental Demonstration Station, Shaanxi, China. Each variety was planted in three randomized plots (3 meters long × 2 meters wide) with 0.33-meter inter-plot spacing to ensure independence of samples. Soil fertility within the experimental field was uniform across all plots. Irrigation, fertilization, weeding, and other field management practices adhered to local large-scale farming protocols.

#### Phenotypic trait determination

During the growth period, inflorescence color, inflorescence density, axis shape, branched spike length, projection on branch base, angle of divergence of branches, panicle type, and trichome, among other measurements, were observed and recorded. At maturity, measurements of stem height, main stem nodes, stem diameter, panicle length, tiller number, branching habit, panicle weight per plant, grain weight per plant, straw weight per plant, and shatter resistance were recorded. After harvest, thousand-grain weight, grain length, grain width, and grain length-to-width ratio were measured.

Inflorescence color was visually evaluated in each test plot, using the principle of maximum similarity for classification. Inflorescence density was assessed by observing the spikelet distribution on the branches. The straightness or curvature of the axis was evaluated visually according to similar criteria. Branch spike length was measured by selecting three random plants from each plot; the length of the first branch on the main stem was measured with a ruler, and the average was used to determine the short, intermediate, or long type. Projection on branch base was examined for the presence of swollen joint-like structures and categorized according to presence, absence, or degree. The angle of divergence of branches was measured with a protractor on three randomly selected plants per plot, with the average determining the small, intermediate, or large type. Panicle type was qualitatively assessed based on morphology. Trichomes were observed to determine their length and density. Stem height was measured from the plant base to the panicle base on three randomly selected plants per plot, and these measurements were averaged. The number of main stem nodes above ground was visually counted for three plants per plot, and the average was then calculated. Stem diameter was measured using calipers at the maximum internode diameter of the basal main stem, with the average obtained from three plants. Panicle length was measured from the first branch node to the top of the plant and averaged across three plants per plot. Tiller number was counted for three randomly selected plants per plot and averaged. Branching habit was visually assessed for conditions between leaf axils on stem nodes. Panicle weight per plant was determined by cutting and weighing three panicles from the first branch and averaging their weights. Grain weight per plant was determined by threshing and weighing grain from three panicles, with the weights then averaged. Straw weight per plant was measured by removing three panicles, cutting off roots, and weighing the stems and leaves, with the weights then averaged. Shatter resistance was assessed by allowing the panicle to drop freely three times, after which the shattering percentage was calculated, which was the basis for determining the shattering resistance type. Thousand-grain weight was calculated by cleaning, drying, and weighing two samples of 500 seeds with an error margin of 0.1 g. Grain length and width were measured using a seed analyzer, with values averaged from three measurements.

The assignment criteria for panicle type, inflorescence density, branched spike length, and shatter resistance were based on the methodology described by Zhang et al. (2019) and are detailed in **Supplementary Table S3**.

### DNA extraction and detection

Leaf samples were collected at the three-leaf stage, and plant DNA was extracted from them using a modified CTAB method. DNA quality was assessed by 1% agarose gel electrophoresis, while DNA concentration was determined using a nucleic acid detector (NanoDropOne).

### SSR primer design

SSR locus detection for the transcriptome unigenes of salt-tolerant varieties was performed using MISA software. The minimum repeat thresholds were set to 10, 6, 5, 5, 5, and 5 for mono-, di-, tri-, tetra-, penta-, and hexa-nucleotide repeat sequences, respectively. The SSR sequences obtained were then utilized for primer design with Primer 3.0 software. Eighty SSR primers (**Supplementary Table S4**) were synthesized by Beijing TSINGKE Biological Co., Ltd.

### SSR primer population amplification and electrophoresis detection

Twenty varieties with significant phenotypic differences (**Supplementary Table S5**) were used for polymorphism screening. Fifteen primers (**Supplementary Table S6**) yielded clear amplification bands that revealed stable polymorphisms, making them suitable for subsequent genetic diversity analysis of 147 identified superior proso millet germplasm resources (**Supplementary Tables S7**, **S8**). PCR amplification was conducted using the EasyCycler PCR system, and PCR products were separated by 8% polyacrylamide gel electrophoresis, followed by silver staining for band detection.

### Statistical analysis

Microsoft Excel 2021 was employed to compile phenotypic data, while SPSS 27.0 and Origin 2025SR1 were employed to analyze and visualize phenotypic data. Electrophoresis results were manually scored to create a binary data matrix. Genetic diversity parameters were calculated using PopGene 1.32 and PowerMarker 3.25, and cluster analyses were performed using MEGA 6.06. Population genetic structure and principal component analyses were conducted with Structure 2.34 and NTSYSpc 2.11. Based on SSR marker scoring results, data were sequentially encoded to construct DNA molecular IDs. QR code online technology (https://cli.im/text) facilitated the conversion of basic information into QR code DNA molecular IDs.

## Results

### Analysis of agronomic trait diversity in proso millet

**Figure 2:**
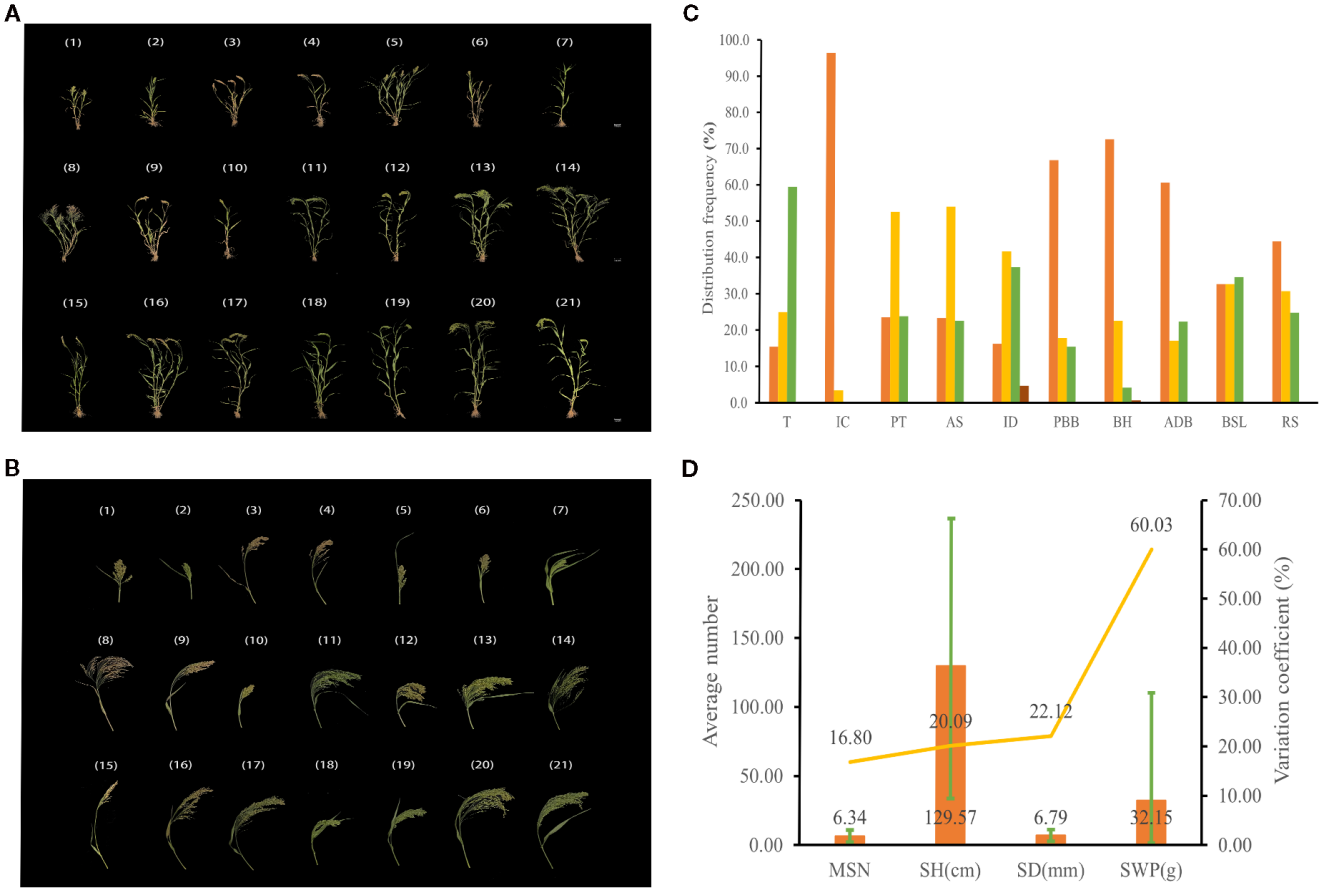
Agronomic trait diversity of proso millet. **(a)** Entire plant; **(B)** Panicle trait; **(C, D)** Agronomic trait. From 1 to 21 are Heidaetou, Shihuqianjingmi, Hongmi, Shuanglimizi, Linhedahuangmizi, Heitounianmizi, Zhouquhuangmizi, Helinxiaoqingmi, Dahuangmizi, Qianjingshu, Mizi, Hongmizi, Baimizi, Hongmizi, Gumi13, Bamenghongmizi, Linxidahongshu, Huangruanshu, Heimizi, Gancaohuangmizi, and Dalishu. These accessions display significant differences in agronomic traits and can effectively represent the diversity of germplasm resources. T, trichome; IC, inflorescence color; PT, panicle type; AS, axis shape; ID, inflorescence density; PBB, projection on branch base; BH, branching habit; ADB, angle of divergence of branches; BSL, branched spike length; RS, shatter resistance. Orange and yellow of inflorescence color denote green and purple; Orange, yellow, and green of panicle type correspond to scattered, lateral, and compact types, the sequence from orange to yellow to green (to brown) of remaining traits signifies a systematic enhancement in rank. MSN, main stem nodes; SH, stem height; SD, stem diameter; SWP, straw weight per plant. The bar chart illustrates the average values, the line graph depicts the coefficients of variation, and the error bars indicate the range.

The genetic diversity indices of 14 agronomic traits, 5 yield traits, and 3 grain traits were evaluated (**Figure 2**, **Supplementary Table S9**). The genetic diversity index was lowest for inflorescence color (0.15) and highest for stem diameter (2.10) among the 14 agronomic traits (comprising 10 qualitative and 4 quantitative traits). Notably, there were two traits with genetic diversity indices exceeding 2, both quantitative in nature. The differences in the frequency distribution of various qualitative traits are presented in **Figure 2C**. For the 1,582 germplasms, inflorescence color was predominantly green (96.5%), with purple accounting for merely 3.5%. The dense trichome type (59.5%) was the main trichome type. Regarding panicle type, the lateral type was predominant (52.6%), whereas scattered and compact types represented 23.6% and 23.8% of accessions, respectively. The main axis shape was primarily slightly bend (54.1%), with erect and bend types accounting for 23.3% and 22.6% of accessions, respectively. Inflorescence density was chiefly intermediate (41.7%) and slightly dense (37.4%). Most varieties lacked projections on branch bases (66.8%) and branching habits (72.6%), with minor angle deviations present in most varieties (60.6%). Short, intermediate, and long-branched spike length were evenly distributed, accounting for 32.7%, 32.7%, and 34.6% of accessions, respectively. Furthermore, the majority of varieties exhibited weak shatter resistance (44.4%), while strong shatter-resistant varieties were the least frequent (24.8%). As shown in **Figure 2D**, the average number of main stem nodes among the 1,582 germplasms was 6.34, with a variation coefficient of 16.8%. Stem height ranged from 33.67 to 236.67 cm, with an average of 129.57 cm, reflecting nearly a 7-fold disparity between the extrema, accompanied by a variation coefficient of 20.09%. The average stem diameter was 6.79 mm, with a variation coefficient of 22.12%. Straw weight per plant spanned from 1.53 to 110.10 g, averaging 32.15 g, with a 72-fold disparity between extrema, marked by the largest variation coefficient (60.03%).

### Analysis of yield trait diversity in proso millet

**Figure 3:**
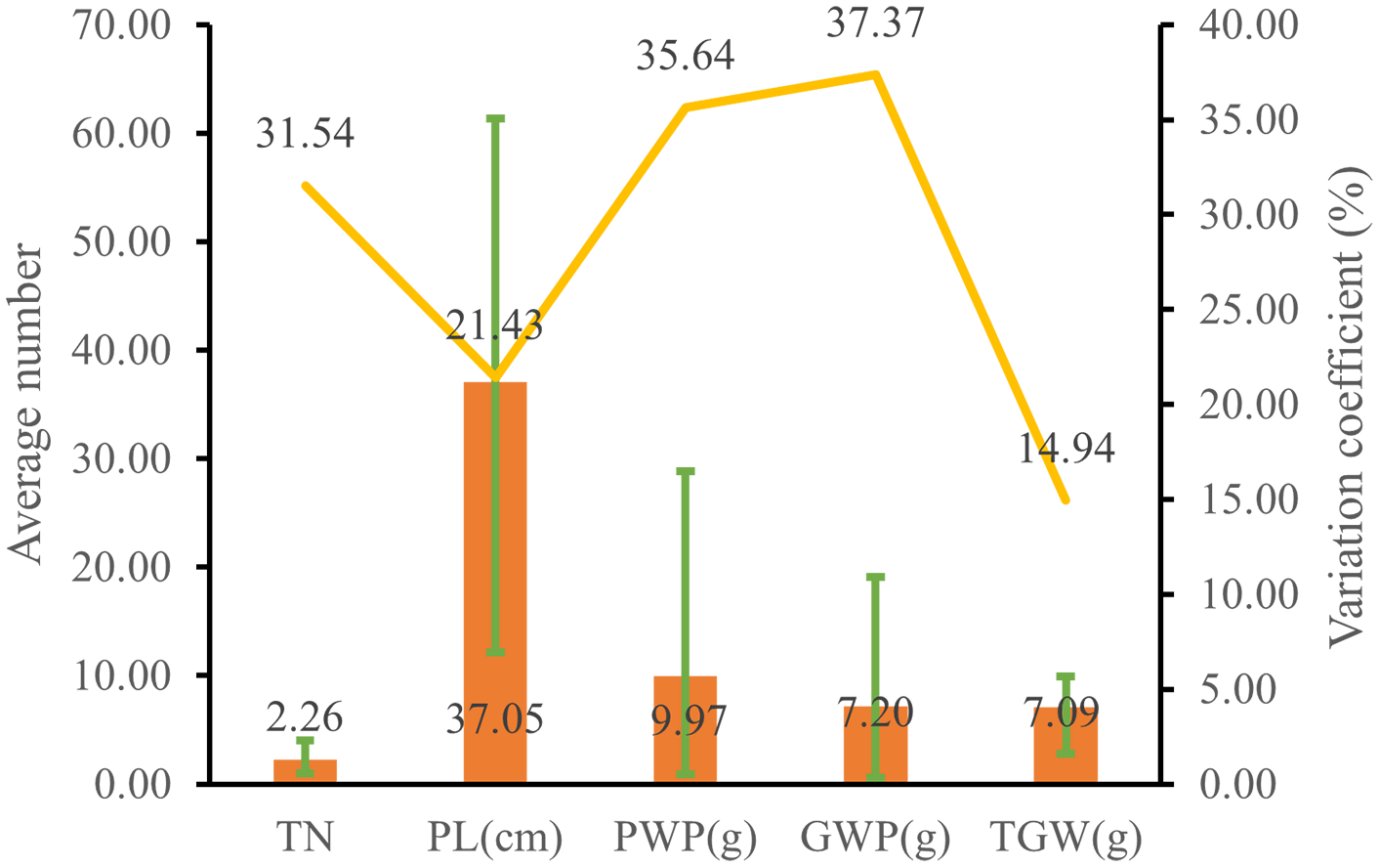
Yield trait diversity of proso millet. TN, tiller number; PL, panicle length; PWP, panicle weight per plant; GWP, grain weight per plant; TGW, thousand-grain weight. The bar chart illustrates the average values, the line graph depicts the coefficients of variation, and the error bars indicate the range.

The genetic diversity indices of yield traits ranged from 1.95 to 2.08, with tiller number having the lowest index at 1.95, while grain weight per plant had the highest at 2.08 (**Supplementary Table S9**). The genetic diversity indices for panicle length, panicle weight per plant, and thousand-grain weight all exceeded 2. As shown in **Figure 3**, the average tiller number among 1,582 germplasms was 2.26, with a considerable variation coefficient (31.54%). The average panicle length was 37.05 cm, exhibiting a variation coefficient of 21.43%. Panicle weight per plant ranged between 0.88 and 28.83 g, averaging 9.97 g, with a 33-fold difference between extrema and a substantial variation coefficient (35.64%). Grain weight per plant varied from 0.61 to 19.11 g, averaging 7.20 g, displaying a 32-fold difference between extrema and a high variation coefficient (37.37%). The average thousand-grain weight was 7.09 g, with a variation coefficient of 14.94%. The above results collectively indicate there was substantial variation in yield traits among proso millet accessions, with considerable genetic diversity.

### Analysis of grain trait diversity in proso millet

**Figure 4:**
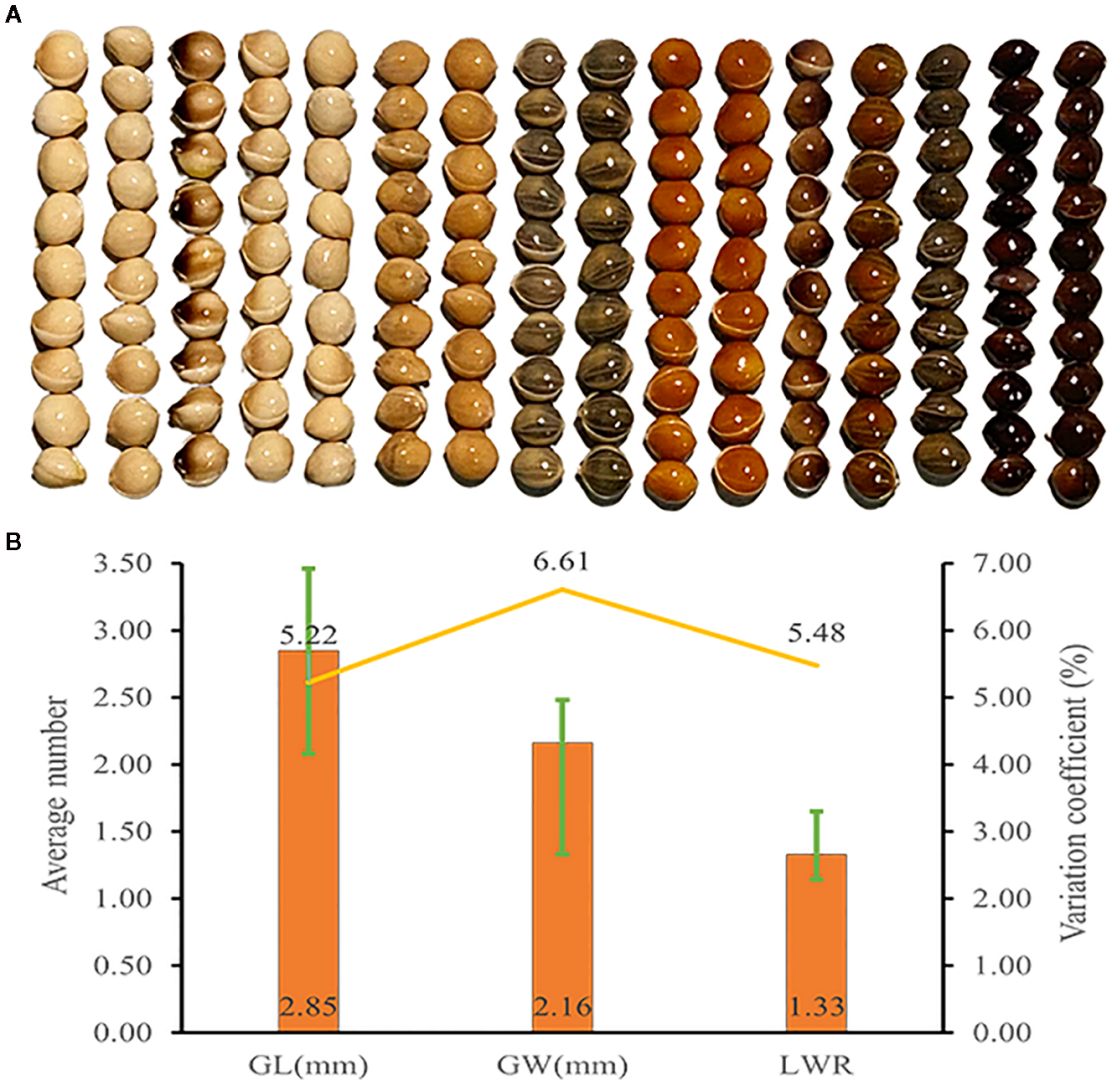
Grain trait diversity of proso millet. **(A)** Representative grain phenotype; **(B)** Grain trait. From left to right are Bairuanmi, Ziganbai, Hualianmi, Jiaojuruanmi, Zaershu, Gumi13, Huangmi, Jingyuanxiaoqing, Fengshouma, Mizi, Hongshuzi, Xiaohuashu, Qingshuihexiangsemizi, Sezhaertuo, Heitounianmizi, Heimizi. These accessions display significant differences in grain traits and can effectively represent the diversity of germplasm resources. GL, grain length; GW, grain width; LWR, grain length to width ratio. The bar chart illustrates the average values, the line graph depicts the coefficients of variation, and the error bars indicate the range.

The genetic diversity indices for grain length, grain width, and length-to-width ratio were 2.04, 2.02, and 2.00, respectively, indicating considerable genetic diversity in these grain traits (**Figure 4**, **Supplementary Table S9**). As shown in **Figure 4B**, the average grain length and width among 1,582 accessions were 2.85 mm and 2.16 mm, respectively. Grain length exhibited the lowest variation coefficient at 5.22%, whereas grain width had a variation coefficient of 6.61%. The average grain length-to-width ratio was 1.33, with a variation coefficient of 5.48%. Genetic diversity indices for grain traits were higher than those of certain agronomic and yield traits, while their variation coefficients are comparatively lower than those of all agronomic and yield traits.

### Correlation analysis among traits of proso millet

**Figure 5:**
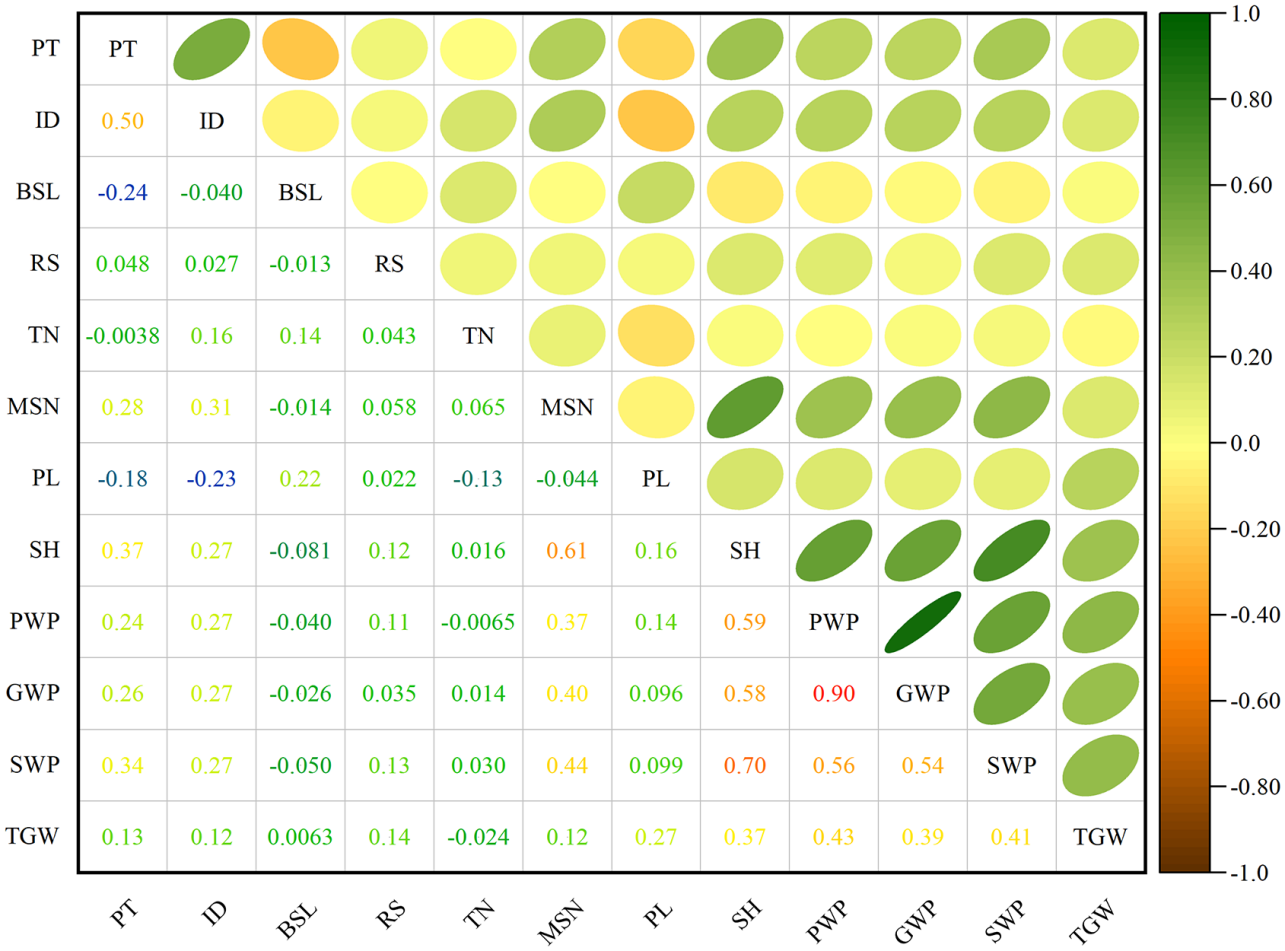
Correlation coefficients of various traits. PT, panicle type; ID, inflorescence density; BSL, branched spike length; RS, shatter resistance; TN, tiller number; MSN, main stem nodes; PL, panicle length; SH, stem height; PWP, panicle weight per plant; GWP, grain weight per plant; SWP, straw weight per plant; TGW, thousand-grain weight.

**Figure 5** presents the correlation analysis between four primary qualitative traits and eight representative quantitative traits. Among the qualitative traits, a highly significant positive correlation was observed between panicle type and inflorescence density (*r* = 0.50), while a highly significant negative correlation was observed between panicle type and branched spike length (*r* = -0.24). No correlations were detected among other qualitative traits. In examining the quantitative traits, the tiller number demonstrated a positive correlation with the main stem nodes (*r* = 0.07) and a negative correlation with panicle length (*r* = -0.13), yet showed no significant correlation with other traits. The number of main stem nodes was not correlated with panicle length but was highly significantly and positively associated with stem height (*r* = 0.61), panicle weight per plant (*r* = 0.37), grain weight per plant (*r* = 0.40), straw weight per plant (*r* = 0.44), and thousand-grain weight (*r* = 0.12). Panicle length was not correlated with the number of main stem nodes, was negatively correlated with the tiller number (*r* = -0.13), and was positively correlated with the stem height (*r* = 0.16), panicle weight per plant (*r* = 0.14), grain weight per plant (*r* = 0.10), straw weight per plant (*r* = 0.10), and thousand-grain weight (*r* = 0.27). Stem height, panicle weight per plant, grain weight per plant, straw weight per plant, and thousand-grain weight exhibited no correlations with tiller number. Stem height was highly significantly and positively associated with panicle weight per plant (*r* = 0.59), grain weight per plant (*r* =0.58), straw weight per plant (*r* = 0.70), and thousand-grain weight (*r* = 0.37). Panicle weight per plant was highly significantly and positively associated with grain weight per plant (*r* = 0.91), straw weight per plant (*r* = 0.56), and thousand-grain weight (*r* = 0.43). Grain weight per plant was highly significantly and positively associated with straw weight per plant (*r* = 0.54) and thousand-grain weight (*r* = 0.39). Straw weight per plant was highly significantly and positively associated with thousand-grain weight (*r* = 0.41). Among the relationships between qualitative and quantitative traits, panicle type was not correlated with the tiller number, but was negatively correlated with panicle length (*r* = -0.18) and positively correlated with the number of main stem nodes (*r* = 0.28), stem height (*r* = 0.37), panicle weight per plant (*r* = 0.24), grain weight per plant (*r* = 0.26), straw weight per plant (*r* = 0.34), and thousand-grain weight (*r* = 0.13). Inflorescence density was negatively correlated with panicle length (*r* = -0.23), and positively correlated with tiller number (*r* = 0.16), the number of main stem nodes (*r* = 0.31), stem height (*r* = 0.27), panicle weight per plant (*r* = 0.27), grain weight per plant (*r* = 0.27), straw weight per plant (*r* = 0.27), and thousand-grain weight (*r* = 0.13). Branched spike length was highly significantly positively correlated with the tiller number (*r* = 0.16) and panicle length (*r* = 0.22) and highly significantly negatively correlated with stem height (*r* = -0.08), while also exhibiting a significant negative correlation with straw weight per plant (*r* = -0.05); however, it showed no significant correlations with the remaining traits. Shatter resistance exhibited no correlation the tiller number, panicle length, or grain weight per plant, yet was highly significantly positively correlated with the number of main stem nodes (*r* = 0.06), stem height (*r* = 0.12), panicle weight per plant (*r* = 0.11), straw weight per plant (*r* = 0.13), and thousand-grain weight (*r* = 0.14).

### Principal component analysis and cluster analysis

Principal component analysis was employed to standardize the relative values of various traits, effectively eliminating factors with minimal impact but significant interference, to facilitate a comprehensive evaluation of proso millet germplasms (**Supplementary Table S10**). The first principal component accounted for 41.25% of the variance, the second for 13.59%, the third for 9.66%, and the fourth for 9.27%, with these four components collectively explaining 73.78% of the variance. The eigenvalues of these four principal components each exceeded 1, confirming their suitability for a comprehensive evaluation of proso millet germplasm. The highest loading values for the first principal component were associated with stem height, panicle weight per plant, and grain weight per plant. The second principal component was most positively correlated with thousand-grain weight, indicating that this trait heavily contributed to the component. The third principal component displayed the highest positive loadings for grain length and grain length-to-width ratio, suggesting it mainly reflects the overall condition of the grain. The fourth principal component exhibited higher absolute values for the tiller number and panicle length, showing its dependency on these traits.

Based on the score coefficient matrix of the four principal components, the comprehensive score (F) for each variety was calculated to rank proso millet germplasms by excellence (**Supplementary Table S11**). A higher F value indicates a better overall phenotype for that variety. The top 10 performing varieties were Hongruanmi, Hongruanmi, Laohuangmi, Getami, Ruantiaozhoumi, Zhuyeqing, Limashu, Mizi, Nuomizi, and Hongnianmi. Among these, Ruantiaozhoumi and Limashu are from Shanxi province; the remaining eight high-performing varieties are all from Shaanxi province.

**Figure 6:**
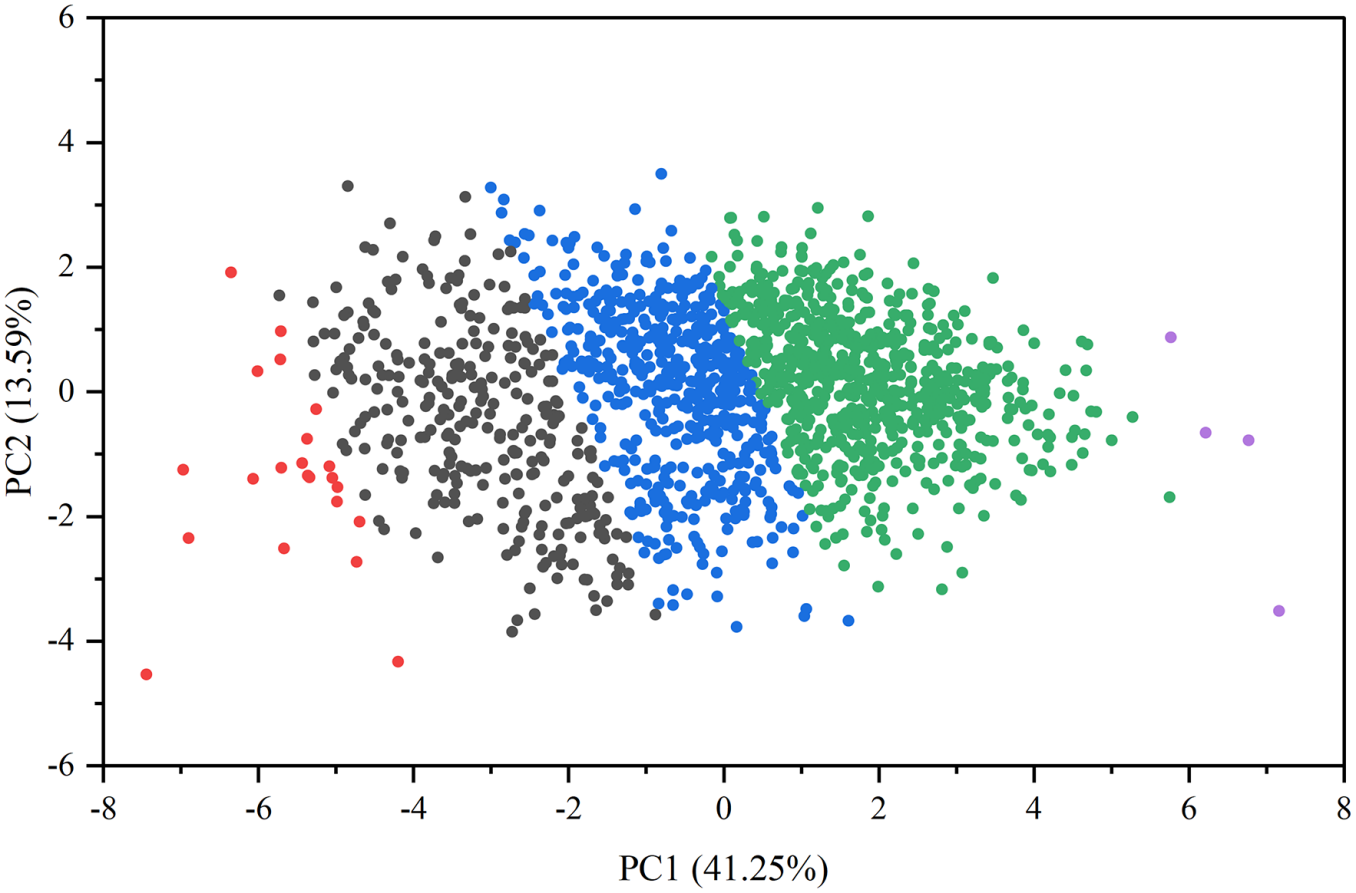
Principal component analysis of relative values of traits. The groups are color-coded as follows: red (Group 1), black (Group 2), blue (Group 3), green (Group 4), and purple (Group 5).

Cluster analysis of the 1,582 germplasms identified five distinct groups (**Figure 6**). The first group includes 22 varieties, including Etou and Sansui, mainly originating from Heilongjiang, accounting for 1.4% of accessions. The second group comprises 292 varieties, including Zaohongmi and Anchunwei, primarily sourced from Heilongjiang and Inner Mongolia, along with most foreign varieties, representing 18.5% of the germplasms. The third group consists of 528 varieties, including Daqihuangmizi and Taolibai, largely obtained from Shanxi and Inner Mongolia, comprising 33.4% of accessions. The fourth group contains 736 varieties, including Ruantiaozhuomi and Ziganmi (shu), predominantly sourced from Shanxi and Shaanxi, followed by Gansu and Inner Mongolia, constituting 46.4% of accessions. The fifth group consists of only four varieties, namely Hongruanmi, Hongruanmi, Getami, and Laohuangmi, all originating from Shaanxi, accounting for just 0.3% of accessions. Based on the clustering analysis of phenotypic traits, the germplasms within the five groups were ranked in descending order of comprehensive F values and compared against qualitative traits. This approach resulted in the identification of 147 representative superior germplasms, which were systematically selected for subsequent genetic diversity analysis.

### Genetic diversity analysis of proso millet

#### Polymorphic SSR primer selection

Twenty varieties with significant differences in phenotypic traits were subjected to polymorphism screening using 80 pairs of SSR primers. All primers successfully amplified bands, resulting in an effective amplification rate of 100%. Among these primers, 15 pairs demonstrated polymorphism, with a development efficiency of 18.75%. Ultimately, these 15 primer pairs were subsequently utilized for genetic diversity analysis of the 147 superior germplasms.

#### Genetic diversity analysis using SSR markers

Utilizing 15 pairs of polymorphic SSR primers, the genetic diversity of 147 proso millet germplasms was analyzed (**Table 1**). Across these 15 loci, a total of 30 observed alleles were identified, with each locus exhibiting two alleles, averaging 2.00. The effective allelic number range was between 1.03 (BLF21) and 1.96 (BLF79), with an average of 1.28. The Shannon diversity index ranged from 0.07 (BLF21) to 0.68 (BLF79), with an average of 0.33. Observed heterozygosity varied from 0 (BLF4, BLF47, BLF8, BLF9, BLF51, BLF52, BLF58, BLF59, BLF61, BLF27, BLF80) to 0.73 (BLF79), with an average of 0.06. Expected heterozygosity ranged from 0.03 (BLF21) to 0.49 (BLF79), averaging 0.19. Nei’s expected heterozygosity ranged from 0.03 (BLF21) to 0.49 (BLF79), averaging 0.19. The polymorphism information content spanned 0.17 (BLF51) to 0.59 (BLF79), averaging 0.39.

**Table 1:** Genetic parameters from the 15 polymorphic SSR markers used in the study.

Primer	*Na*	*Ne*	*I*	*Ho*	*He*	*Nei*	*PIC*
BLF-41	2	1.3478	0.4265	0.0145	0.2590	0.2580	0.3116
BLF-4	2	1.1912	0.2979	0.0000	0.1612	0.1605	0.3367
BLF-47	2	1.2800	0.3768	0.0000	0.2199	0.2188	0.4619
BLF-8	2	1.3081	0.3983	0.0000	0.2373	0.2355	0.4476
BLF-9	2	1.1133	0.2095	0.0000	0.1023	0.1017	0.4077
BLF-51	2	1.0606	0.1327	0.0000	0.0573	0.0571	0.1771
BLF-52	2	1.4764	0.5035	0.0000	0.3245	0.3227	0.5118
BLF-58	2	1.0533	0.1205	0.0000	0.0509	0.0506	0.3945
BLF-59	2	1.1492	0.2530	0.0000	0.1303	0.1298	0.2898
BLF-61	2	1.1995	0.3061	0.0000	0.1669	0.1663	0.2071
BLF-21	2	1.0267	0.0701	0.0263	0.0261	0.0260	0.3046
BLF-66	2	1.1730	0.2791	0.1603	0.1480	0.1475	0.2882
BLF-27	2	1.7676	0.6259	0.0000	0.4366	0.4342	0.5613
BLF-79	2	1.9625	0.6836	0.7340	0.4931	0.4904	0.5874
BLF-80	2	1.1150	0.2117	0.0000	0.1041	0.1031	0.3843
Mean	2	1.2816	0.3263	0.0623	0.1945	0.1935	0.3781
St.Dev	0	0.2685	0.1788	0.1903	0.1373	0.1366	

*Na*, number of alleles; *Ne*, number of effective alleles; *I*, Shannon’s diversity index; *Ho*, observed heterozygosity; *He*, expected heterozygosity; *Nei*, Nei’s expected heterozygosity; *PIC*, polymorphism information content.

#### Genetic diversity and similarity analysis from different regions

The analysis of genetic diversity parameters of proso millet germplasms across different regions (**Table 2**) revealed observed allelic variability from 1.33 to 1.87, highest in the Northwest China region and lowest in the Inner Mongolia region. Effective allelic variability ranged from 1.20 to 1.32, and was highest in the Loess Plateau region and lowest in the Northeast region. The highest observed heterozygosity was recorded in the Northeast region (0.08), and the lowest value was observed among germplasms from abroad (0.01). The highest expected heterozygosity was in the Loess Plateau region (0.20), and the lowest was in the Inner Mongolia region (0.13). Nei’s expected heterozygosity was highest in the Loess Plateau region (0.19) and lowest in the Inner Mongolia region (0.12). In terms of Shannon diversity index and polymorphism information content, varieties from Northwest China and Loess Plateau region exhibited higher values, while foreign varieties showed the lowest value, indicating greater genetic diversity in the Northwest and Loess Plateau region.

**Table 2:** Parameters of genetic diversity in six populations.

Population	*Na*	*Ne*	*I*	*Ho*	*He*	*Nei*	*PIC*
IMR	1.3333±0.4880	1.2138±0.3517	0.1849±0.2801	0.0667±0.2582	0.1325±0.2050	0.1245±0.1930	0.2877
NER	1.4000±0.5071	1.1961±0.2944	0.1948±0.2578	0.0794±0.2317	0.1365±0.1877	0.1253±0.1715	0.3652
NR	1.6667±0.4880	1.2888±0.3386	0.2850±0.2570	0.0672±0.1742	0.1859±0.1857	0.1808±0.1800	0.3471
NWR	1.8667±0.3519	1.2727±0.2502	0.3131±0.2014	0.0539±0.1687	0.1915±0.1441	0.1887±0.1420	0.3371
LPR	1.7333±0.4577	1.3177±0.3627	0.3025±0.2621	0.0713±0.2271	0.1984±0.1942	0.1932±0.1890	0.3573
Abroad	1.3571±0.4972	1.2816±0.4323	0.2158±0.3129	0.0143±0.0535	0.1789±0.2677	0.1503±0.2226	0.2345

IMR, Inner Mongolia region; NER, Northeast region; NR, Northern region; NWR, Northwest region; LPR, Loess Plateau region. The same as below.

Analysis of genetic distance and genetic consistency parameters of proso millet germplasms from different regions (**Table 3**) showed that genetic distance ranged from 0.01 to 0.21, averaging 0.08, and genetic consistency ranged from 0.81 to 0.99, averaging 0.92. The genetic distance was greatest between Northwest region and foreign accessions (0.21), with the lowest genetic consistency (0.81), while accession from the Northeast and the Northwest showed the closest genetic distance (0.01) and highest genetic consistency (0.99). These findings indicate that greater geographical separation is associated with greater genetic distance, lower genetic consistency, and more distant kinship; conversely, closer geographical proximity is correlated with closer genetic distance, higher genetic consistency, and nearer kinship.

**Table 3:** Nei's genetic identity and genetic distance from different populations.

Population	IMR	NER	NR	NWR	LPR	Abroad
IMR	-	0.9776	0.9590	0.9865	0.9636	0.8204
NER	0.0227	-	0.9617	0.9901	0.9853	0.8138
NR	0.0418	0.0391	-	0.9781	0.9751	0.8218
NWR	0.0136	0.0099	0.0221	-	0.9866	0.8123
LPR	0.0371	0.0148	0.0252	0.0135	-	0.8529
Abroad	0.1980	0.2060	0.1963	0.2079	0.1591	-

Nei's genetic identity (above diagonal) and genetic distance (below diagonal).

### Cluster analysis based on UPGMA

**Figure 7:**
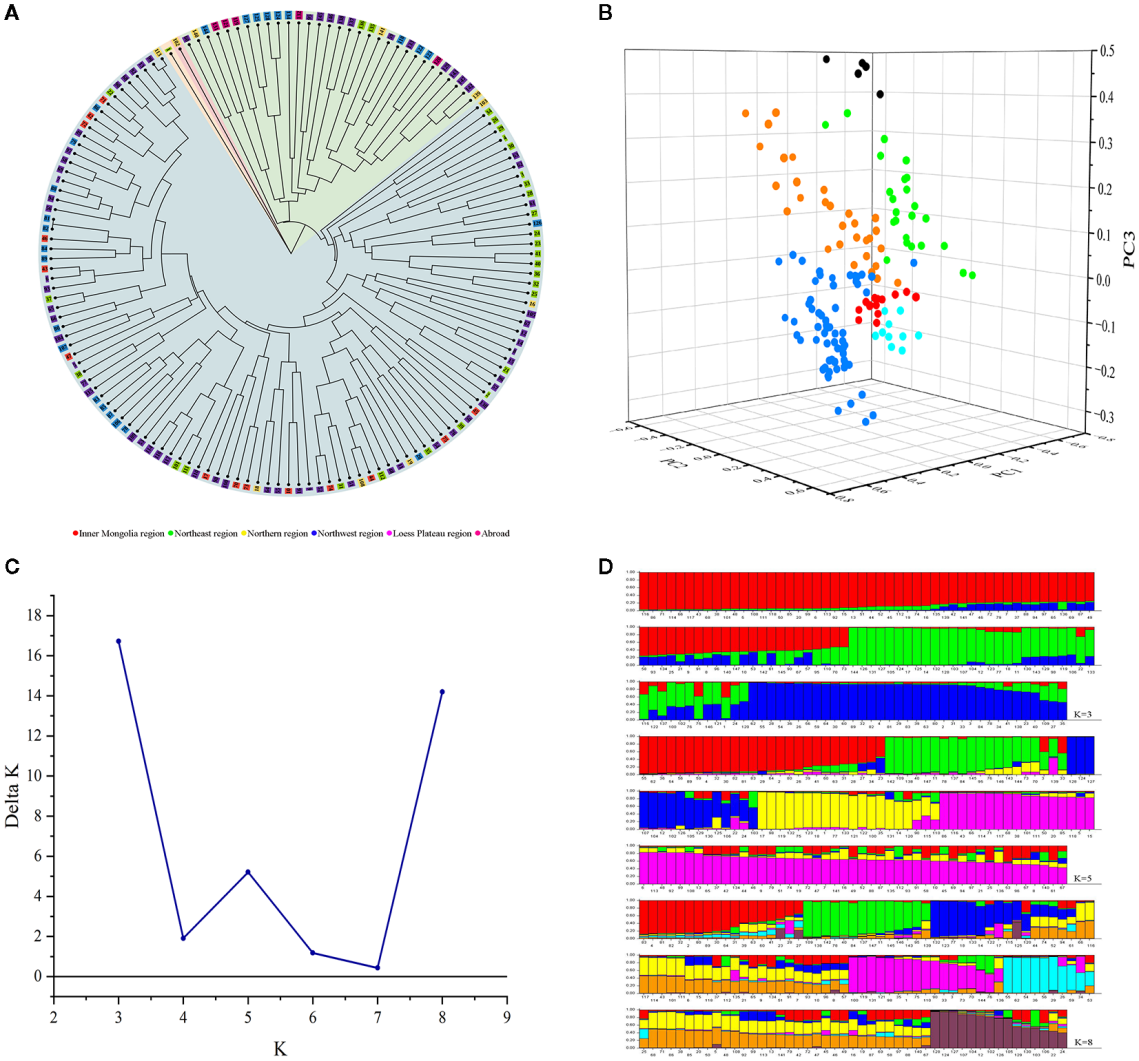
**(A)** Cluster analysis of 147 proso millet germplasms based on UPGMA. Numbers represent serial number of accessions. **(B)** Principal component analysis of proso millet germplasms. The groups are color-coded as follows: red (Inner Mongolia region), yellow (Northeast region), aqua (Northern region), green (Northwest region), blue (Loess Plateau region) and black (Abroad). **(C)** Population modeling of 147 proso millet germplasms based on structure. **(D)** Population genetic structure graph of 147 proso millet germplasms based on structure. Numbers in the horizontal axis represent serial number of accessions.

Cluster analysis of 147 proso millet germplasms was conducted by unweighted pair group method with arithmetic mean analysis (UPGMA), which indicated that the germplasms could be divided into four groups (**Figure 7A**, **Table 4**). Group 1 encompasses one variety, sourced from Heilongjiang, representative of the Northeast region. Group 2 includes one variety, sourced from Shandong, representative of the Northern region. Group 3 comprises 31 varieties with 8 from Shanxi, 6 from Qinghai, 3 from Gansu, 3 from Ningxia, 3 from Hebei, 2 from Heilongjiang, 1 from Shaanxi, and 5 from abroad, mainly from the Loess Plateau and Northwest regions. Group 4 contains 114 varieties, with contributions from Shanxi (22), Shaanxi (22), Heilongjiang (19), Inner Mongolia (14), Gansu (9), Jilin (7), Ningxia (7), Xinjiang (5), Hebei (4), Qinghai (2), Shandong (2), and Liaoning (1), primarily from the Loess Plateau, Northeast, and Northwest regions. Additionally, **Figure 7A** and **Table 4** collectively illustrate that all foreign varieties are consolidated within Group 3.

**Table 4:** Group distribution of 147 proso millet germplasms based on UPGMA.

Province	Group1	Group2	Group3	Group4
Inner Mongolia				14
Heilongjiang	1		2	19
Jilin				7
Liaoning				1
Hebei			3	4
Shandong		1		2
Gansu			3	9
Qinghai			6	2
Xinjiang				5
Shanxi			8	22
Shaanxi			1	22
Ningxia			3	7
Abroad			5	
Total	1	1	31	114

### Principal component analysis of genetic differences

Three-dimensional principal component analysis (PCA) was conducted on the 147 proso millet superior germplasms, as shown in **Figure 7B**. The first three principal components, denoted as PC1, PC2, and PC3, accounted for 21.37%, 18.87%, and 17.39% of the total variance, respectively, with a cumulative explanation of variance reaching 57.63%. The 147 germplasms were thus classified into six groups: 14 from the Inner Mongolia region, 30 from the Northeast region, 10 from the Northern region, 25 from the Northwest region, 63 from the Loess Plateau region, and 5 from abroad, with each group corresponding to their geographical sources.

### Population genetic structure analysis based on structure

The genetic structure of 147 proso millet accessions was analyzed using Structure. Peaks in the number of genetic populations (*K*) were identified at *K* = 3, *K* = 5, and *K* = 8 (**Figure 7C**), prompting analysis under both models. For *K* = 3, the germplasms were categorized into three groups as illustrated in **Figure 7D**. Group 1, marked in red in **Figure 7D** (73 varieties), predominantly sourced from the Loess Plateau, and Inner Mongolia regions. This includes varieties from Shanxi (19), Shaanxi (13), and Inner Mongolia (12). Group 2, marked in green in **Figure 7D** (39 varieties), primarily consists of varieties mainly originating from the Loess Plateau, Northwest regions, and abroad (5), including varieties specifically from Qinghai (7), Shanxi (6), Shaanxi (6), and abroad (5). Group 3, marked in blue in **Figure 7D** (35 varieties), encompasses varieties mainly from the Loess Plateau, and Northeast regions, including Shanxi (5), Shaanxi (4), and Heilongjiang (14).

For *K* = 5, the germplasms were divided into five groups. Group 1, marked in red in **Figure 7D** (27 varieties), consists of varieties predominantly from the Northeast, and Loess Plateau regions, comprising Heilongjiang (11), Shanxi (5), and Shaanxi (3). Group 2, marked in green in **Figure 7D** (20 varieties), includes varieties mostly sourced from the Loess Plateau region, featuring Shanxi (6), Ningxia (3), and Shaanxi (2). Group 3, marked in blue in **Figure 7D** (16 varieties), encompasses varieties primarily from the Northwest region, specifically Qinghai (5). Group 4, marked in yellow in **Figure 7D** (20 varieties), primarily comprises varieties mainly from the Loess Plateau region, and abroad, including Shaanxi (4), Ningxia (3), and abroad (5). Group 5, marked in purple in **Figure 7D** (64 varieties), contains varieties primarily from the Loess Plateau, and Inner Mongolia regions, comprising Shanxi (15), Shaanxi (12), and Inner Mongolia (10).

For *K* = 8, the germplasms were categorized into eight groups as illustrated in **Figure 7D**. Group 1, marked in red in **Figure 7D** (18 varieties), predominantly sourced from the Northeast, and Northwest regions. This includes varieties from Heilongjiang (8), and Gansu (4). Group 2, marked in green in **Figure 7D** (14 varieties), primarily consists of varieties mainly originating from the Loess Plateau region, including varieties specifically from Shanxi (6), Shaanxi (2), and Ningxia (2). Group 3, marked in blue in **Figure 7D** (16 varieties), encompasses varieties mainly from the Loess Plateau, Inner Mongolia, and Northwest regions, including Shanxi (3), Ningxia (3), Qinghai (3), and Inner Mongolia (3). Group 4, marked in yellow in **Figure 7D** (25 varieties), mainly comprises varieties from the Loess Plateau, and Northeast regions, with varieties specifically from Shanxi (6), Shaanxi (6), Ningxia (3), Heilongjiang (3), and Jilin (3). Group 5, marked in purple in **Figure 7D** (17 varieties), contains varieties primarily from the Loess Plateau, and Northeast regions, comprising Shaanxi (3), and Heilongjiang (5). Group 6, marked in aqua in **Figure 7D** (11 varieties), consists of varieties predominantly from the Northeast, and Loess Plateau regions, comprising Heilongjiang (4), and Shanxi (5). Group 7, marked in golden in **Figure 7D** (31 varieties), includes varieties mostly sourced from the Loess Plateau, Northwest, and Inner Mongolia regions, featuring Shanxi (8), Xiangjiang (4), Shaanxi (3), and Inner Mongolia (6). Finally, Group 8, marked in crimson in **Figure 7D** (15 varieties), primarily includes varieties from the Loess Plateau, and Northwest regions, specifically Qinghai (4), Shanxi (3), and Shaanxi (2).

### Construction of DNA fingerprints and molecular IDs

**Figure 8:**
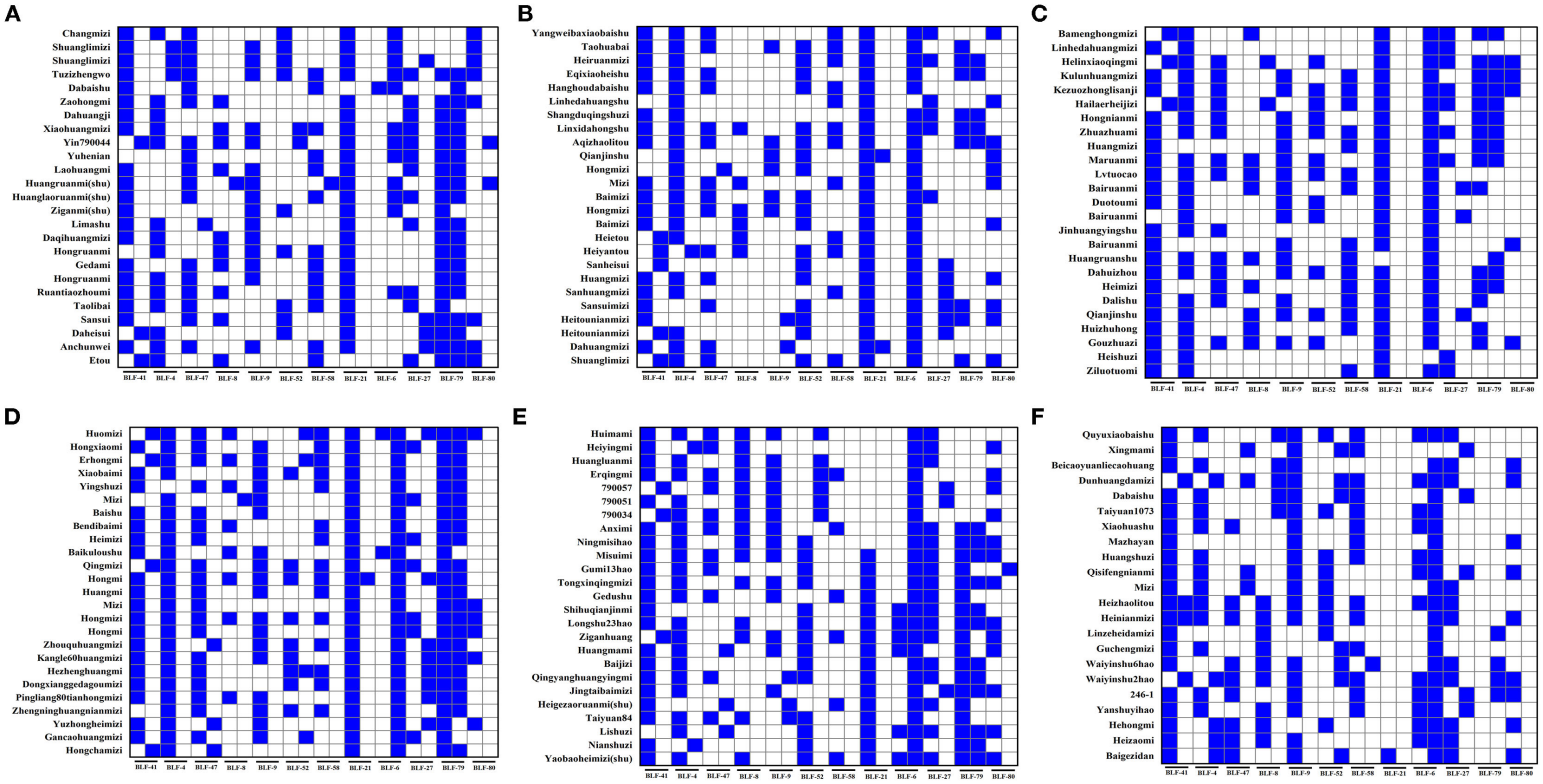
Fingerprints of proso millet germplasms. The labels **(A–F)** respectively represent the fingerprints of 147 proso millet germplasms in sequence. The blue grids represent a binary value of 1, whereas the white grids represent a binary value of 0.

**Figure 9:**
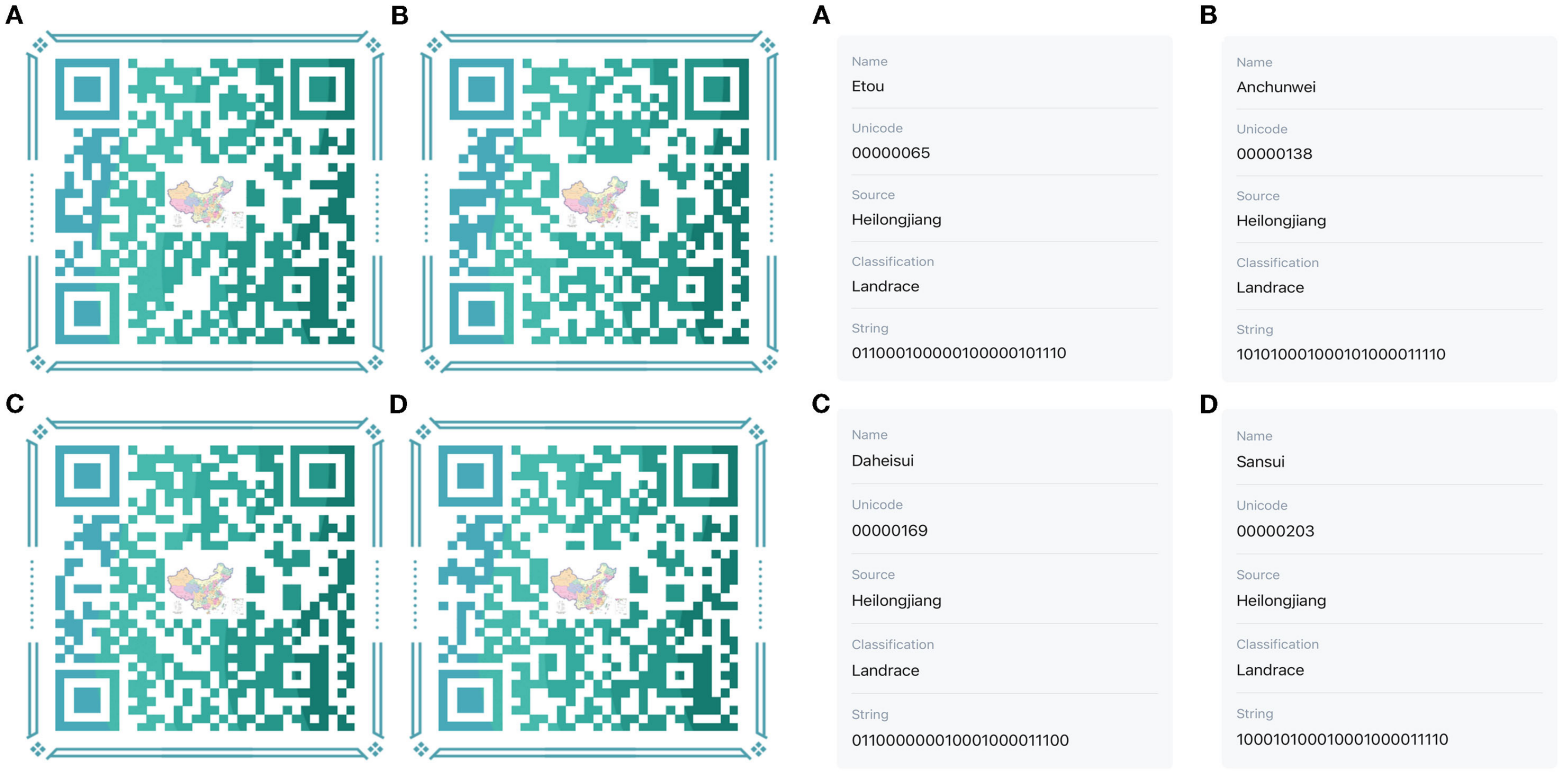
QR code DNA molecular IDs of proso millet germplasms of No1-4. The labels **(A–D)** on the left represent the molecular IDs of proso millet germplasms of No1-4 in sequence, whereas those on the right represent the scanning results of these molecular IDs.

To facilitate the conservation of the genetic resources of proso millet varieties, fingerprint maps were constructed for all 147 evaluated superior varieties on the basis of the SSR marker electrophoresis results (**Figure 8**). The electrophoresis results were digitally encoded, and following the principle of distinguishing the maximum number of varieties with the minimum number of primers, all amplification outcomes of the assayed varieties were combined in the following order: BLF41, BLF4, BLF47, BLF8, BLF9, BLF52, BLF58, BLF21, BLF66, BLF-27, BLF-79, and BLF80. This procedure produced a distinct 24-character string for each variety (**Supplementary Table S12**). These strings of DNA molecular IDs were then augmented with additional information such as Name, Unicode, Source, and Classification to create QR code DNA molecular IDs using online QR code generation technology. QR code DNA molecular IDs for varieties 1, 2, 3, and 4 are presented in **Figure 9**, while those for the remaining 143 varieties are detailed in **Supplementary Figure S1**.

## Discussion

In proso millet, morpho-agronomic traits, which are important for its genetic improvement, include plant height, panicle length, thousand-grain weight, and grain weight per plant (Rajput et al., 2013; 2024). In several cereal crops, many of such traits demonstrated high genetic variability and were used in breeding programs for genetic improvement (Gaju et al., 2009; Upadhyaya et al., 2011; Pucher et al., 2015). In the present study, the variation coefficients for these various traits ranged from 5.22 to 60.03% (**Figures 2**,**3**, **4**), and the diversity index for these traits ranged from 0.15 to 2.10 (**Supplementary Table S9**), with larger values noted for panicle length, stem height, stem diameter, panicle weight per plant, grain weight per plant, and thousand-grain weight, all exceeding 2, in agreement with the findings of Zhang et al. (2019). The variation coefficients and diversity indices in this study were notably high, demonstrating significant variation among the germplasm resources and indicating substantial genetic diversity. Hence, these resources are valuable in breeding programs and parental selection processes.

Correlation analysis between various morpho-agronomic traits is necessary for direct and indirect selection and genetic improvement of crops, which may help breeders to identify traits that are quickly and easily measurable, making them very useful in selections (Rajput et al., 2024). The present study revealed significant or highly significant positive and negative correlations between multiple traits (**Figure 5**). As a physiological process, plant height is highly correlated with the number of nodes; an increase in the number of nodes corresponds to an increase in plant height (**Figure 5**), aligning with the findings of Zhang et al. (2019) and Rajput et al. (2024). Notably, this study found no correlation between the tiller number and grain weight per plant or thousand-grain weight (**Figure 5**), diverging from the findings of Guo et al. (2025); this discrepancy is likely attributable to environmental factors, as this present research was confined to a single year and location. Principal component analysis reduced 12 indicators into four principal components, with a cumulative contribution rate of 73.78% (**Supplementary Table S10**), effectively describing trait variation. Major traits contributing to this variation include tiller number, panicle length, and thousand-grain weight, consistent with the findings of Yazdizadeh et al. (2020). Clustering by principal component analysis involves the decomposition of phenotypic data into ordered, unrelated, orthogonal principal components based on the Euclidean distance matrix (Odong et al., 2013). Simply put, PCA-based clustering improves the extraction of genetically relevant information from many quantitative variables to a limited number of variables (Rajput et al., 2024). In the present study, cluster analysis divided the 1,582 germplasms into five categories, finding that proso millet from each of the different ecological zones may be grouped together (**Figure 6**).

It is inevitable that the present study still has certain limitations, as it relies on data from a single year and location and thus may not fully account for environmental variability or long-term trends. Nonetheless, our findings are consistent with previous studies (Zhang et al., 2019; Rajput et al., 2024; Yazdizadeh et al., 2020), which effectively confirms the reliability of the present results. Future investigations incorporating diverse temporal and spatial scales will improve the study’s applicability and relevance.

The Shannon diversity index (*I*) and polymorphism information content (PIC) comprehensively reflect population genetic diversity levels (Liu and Muse, 2005). Generally, primers with PIC values less than 0.25 indicate low polymorphism, PIC values greater than or equal to 0.25 and less than 0.5 indicate moderate polymorphism, and PIC values greater than or equal to 0.5 indicate high polymorphism (Sharma et al., 2021). Most primers used in this study demonstrated moderate to high polymorphism (**Table 1**), making them suitable for genetic diversity analysis. This study assessed genetic differences in 147 proso millet germplasms, finding Shannon diversity indices ranging from 0.07 to 0.68, with an average of 0.33 (**Table 1**), which was lower than those reported by Yazdizadeh et al. (2020); Liu et al. (2017, 2016), Hunt et al. (2011), and Cho et al. (2010). PIC values in this study ranged from 0.17 to 0.59, averaging 0.38 (**Table 1**), higher than those reported by Hunt et al. (2011); Cho et al. (2010), and Liu et al. (2016), but lower than those reported by Hu et al. (2009), and Yazdizadeh et al. (2020), possibly owing to differences in experimental materials or SSR marker types used. With respect to accession source, the Shannon diversity index and PIC values for varieties from the Northwest, and Loess Plateau regions were higher than those from other regions (**Table 2**), indicating greater genetic diversity in these areas, consistent with the findings of Hu et al. (2009). Furthermore, the genetic distance between varieties from the Northeast and Northwest regions was closest, with the highest genetic consistency (**Table 3**), consistent with a positive correlation between geographic origin and genetic distance (Hu et al., 2009), contrasting with the findings of Hou et al. (2017); this discrepancy may arise from the differences in marker types used or the specific properties of the markers selected.

Cluster analysis based on UPGMA is an effective method for evaluating the genetic diversity and background of crop germplasm resources, which are essential for the effective conservation and utilization of these resources (Rajput et al., 2024). The cluster analysis performed in this study revealed that all five foreign varieties were classified into the third group (**Figure 7A**, and **Table 4**), suggesting that the grouping may be associated with geographical origin. However, occasionally exceptions can occur in groupings based on UPGMA, as has been observed across major cereal crops, and principal component analysis can provide a reasonable explanation for this discrepancy (Van Inghelandt et al., 2010; Courtois et al., 2012; Bonman et al., 2015). In the present study, UPGMA clustering categorized varieties from Northeast China into three groups (**Figure 7A**), whereas principal component analysis classified them into a single group (**Figure 7B**). The individual cases appearing in the cluster analysis groups are all classified into appropriate groups in PCA, reasonably explaining the aforementioned classification issues.

## Conclusion

Proso millet germplasm resources exhibit substantial phenotypic and genetic variation, with the greatest variation coefficients in yield traits. A total of 10 high-performing germplasms were identified, which can meet the needs of germplasm resource development and genetic breeding. Additionally, 15 polymorphic markers were developed, enabling the precise evaluation of 147 proso millet accessions from both regions within China and foreign sources. Proso millet germplasm resources originating from China were observed to have abundant genetic diversity, generally higher than those from abroad. Meanwhile, efforts must be intensified to collect wild varieties and introduce foreign varieties to enhance the genetic diversity among existing resources, thereby promoting research on the genetic evolution and improvement of proso millet. In addition, this study will be followed by long-term research across various locations to ensure the validity and reliability of the present results.

## Supporting information

The following supporting information is available online: **Supplementary Tables S1–S12**, **Supplementary Data S1–S2**, and **Supplementary Figures S1-S4**.

## Data Availability

The datasets presented in this study can be found in online repositories. The names of the repository/repositories and accession number(s) can be found in the article/**Supplementary Material**.
