# Supplementary figures and images for: Genetic diversity analysis of proso millet (Panicum miliaceum L.) germplasm resources based on phenotypic traits and SSR markers
Source: Front Plant Sci. 2025 Sep 8;16:1649200. doi: 10.3389/fpls.2025.1649200 (PMC12450881; doi:10.3389/fpls.2025.1649200)

**
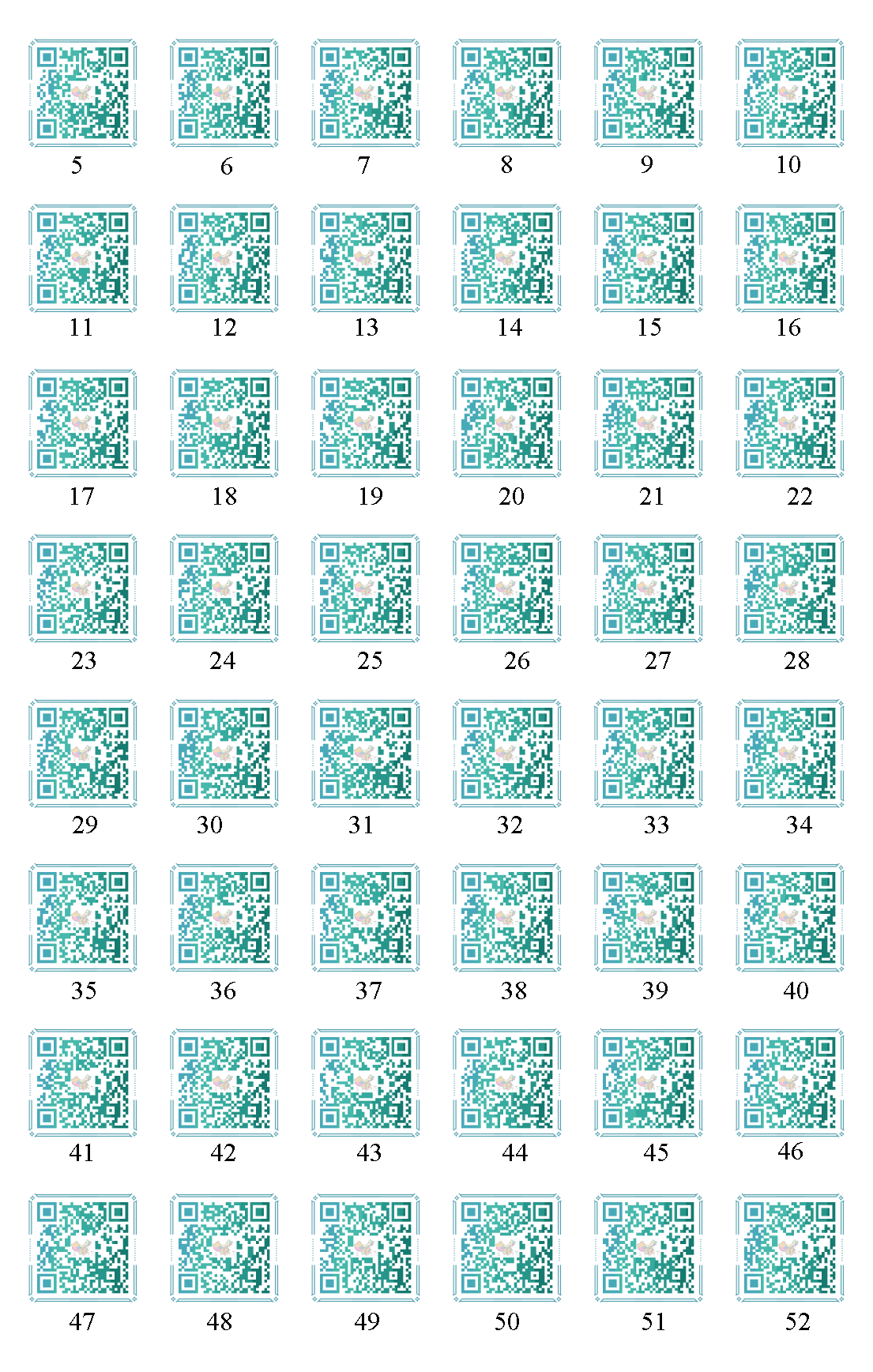
Figure S1 QR code DNA molecular IDs of proso millet germplasms of No 5-147.**


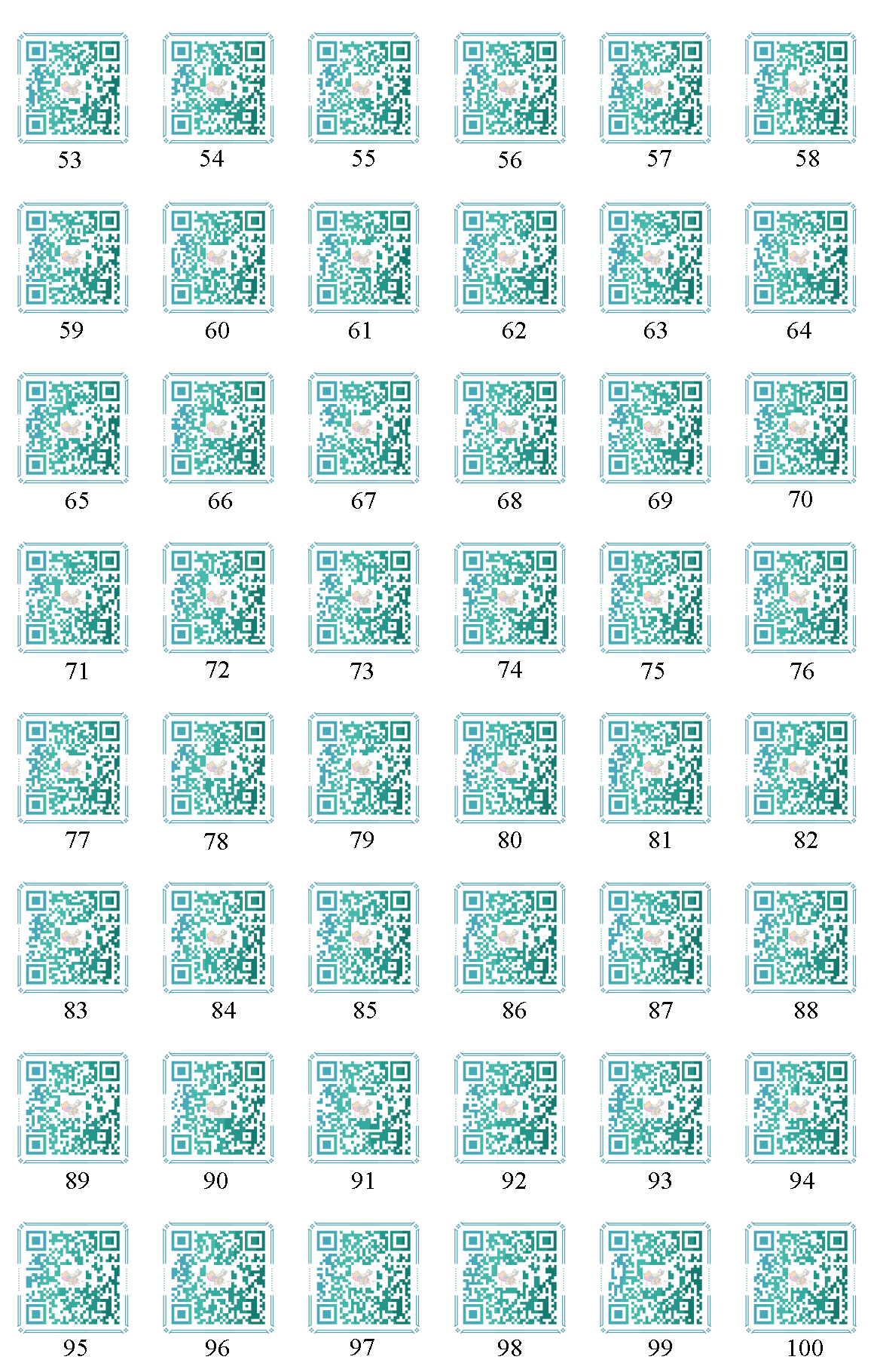

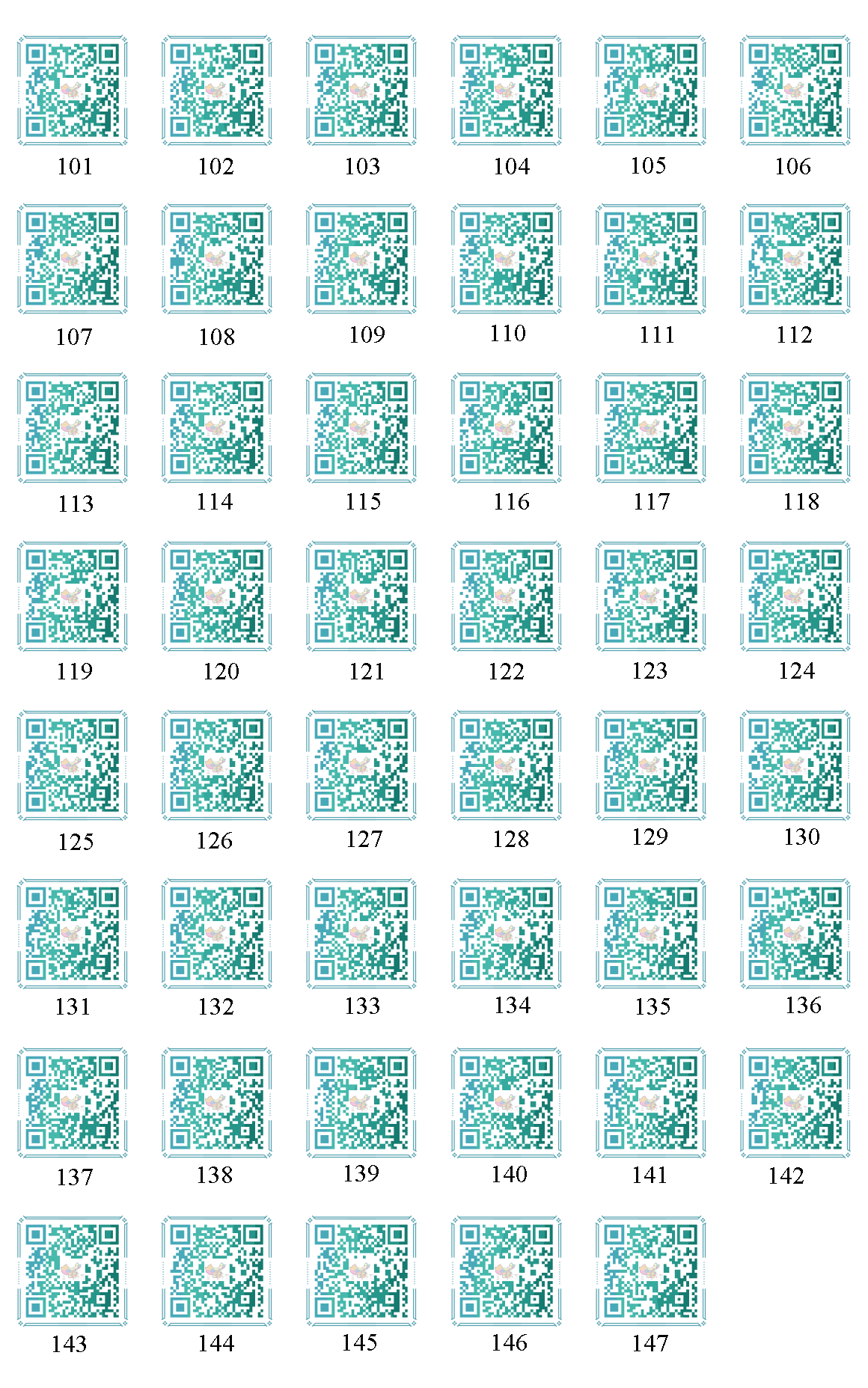

Supplement: Supplementary file 1 [file Table1.docx]
