# Supplementary material for: Genetic diversity analysis of proso millet (Panicum miliaceum L.) germplasm resources based on phenotypic traits and SSR markers
Source: Front Plant Sci. 2025 Sep 8;16:1649200. doi: 10.3389/fpls.2025.1649200 (PMC12450881; doi:10.3389/fpls.2025.1649200)
Supplement: Supplementary file 2 [file Table2.docx]

**Figure S2 Amplification bands of 80 SSR markers for polymorphism screening.**

| 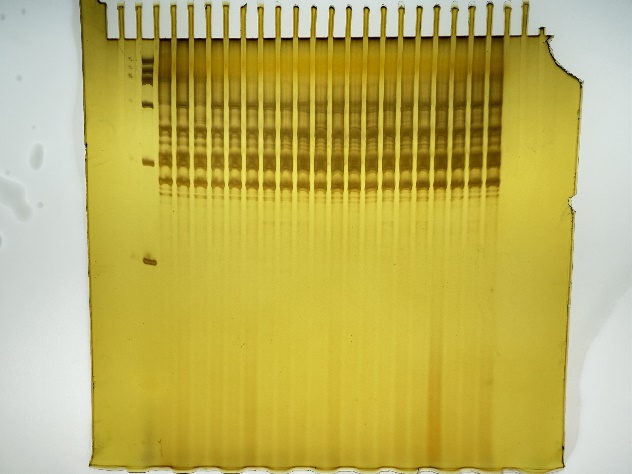BLF-1 | **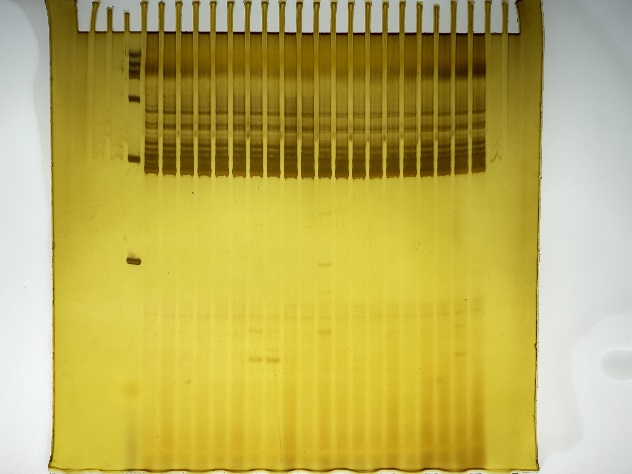**BLF-2 |
| --- | --- |
| **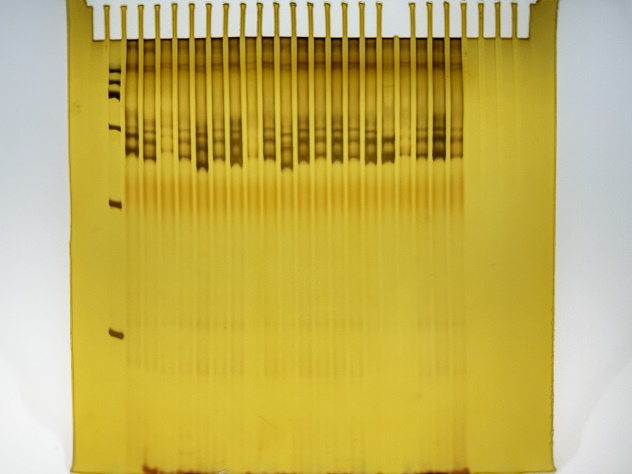**BLF-3 | **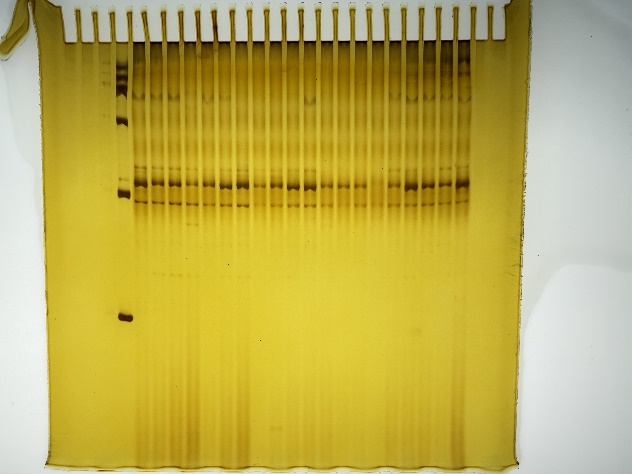**BLF-4 |
| **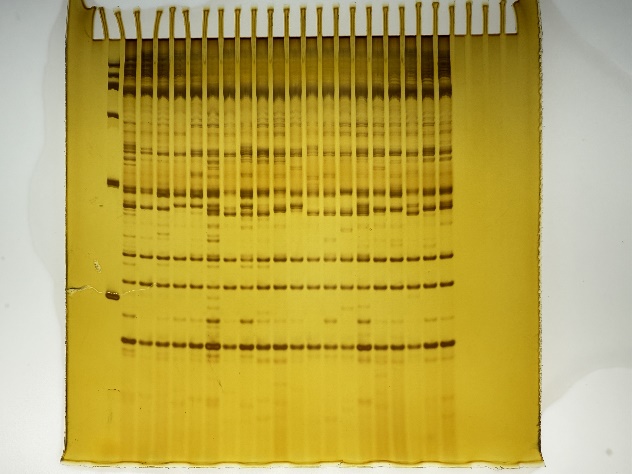**BLF-5 | **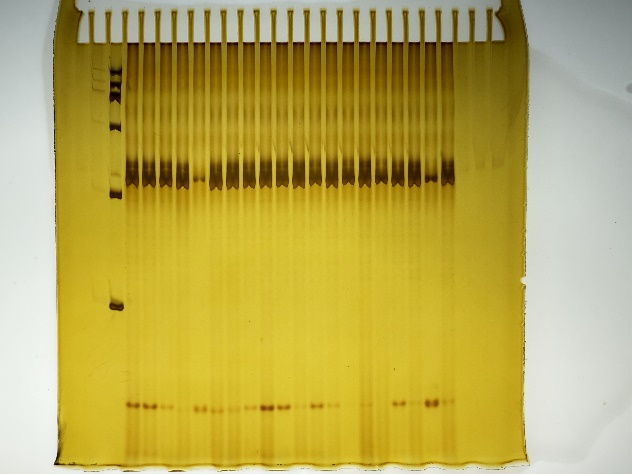**BLF-6 |
| BLF-**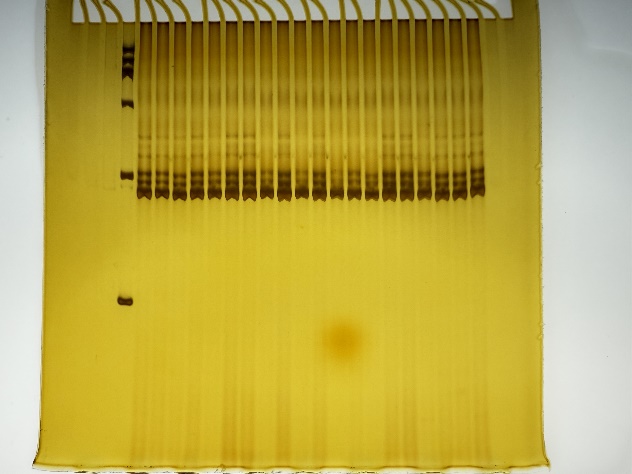**7 | BLF-**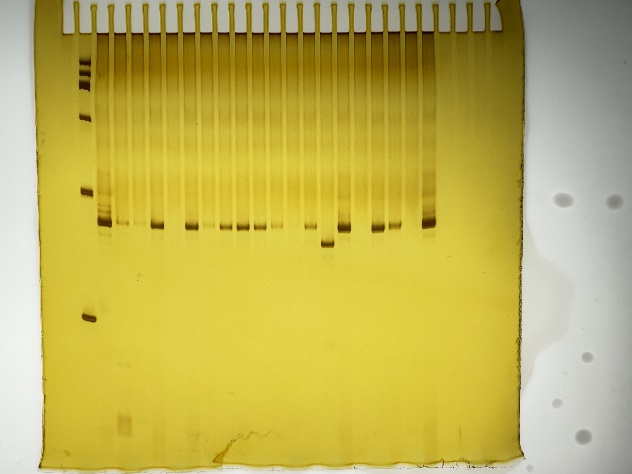**8 |
| BLF-**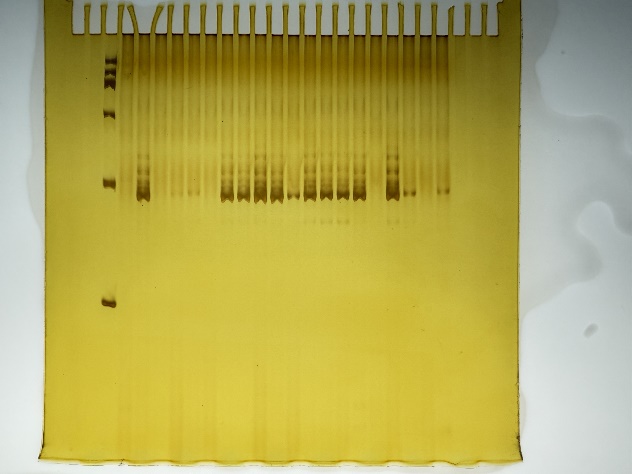**9 | BLF-**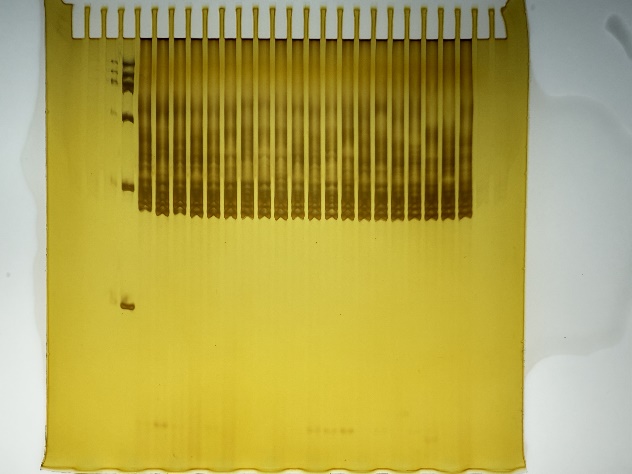**10 |
| **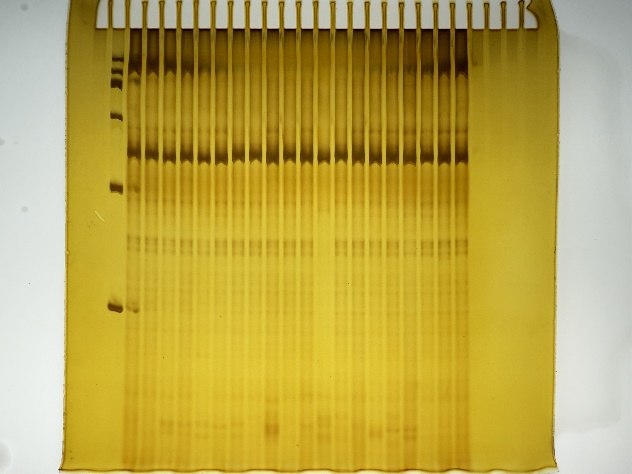**BLF-11 | BLF-**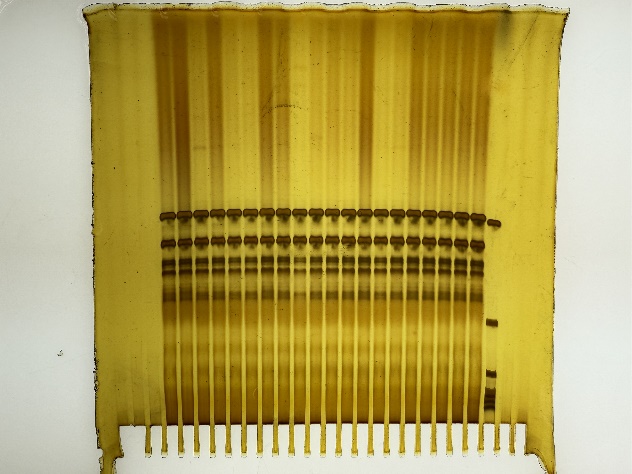**12 |
| BLF-**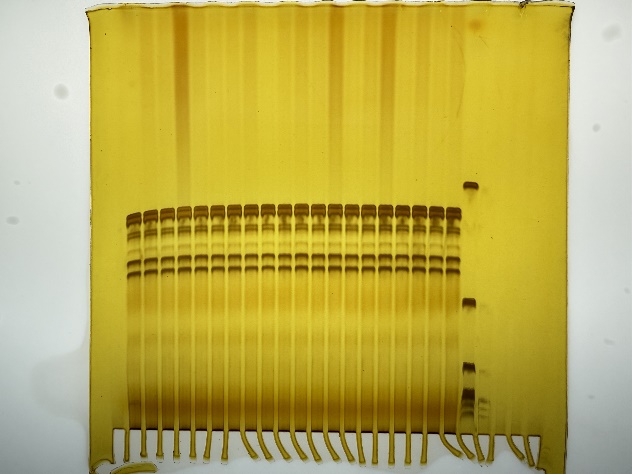**13 | **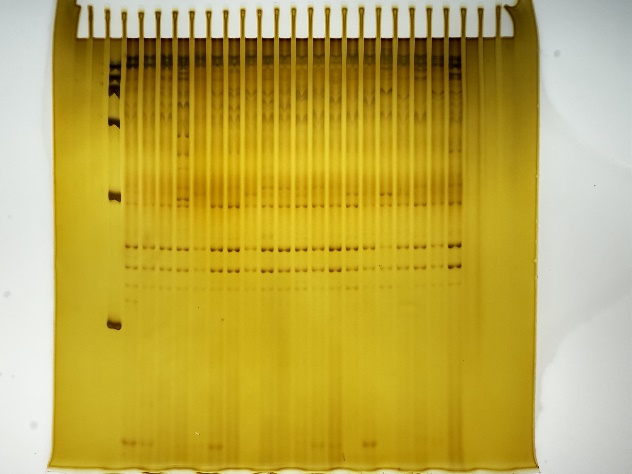**BLF-14 |
| BLF-**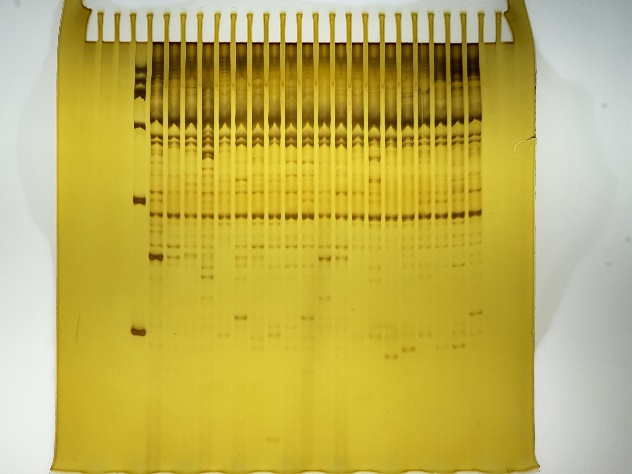**15 | BLF-**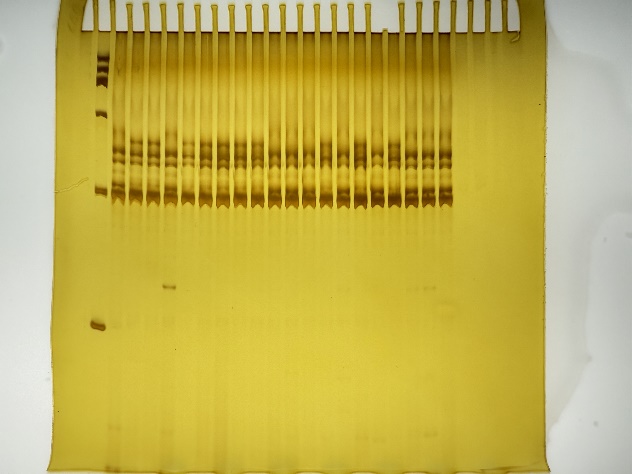**16 |
| BLF-**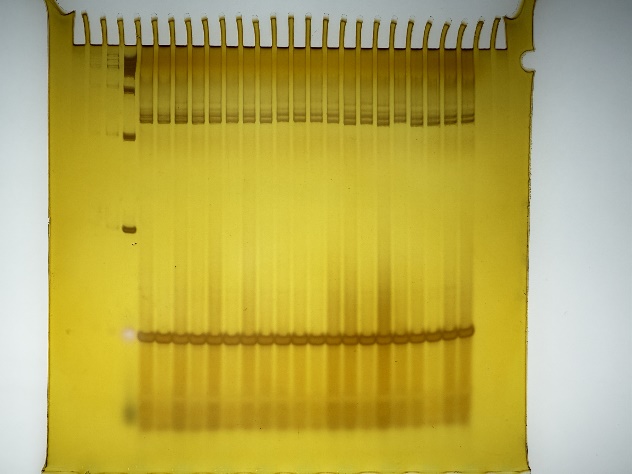**17 | BLF-**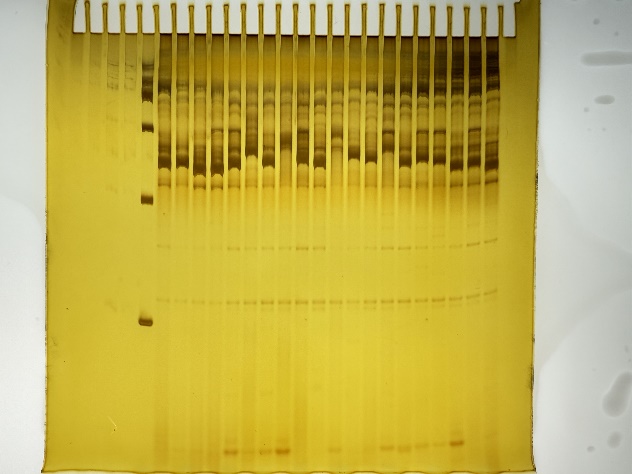**18 |
| BLF-**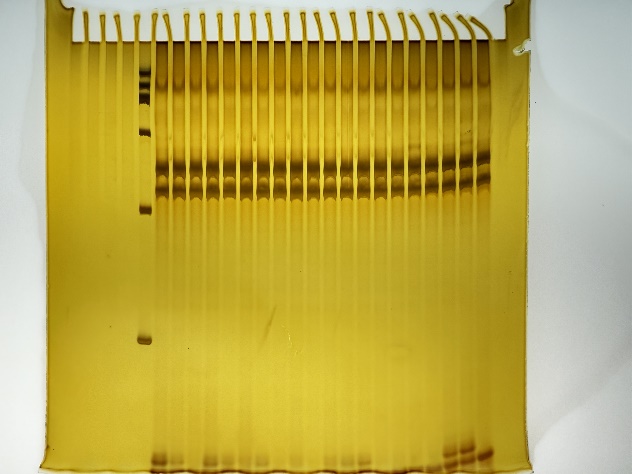**19 | BLF-**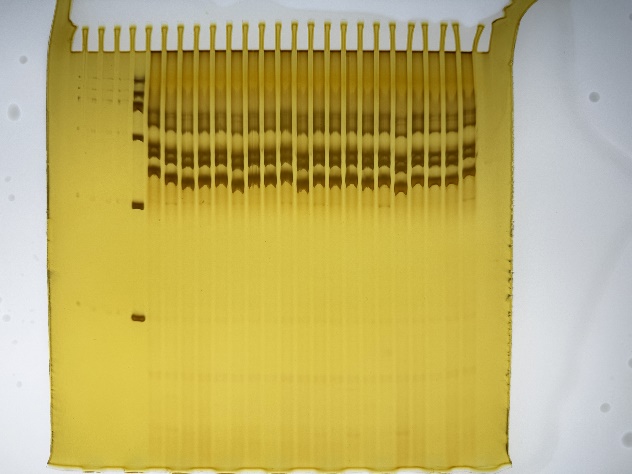**20 |
| BLF-**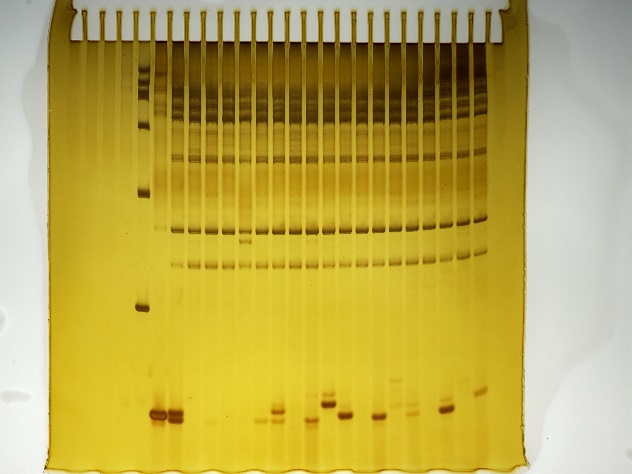**21 | BLF-**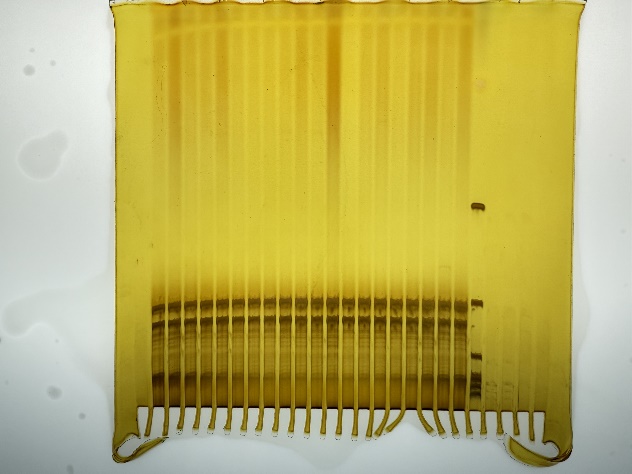**22 |
| BLF-**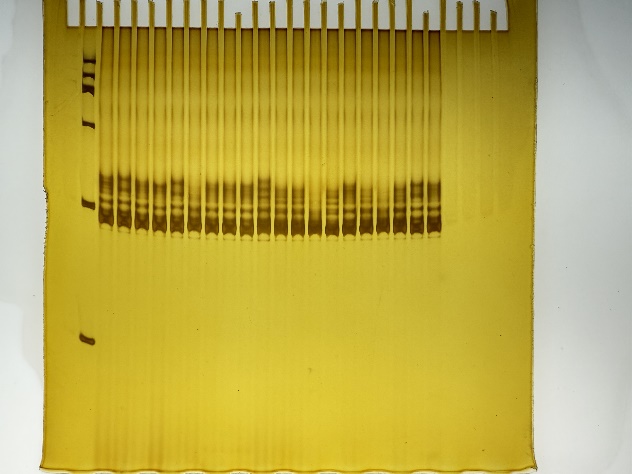**23 | BLF-**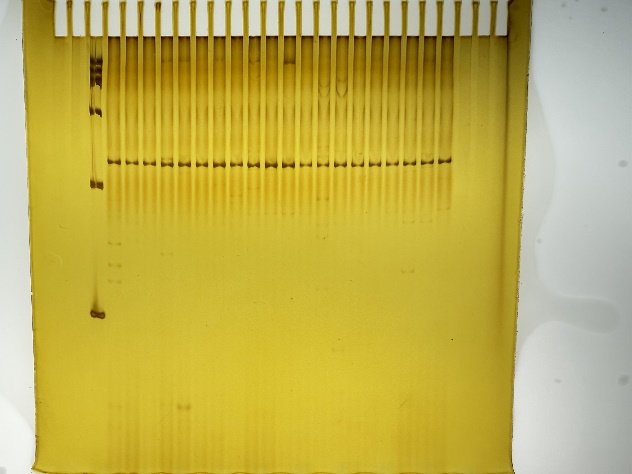**24 |
| BLF-**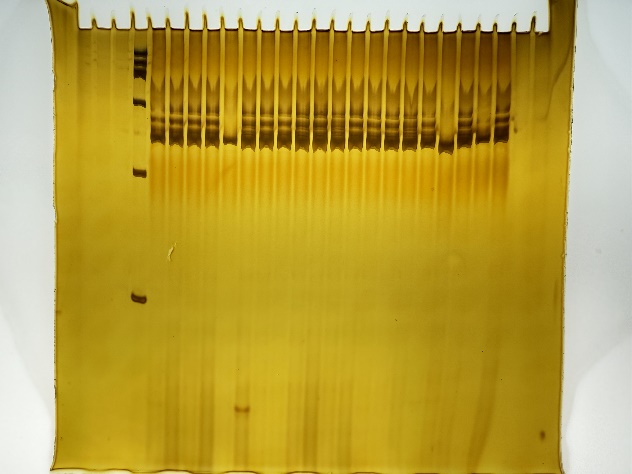**25 | BLF-**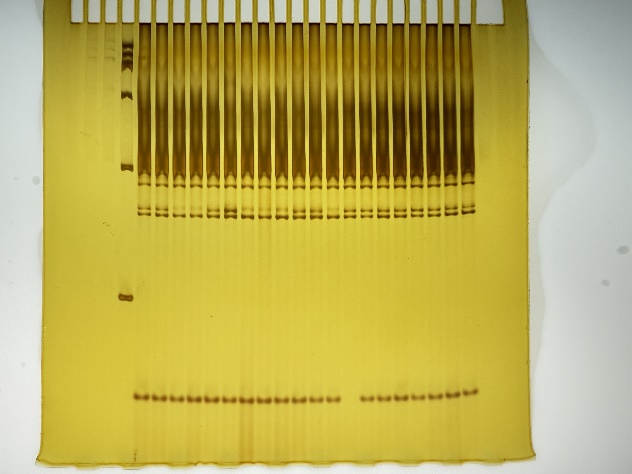**26 |
| BLF-**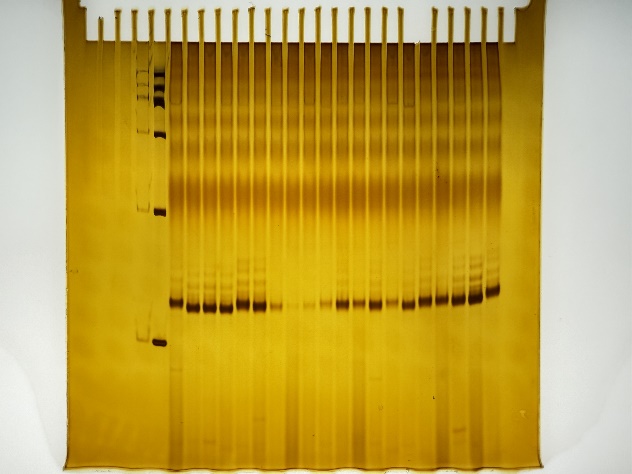**27 | BLF-**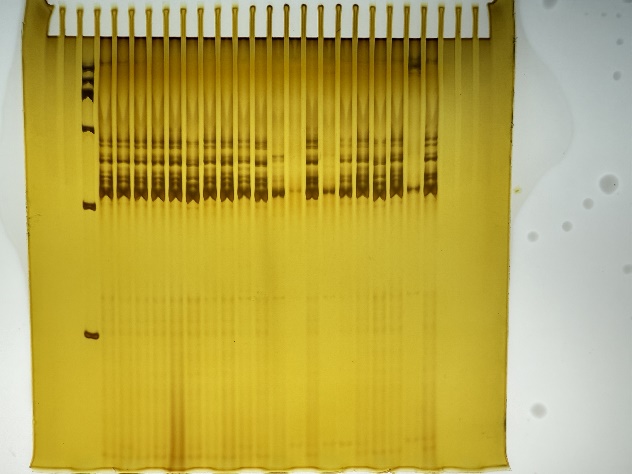**28 |
| BLF-**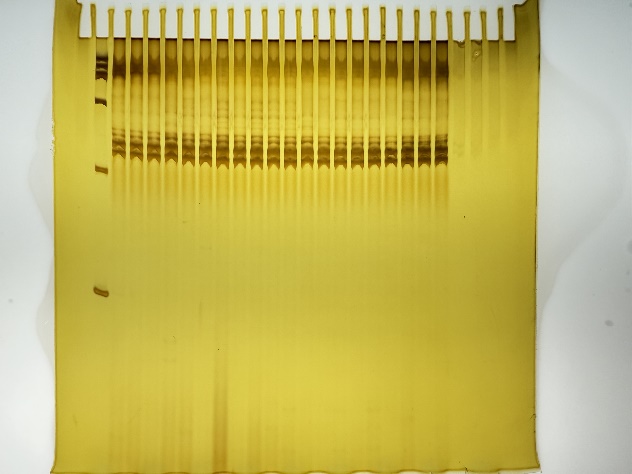**29 | BLF-**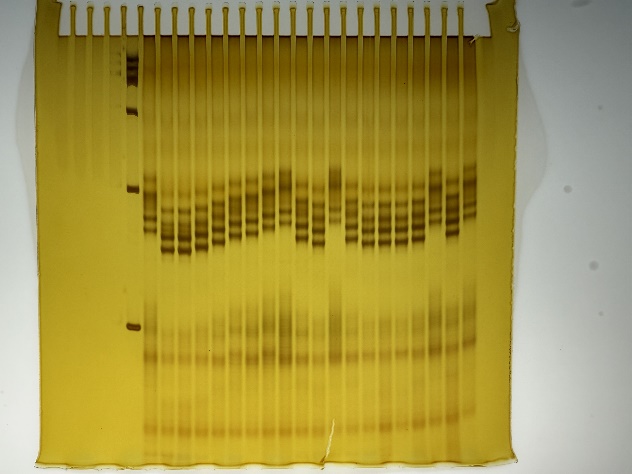**30 |
| BLF-**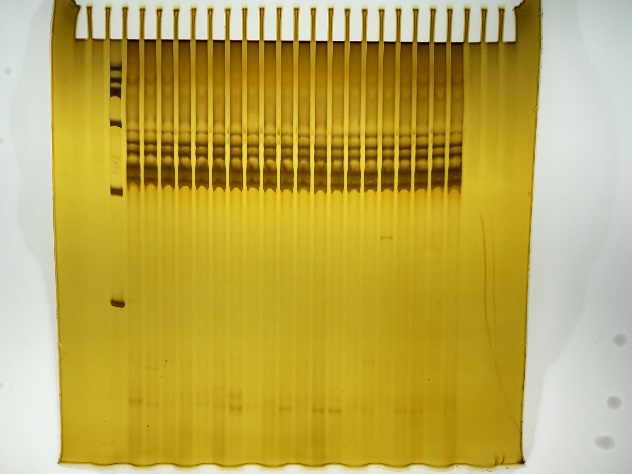**31 | BLF-**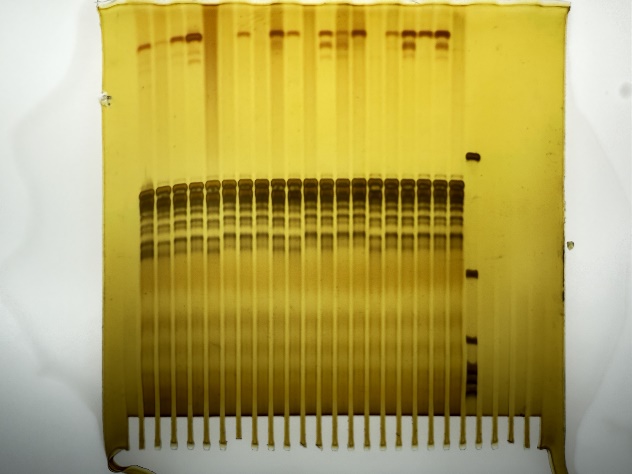**32 |
| BLF-**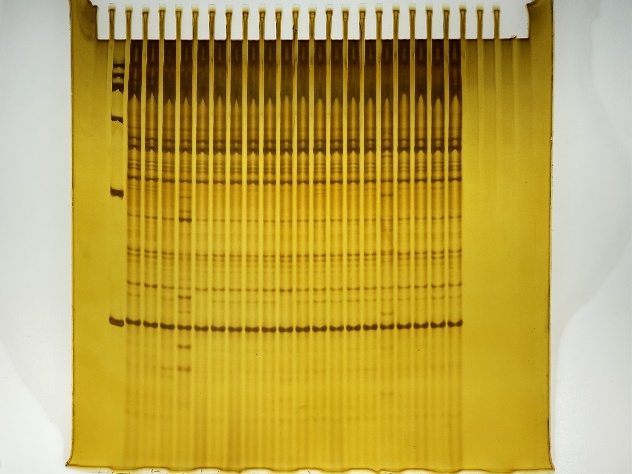**33 | BLF-**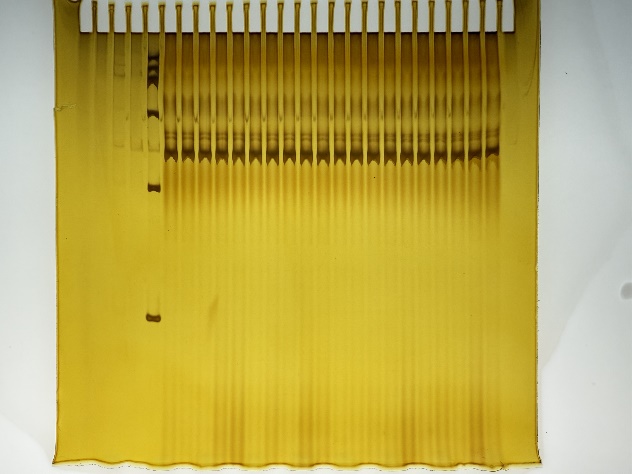**34 |
| BLF-**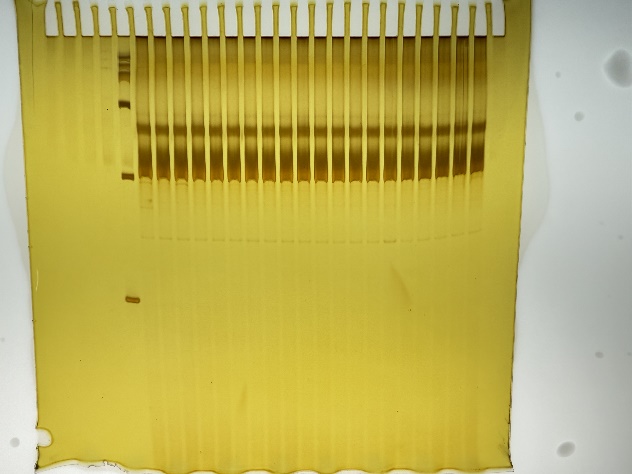**35 | BLF-**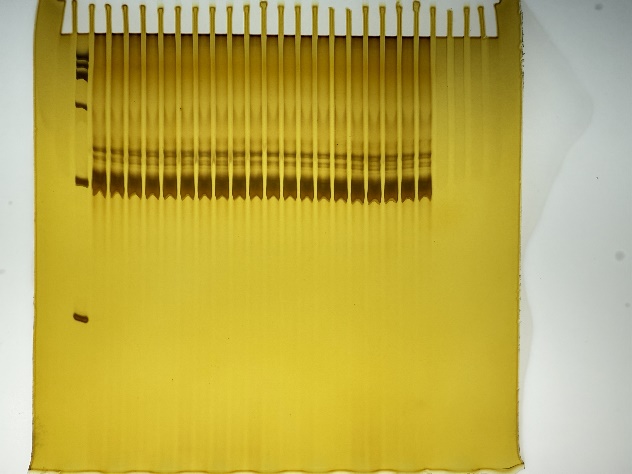**36 |
| BLF-**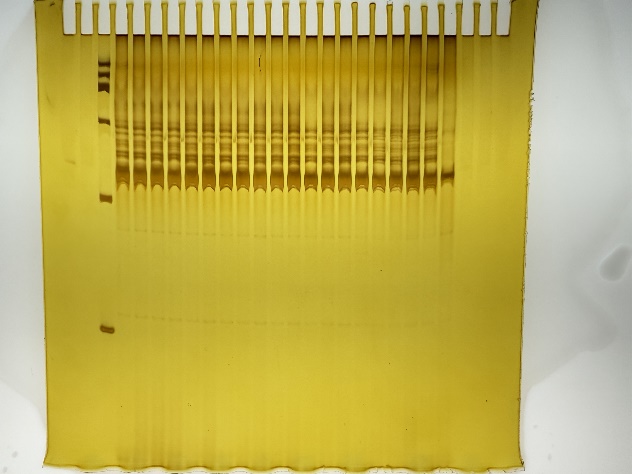**37 | BLF-**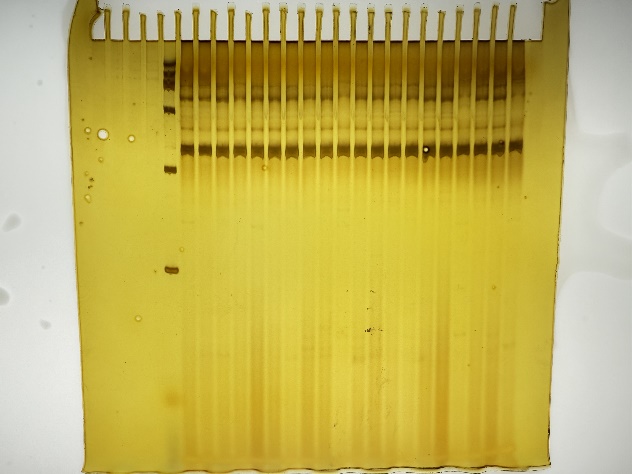**38 |
| BLF-**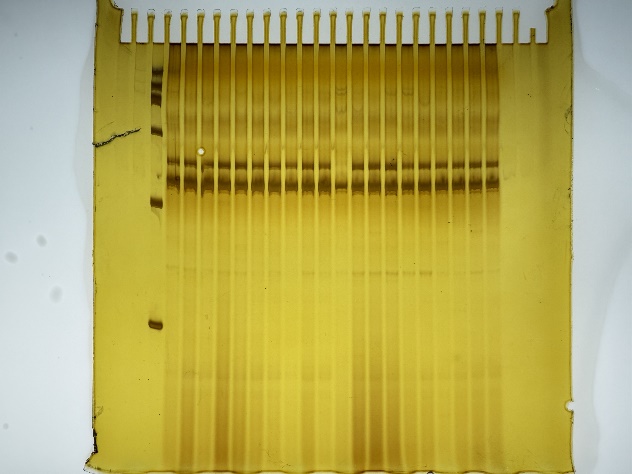**39 | BLF-**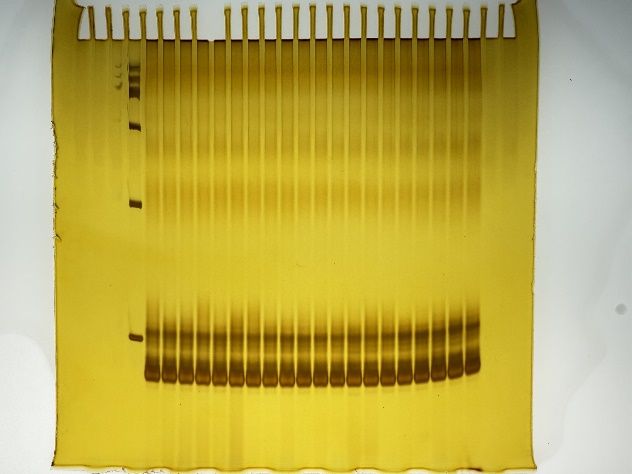**40 |
| BLF-**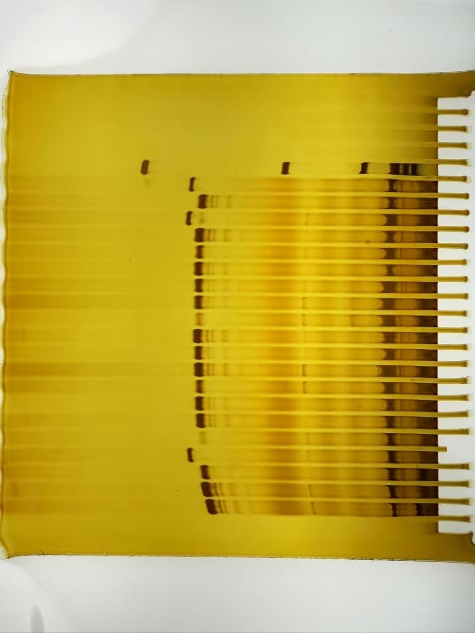**41 | BLF-**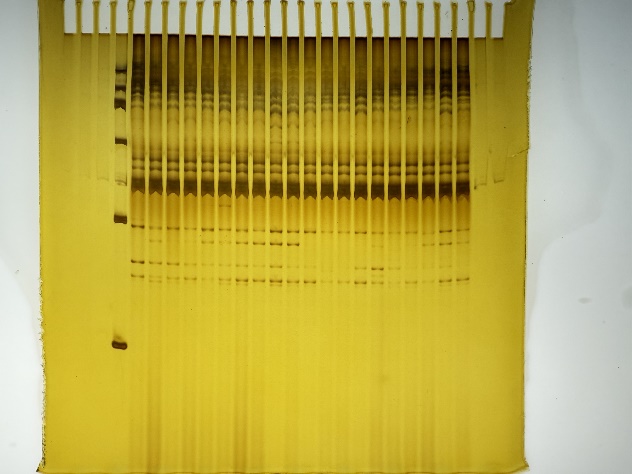**42 |
| BLF-**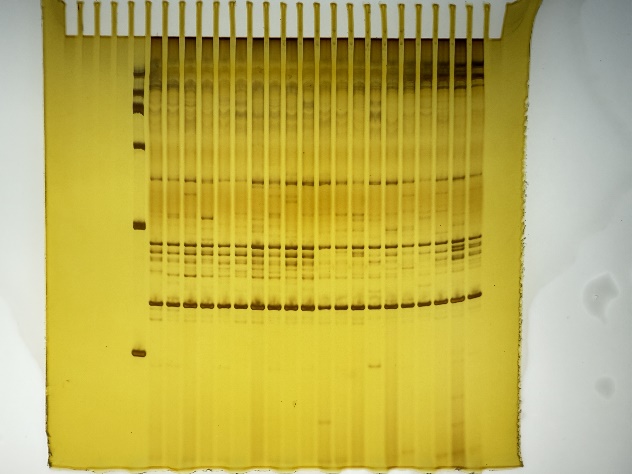**43 | BLF-**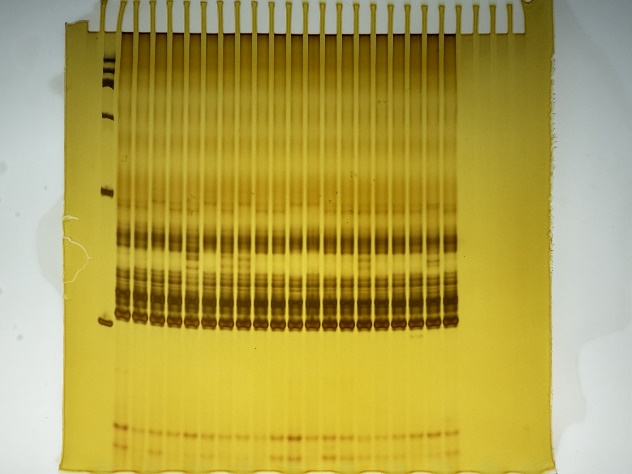**44 |
| BLF-**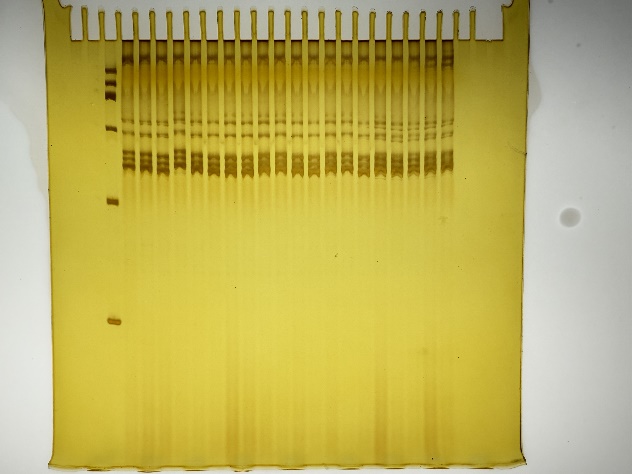**45 | BLF-**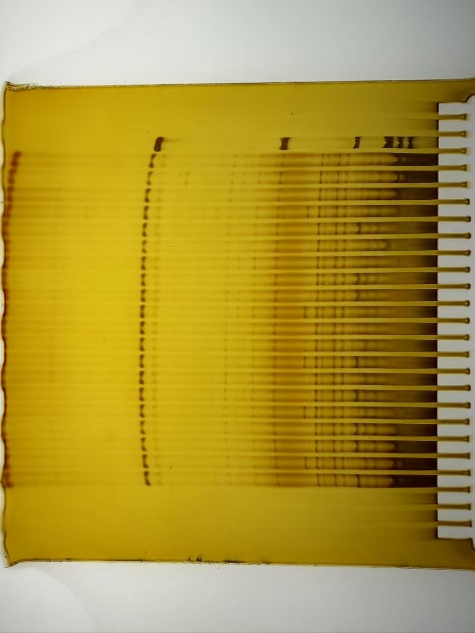**46 |
| BLF-**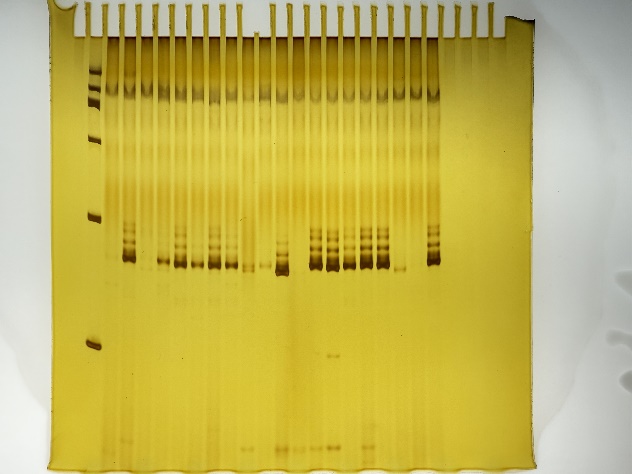**47 | BLF-**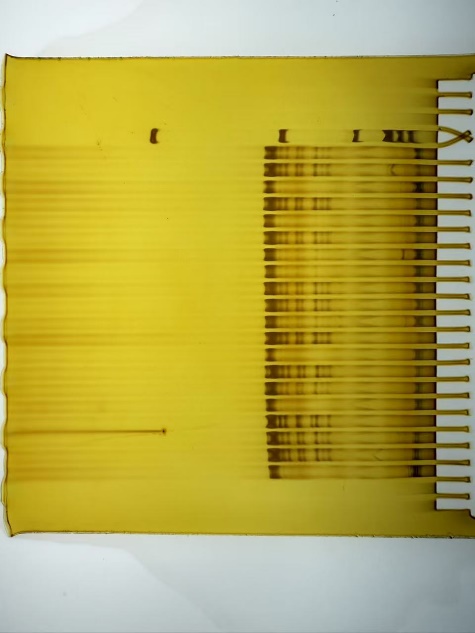**48 |
| BLF-**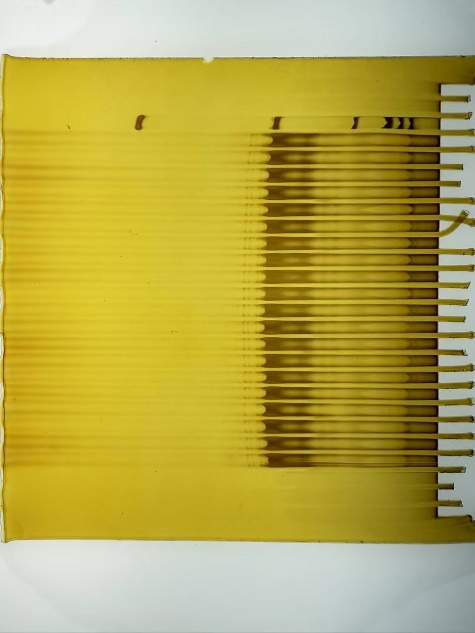**49 | BLF-**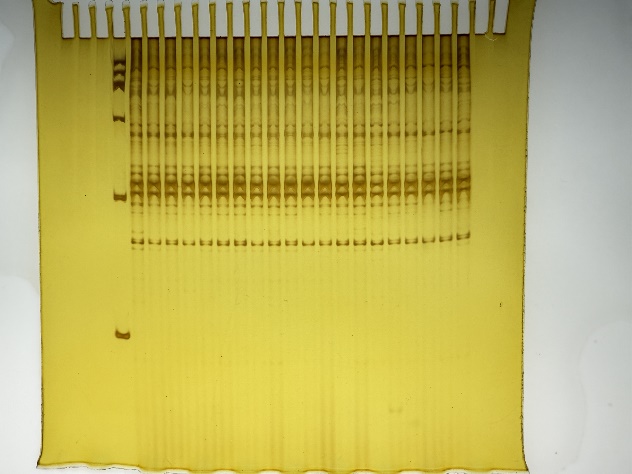**50 |
| BLF-**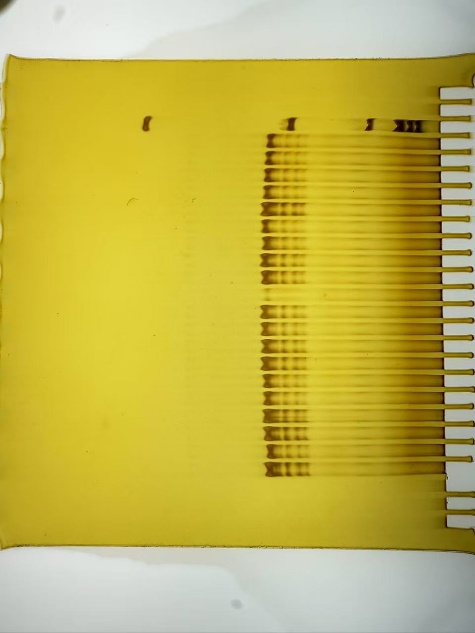**51 | BLF-**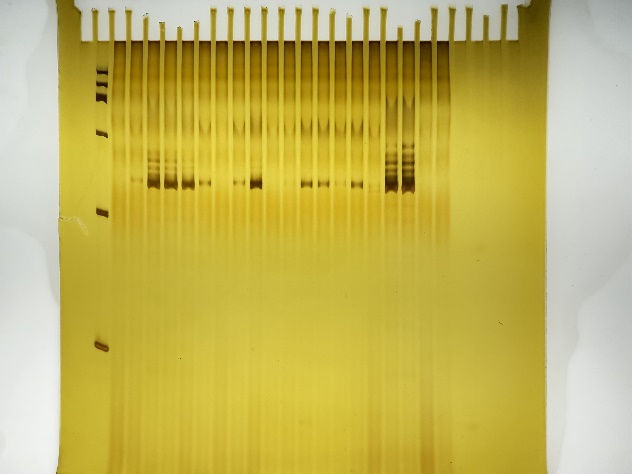**52 |
| BLF-**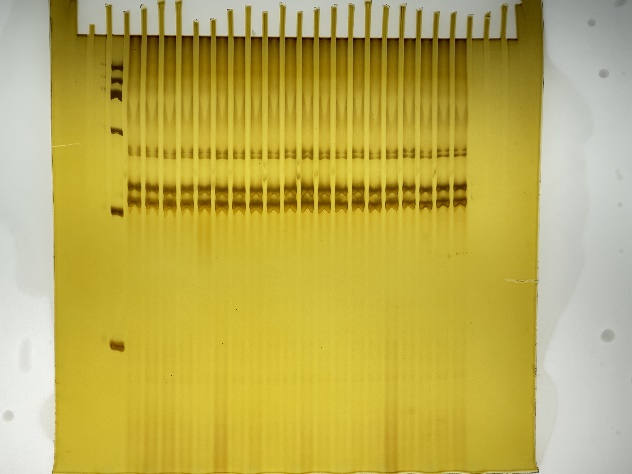**53 | BLF-**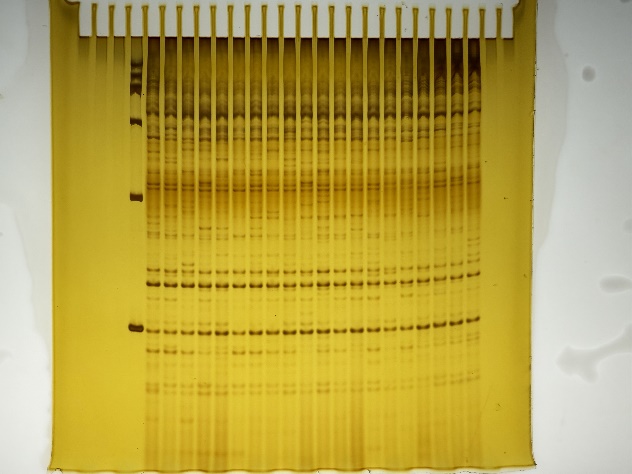**54 |
| BLF-**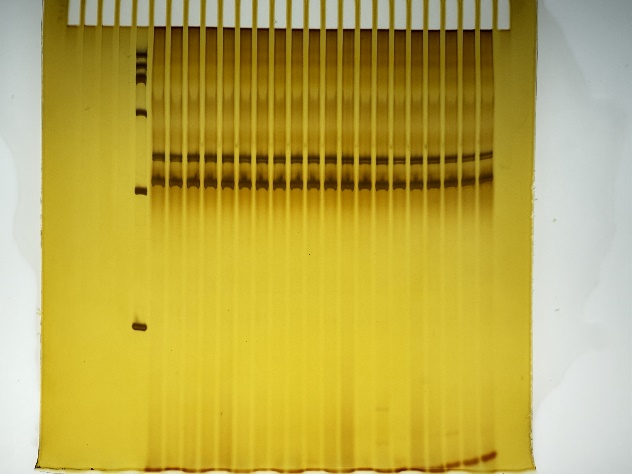**55 | BLF-**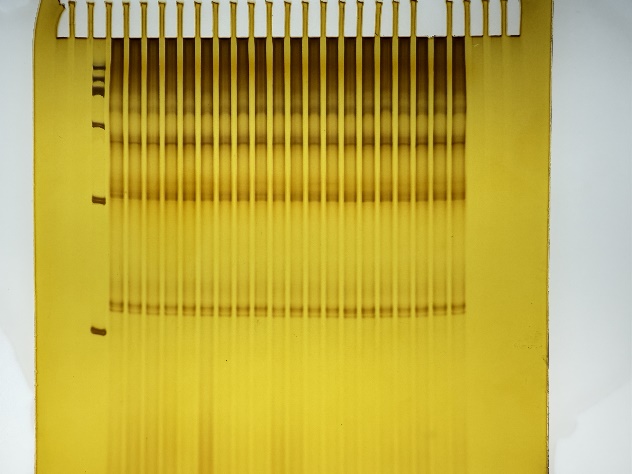**56 |
| BLF-**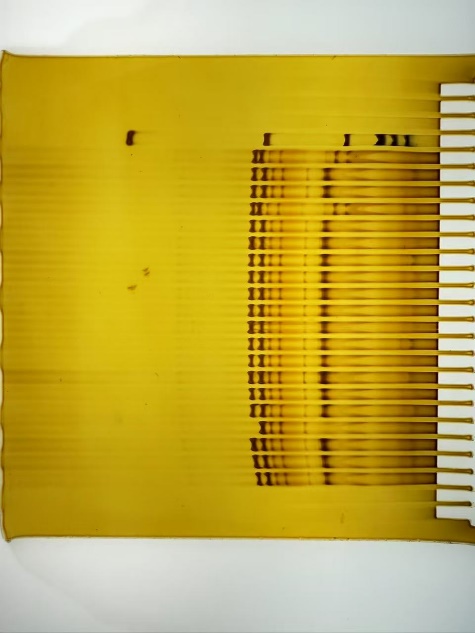**57 | BLF-**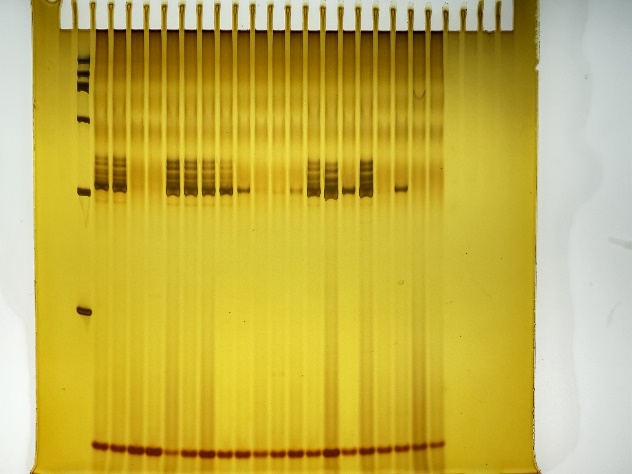**58 |
| BLF-**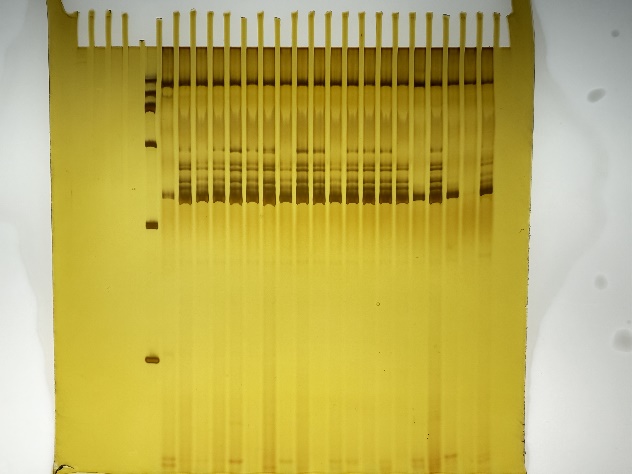**59 | BLF-**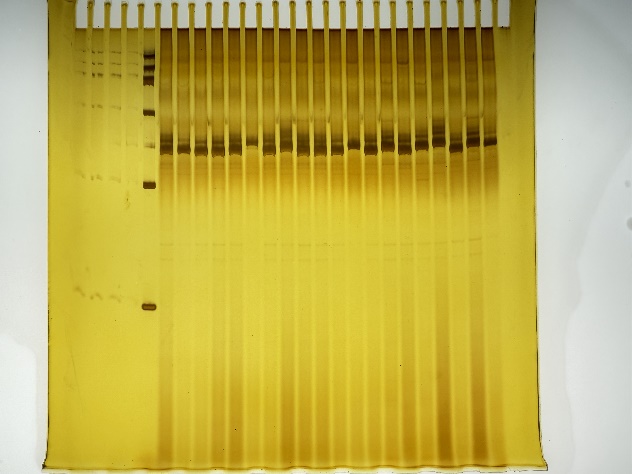**60 |
| BLF-**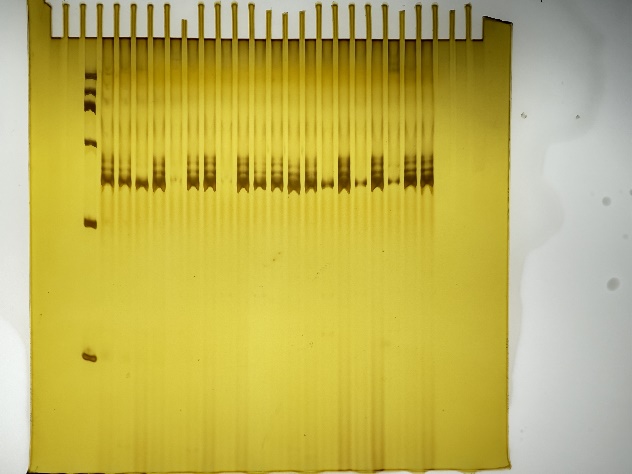**61 | BLF-**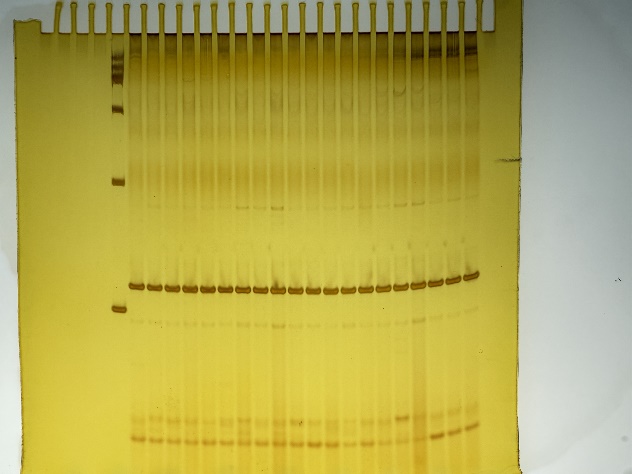**62 |
| BLF-**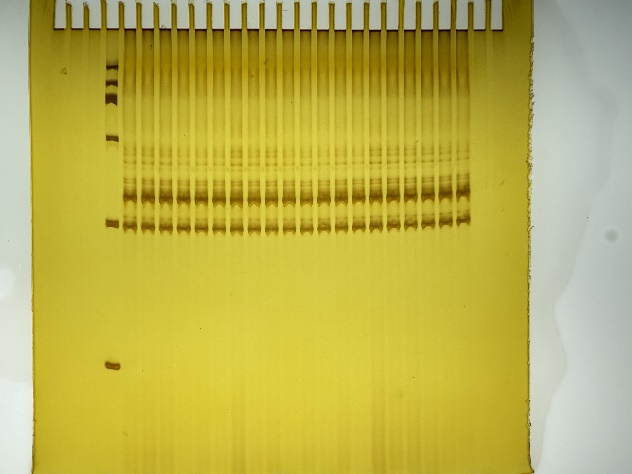**63 | BLF-**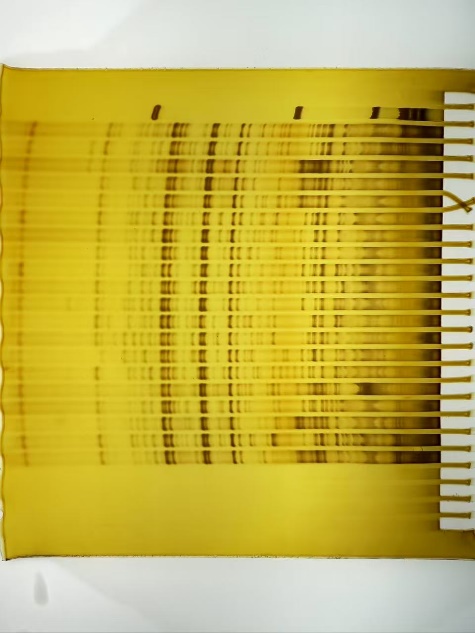**64 |
| BLF-**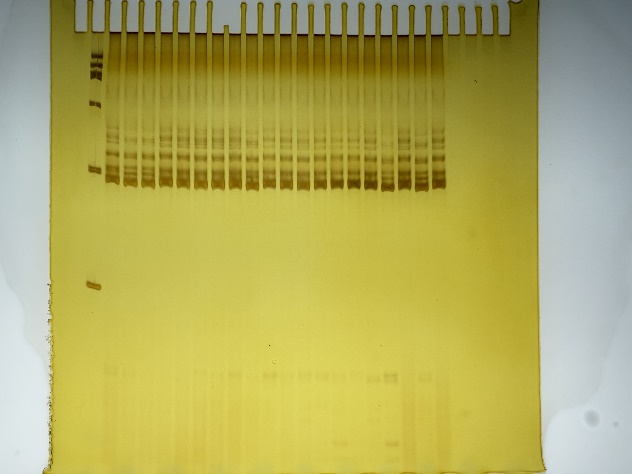**65 | BLF-**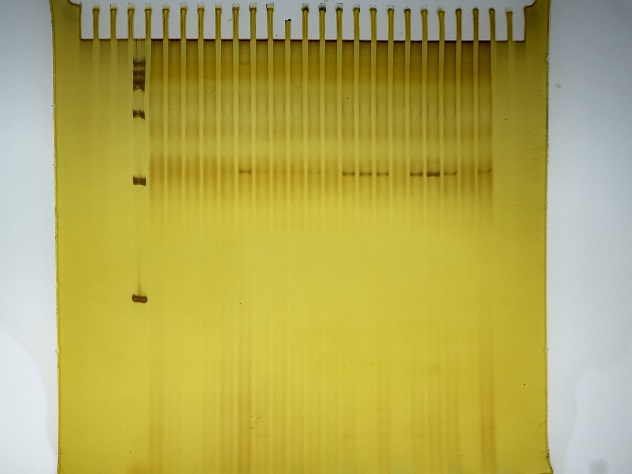**66 |
| BLF-**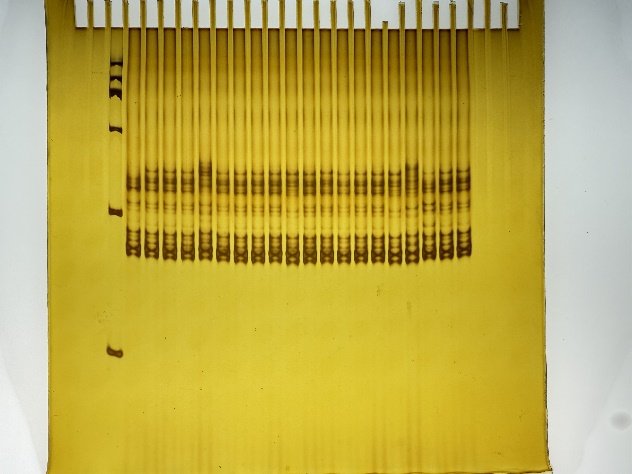**67 | BLF-**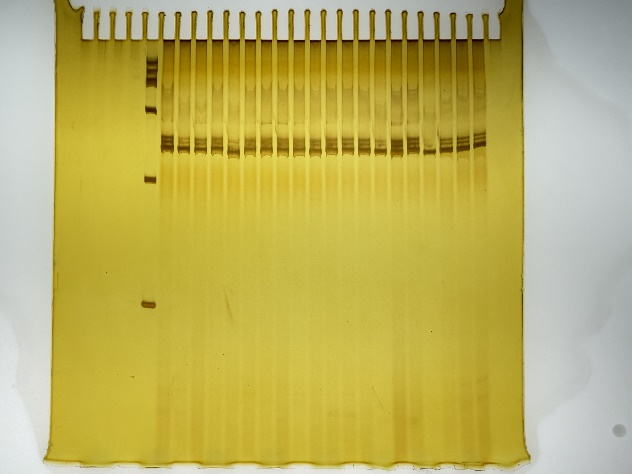**68 |
| BLF-**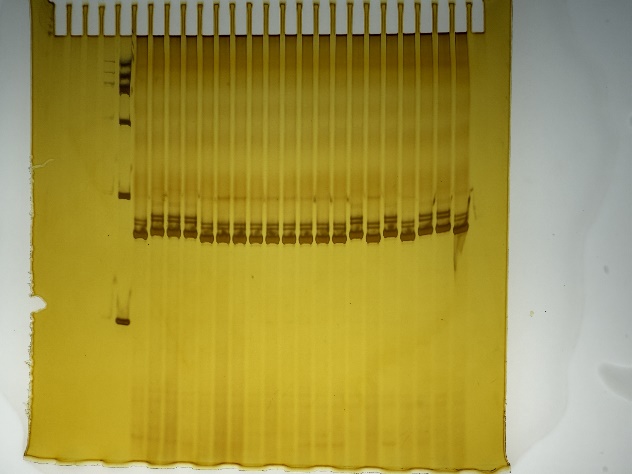**69 | BLF-**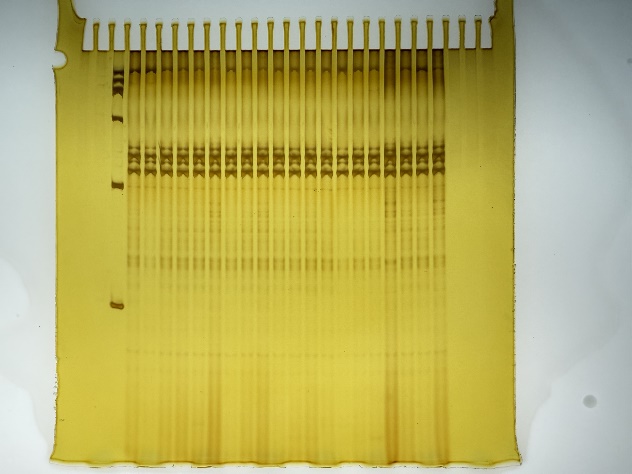**70 |
| BLF-**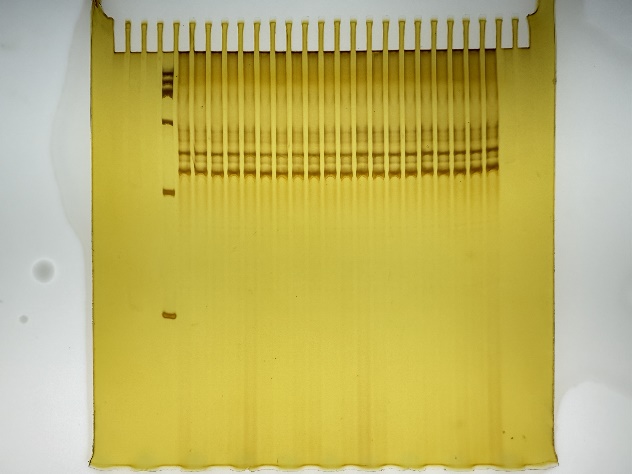**71 | BLF-**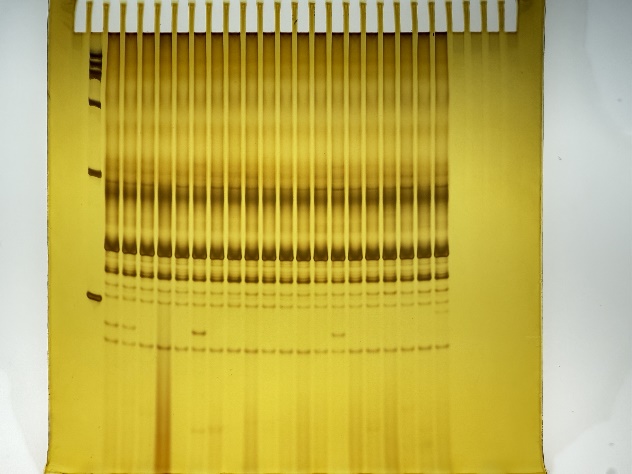**72 |
| BLF-**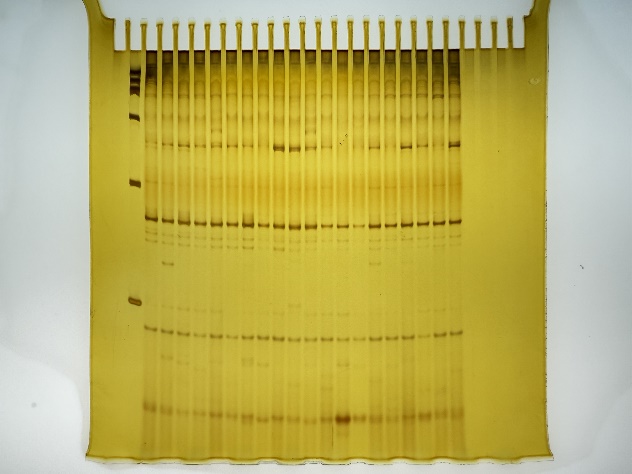**73 | BLF-**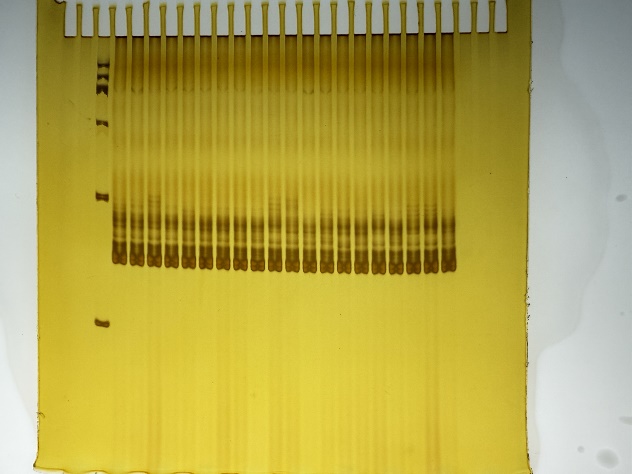**74 |
| BLF-**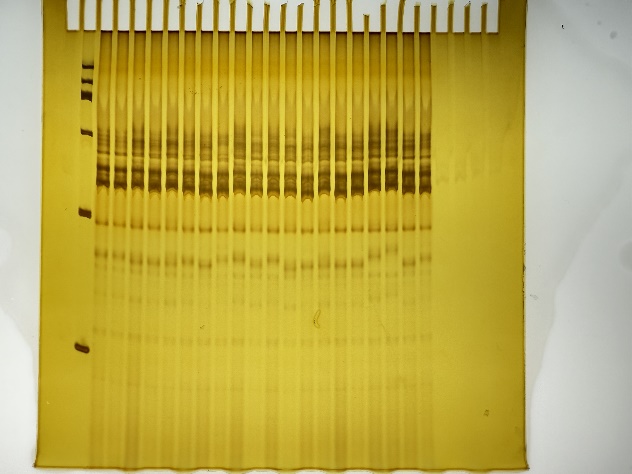**75 | BLF-**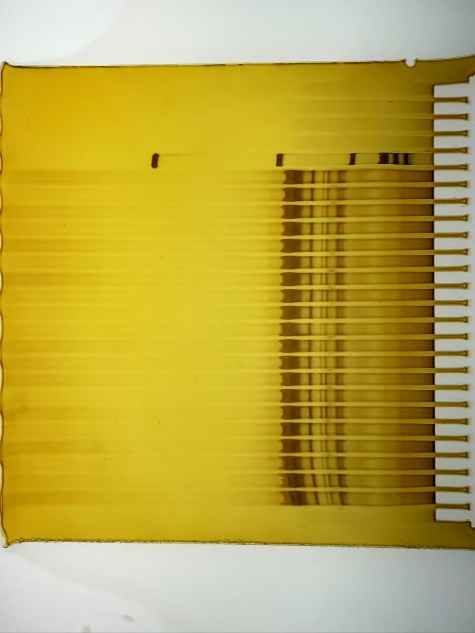**76 |
| BLF-**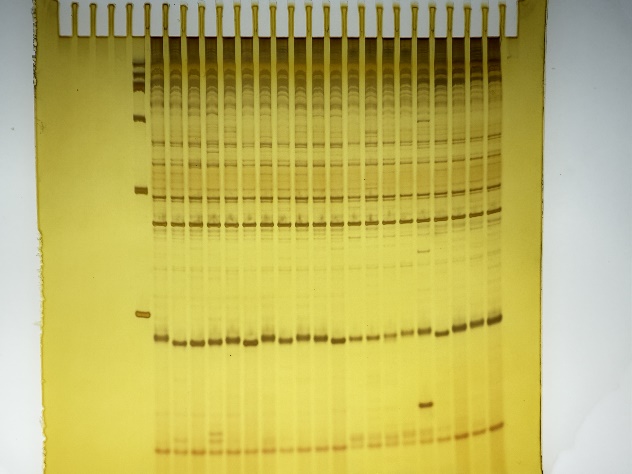**77 | BLF-**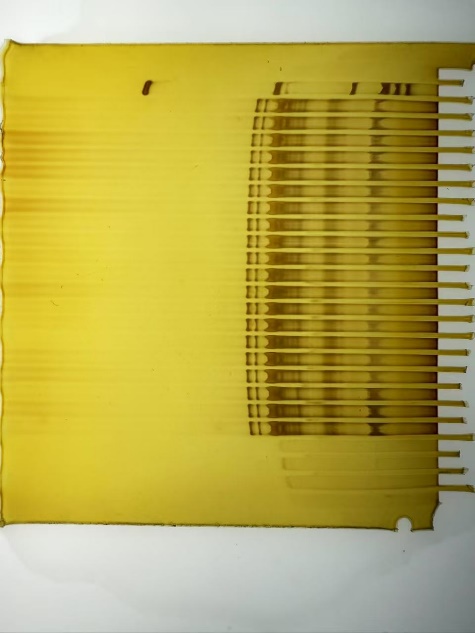**78 |
| BLF-**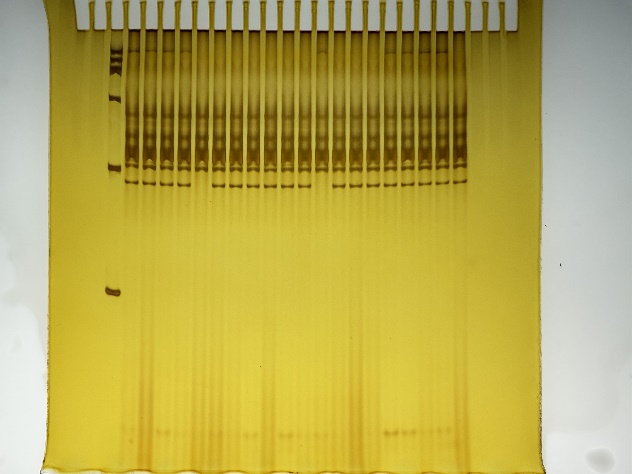**79 | BLF-**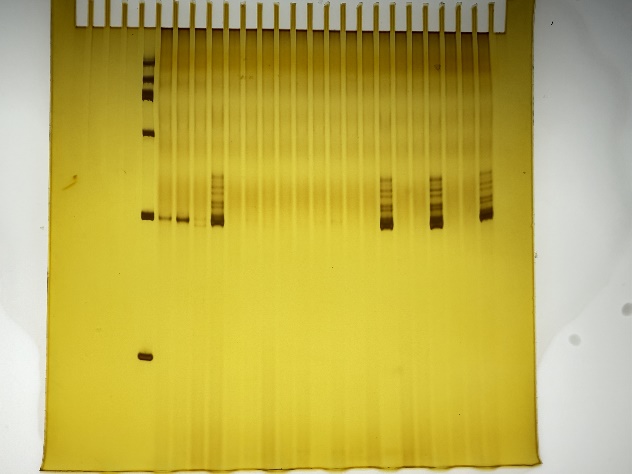**80 |
