# Supplementary material for: Genetic diversity analysis of proso millet (Panicum miliaceum L.) germplasm resources based on phenotypic traits and SSR markers
Source: Front Plant Sci. 2025 Sep 8;16:1649200. doi: 10.3389/fpls.2025.1649200 (PMC12450881; doi:10.3389/fpls.2025.1649200)
Supplement: Supplementary file 3 [file Table3.docx]

**Figure S3 Amplification bands of 15 polymorphic SSR markers.**

| 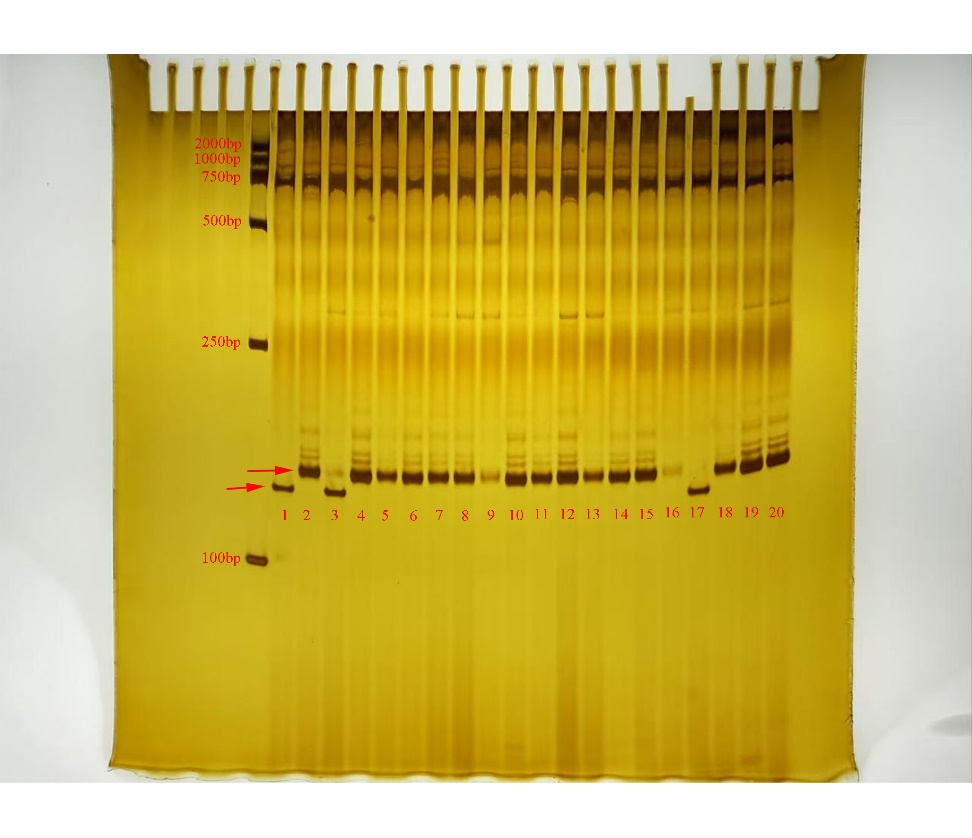BLF-41 | 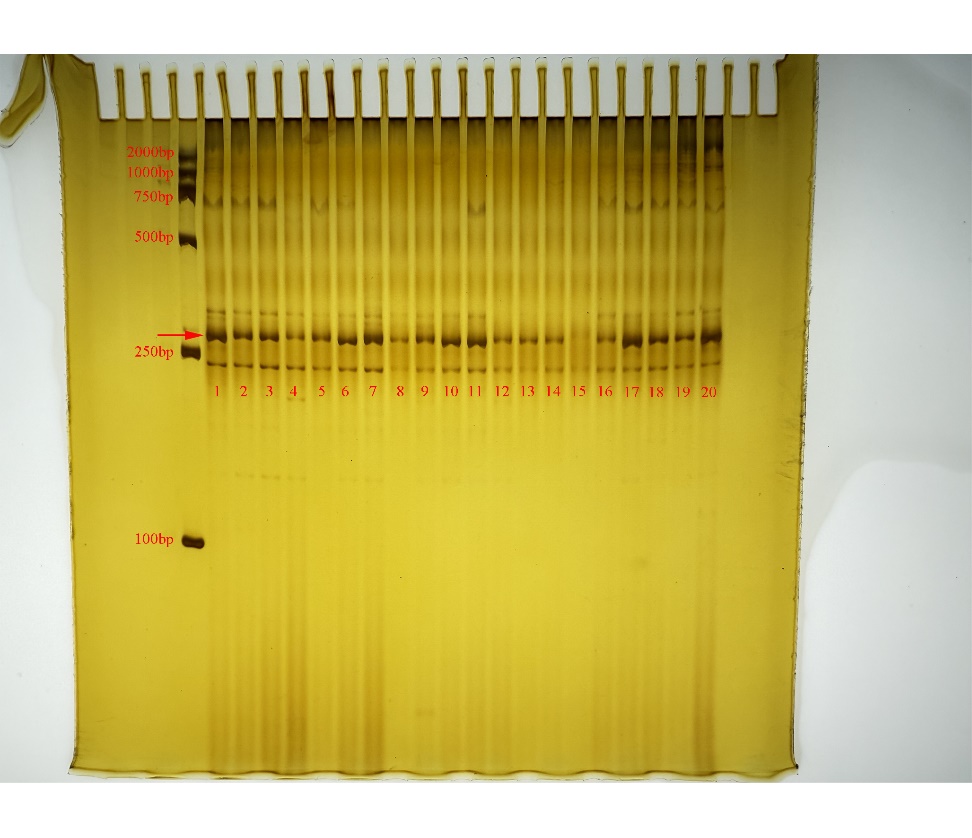BLF-4 |
| --- | --- |
| 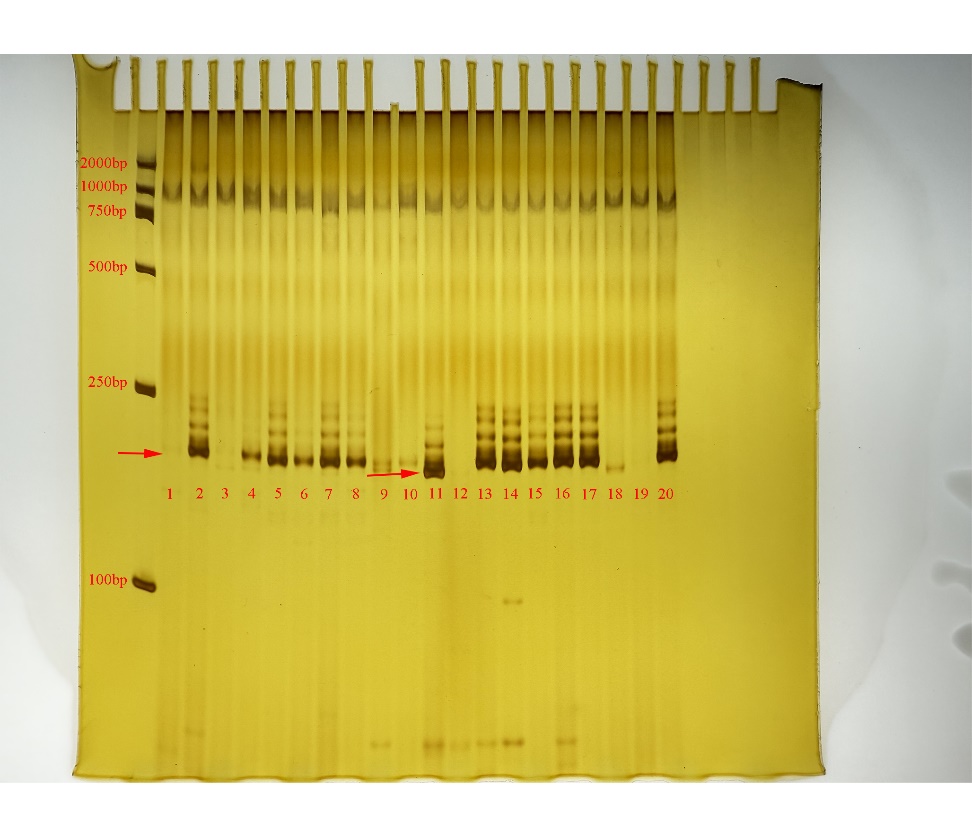BLF-47 | 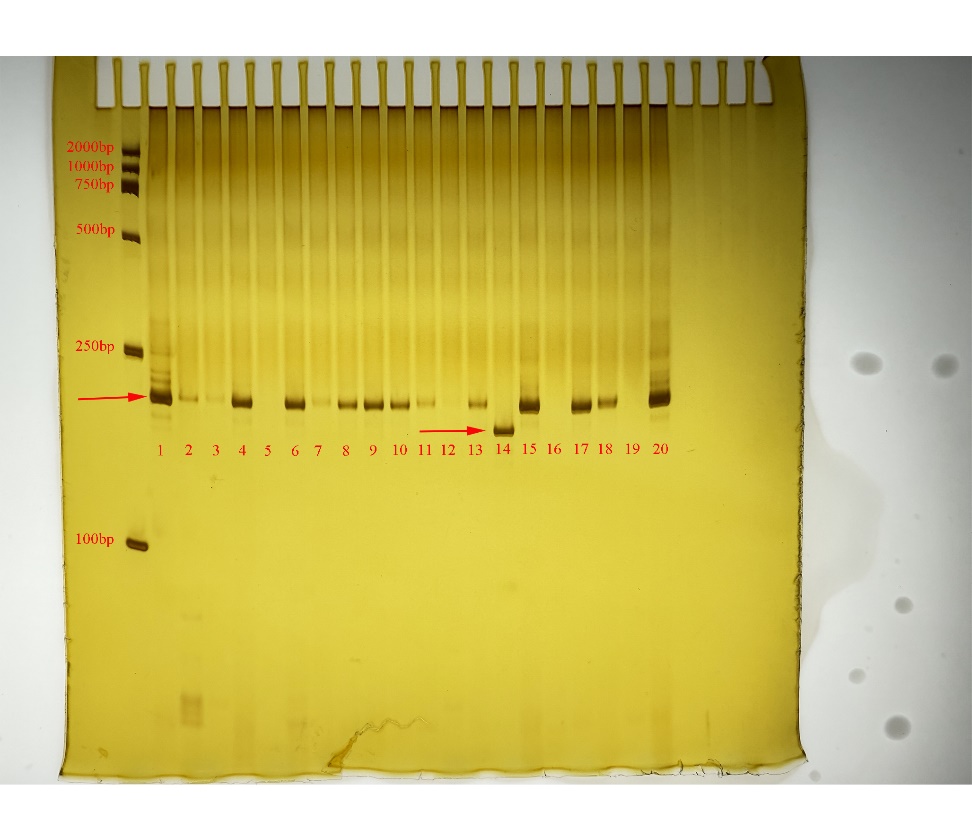BLF-8 |
| 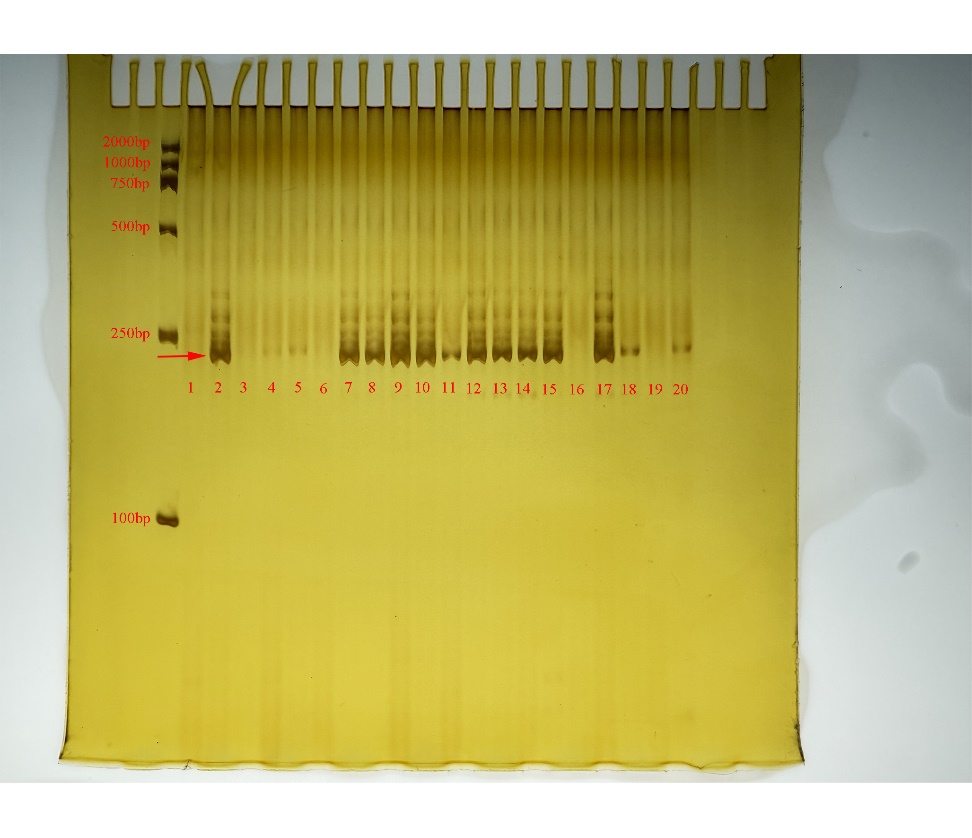BLF-9 | 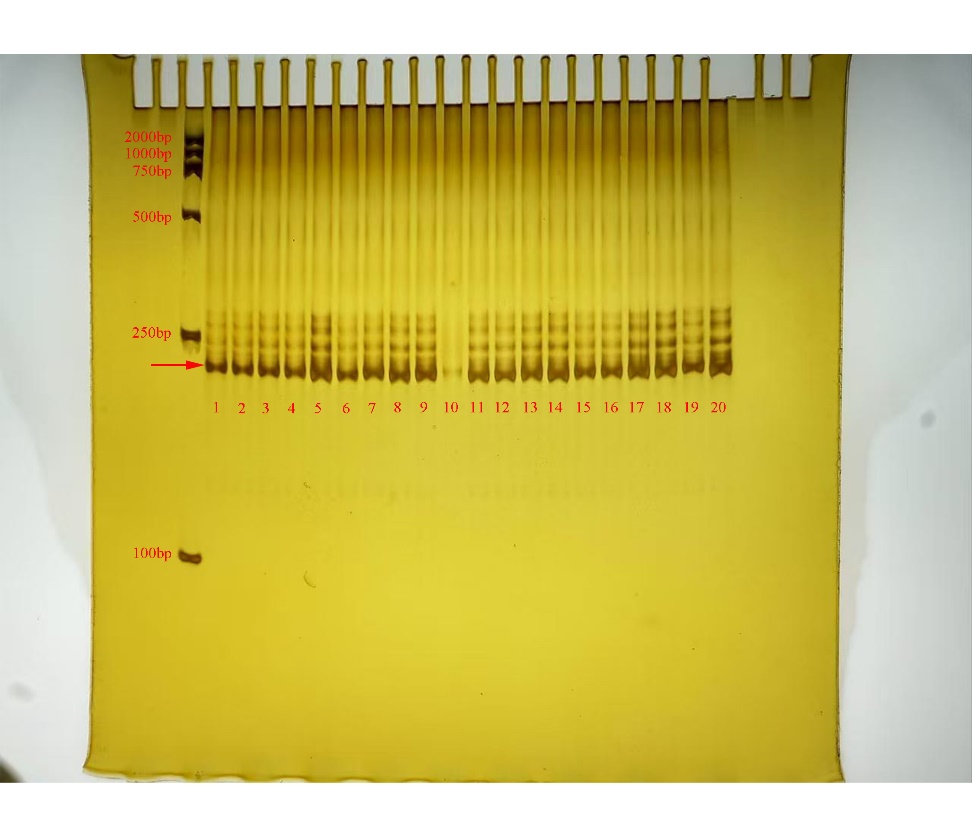BLF-51 |
| 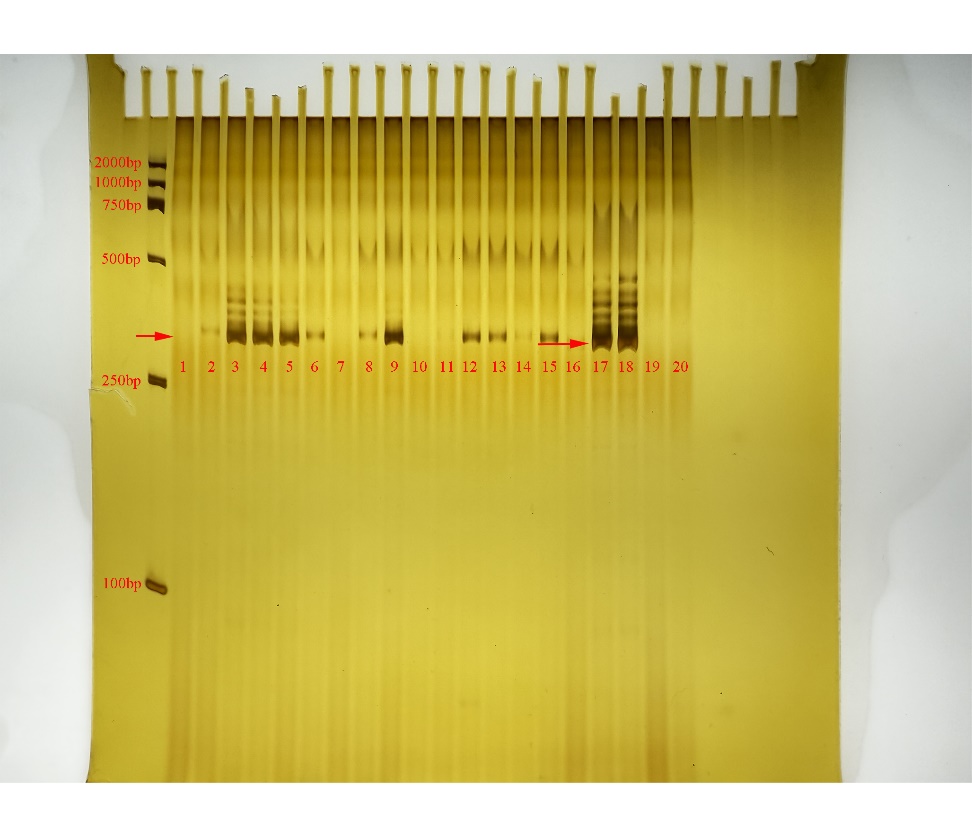BLF-52 | 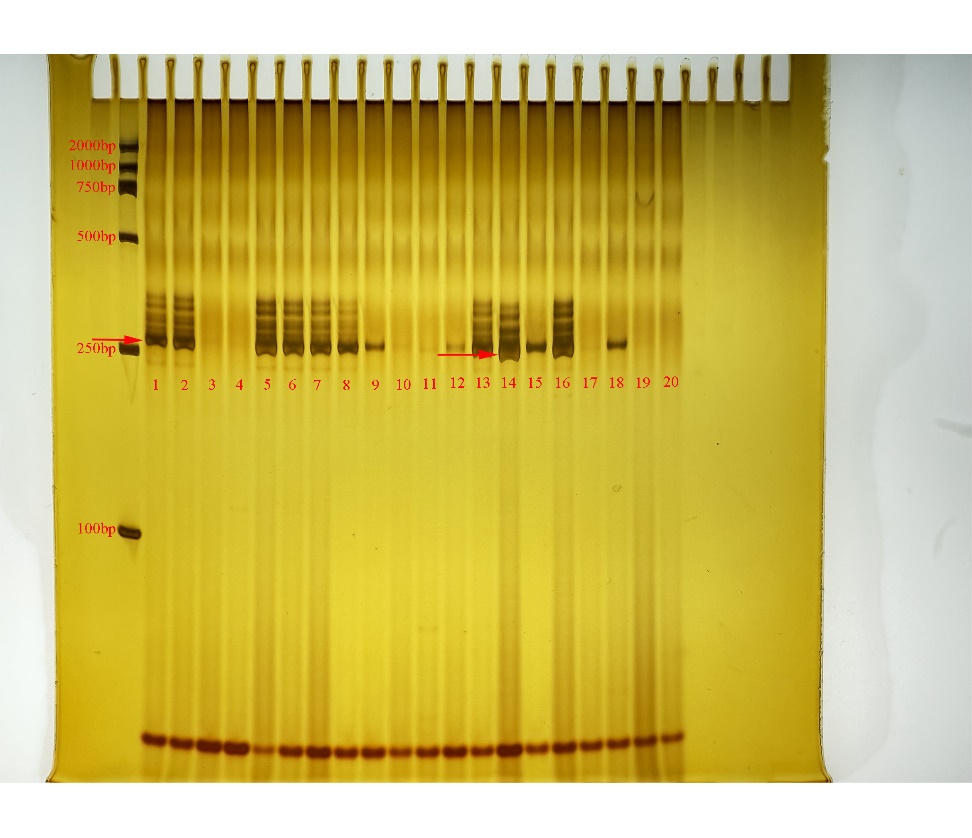BLF-58 |
| 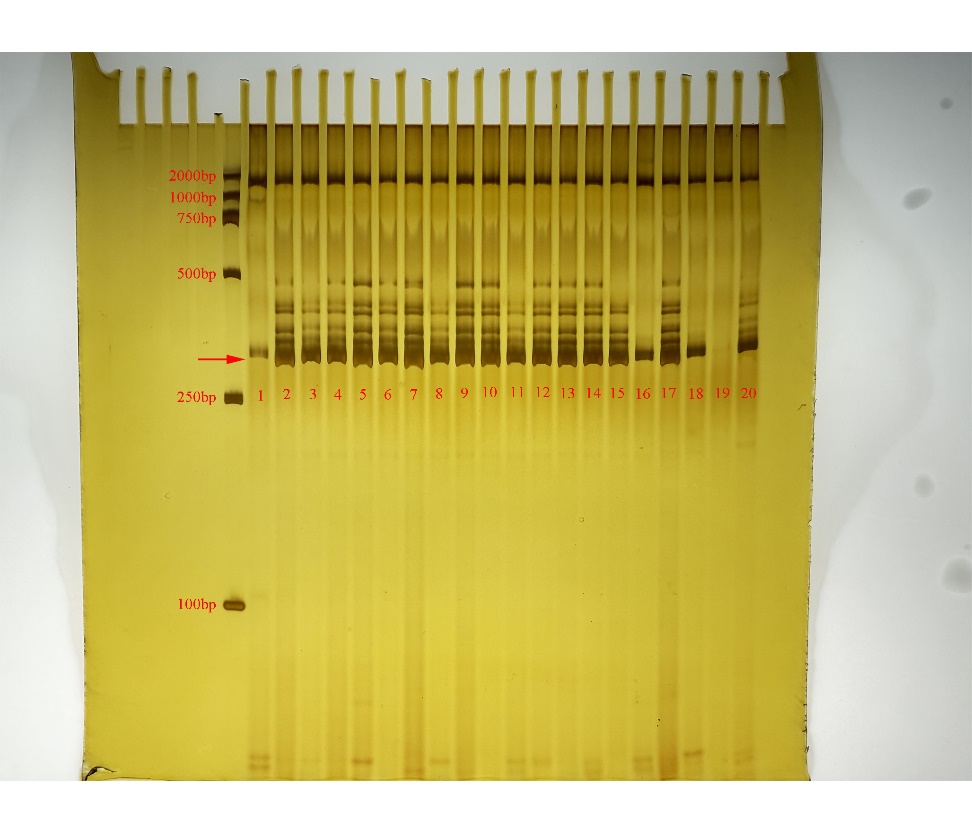BLF-59 | 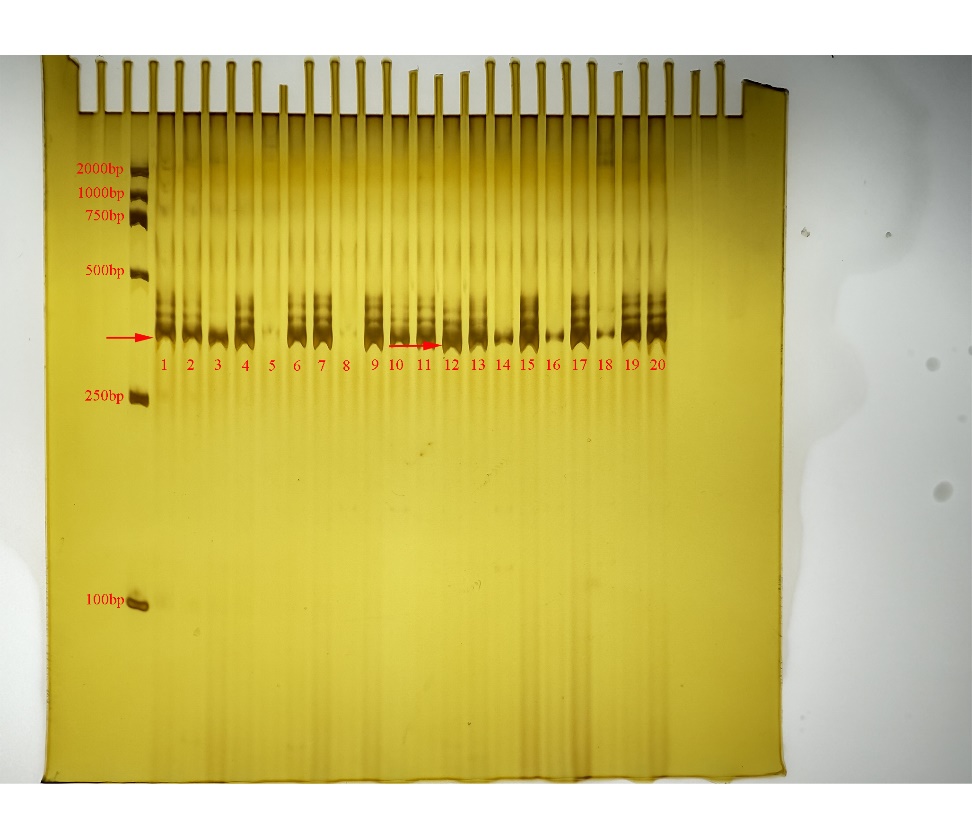BLF-61 |
| 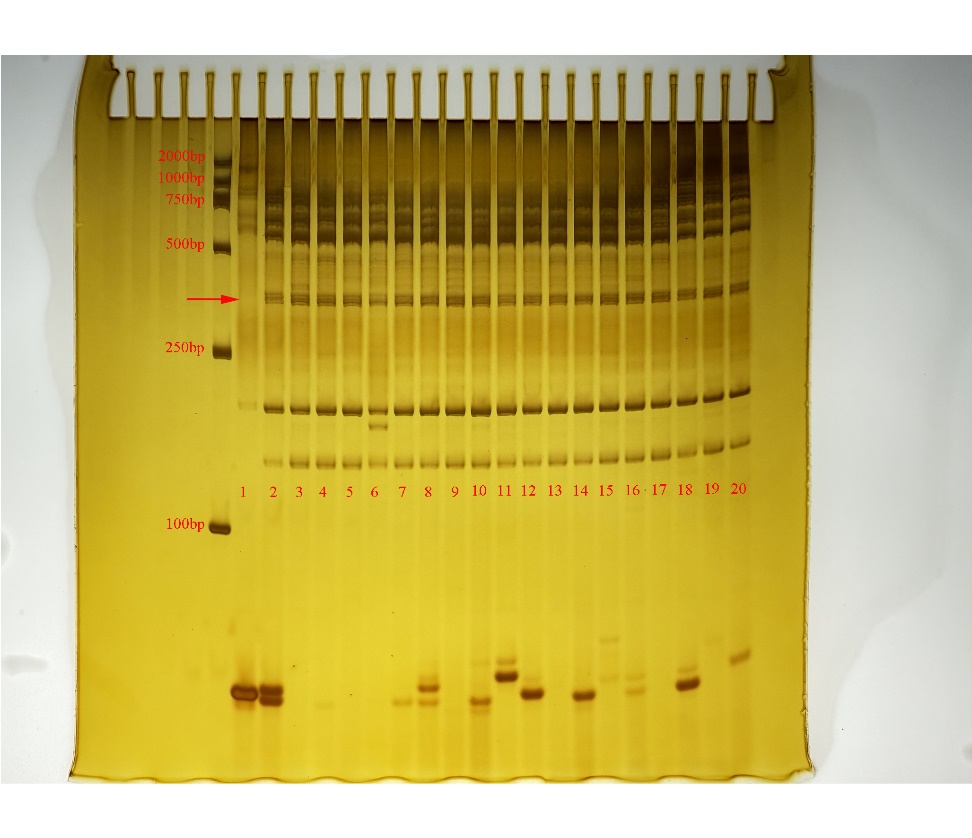BLF-21 | 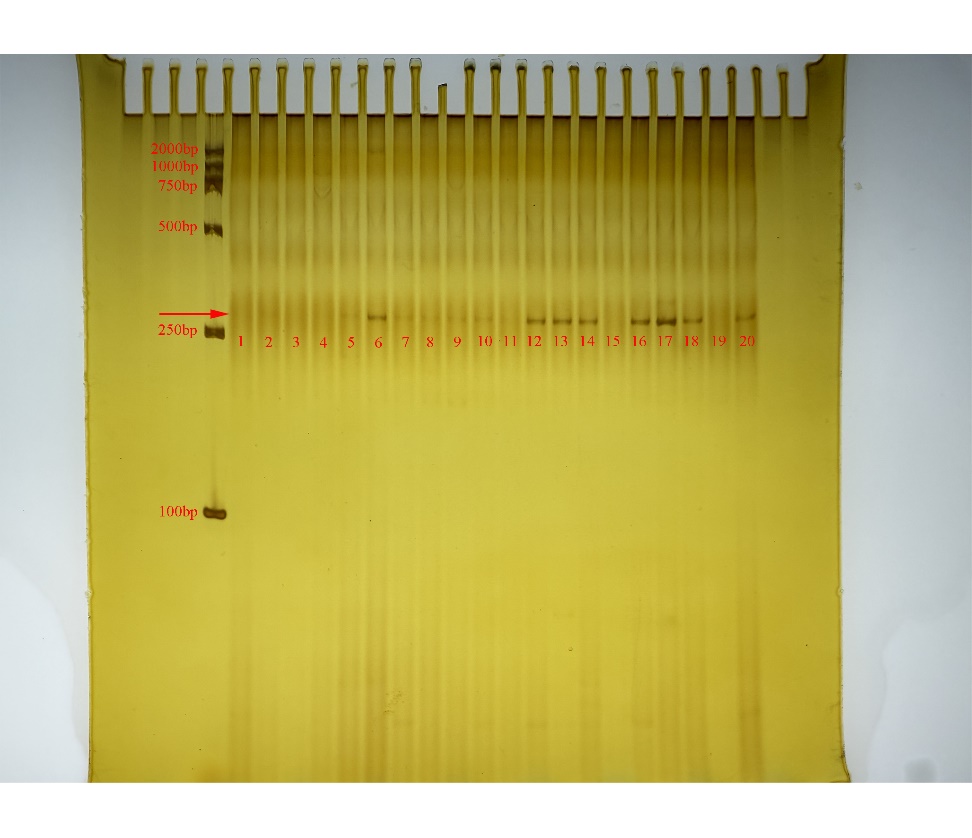BLF-66 |
| 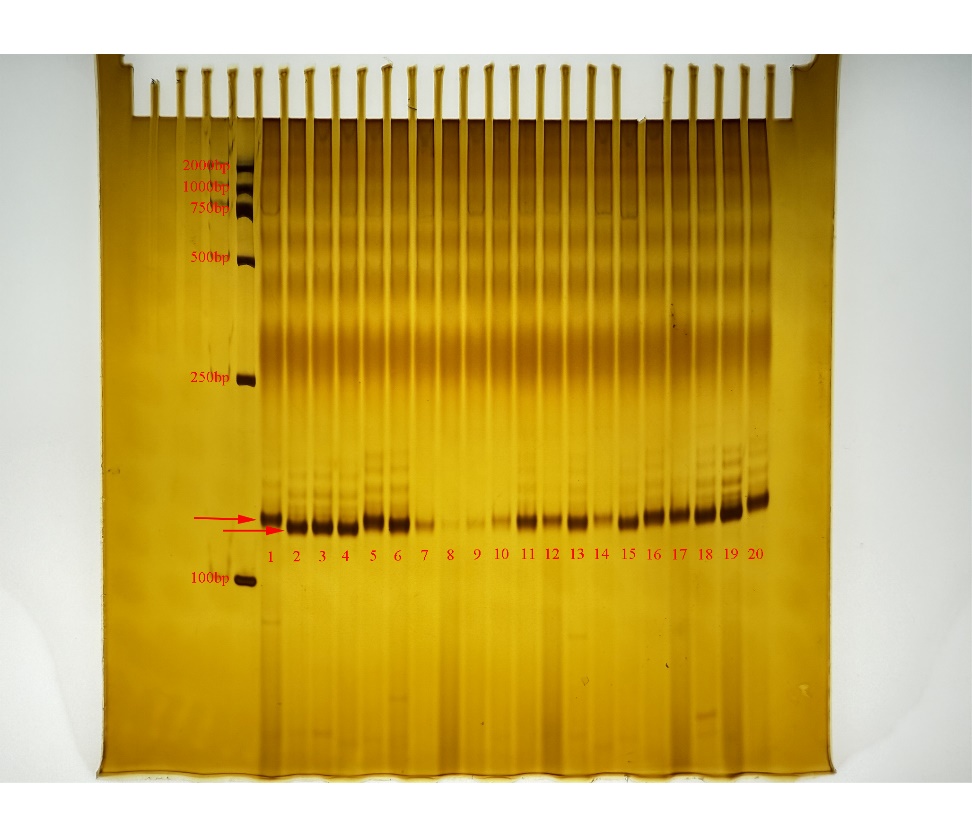BLF-27 | 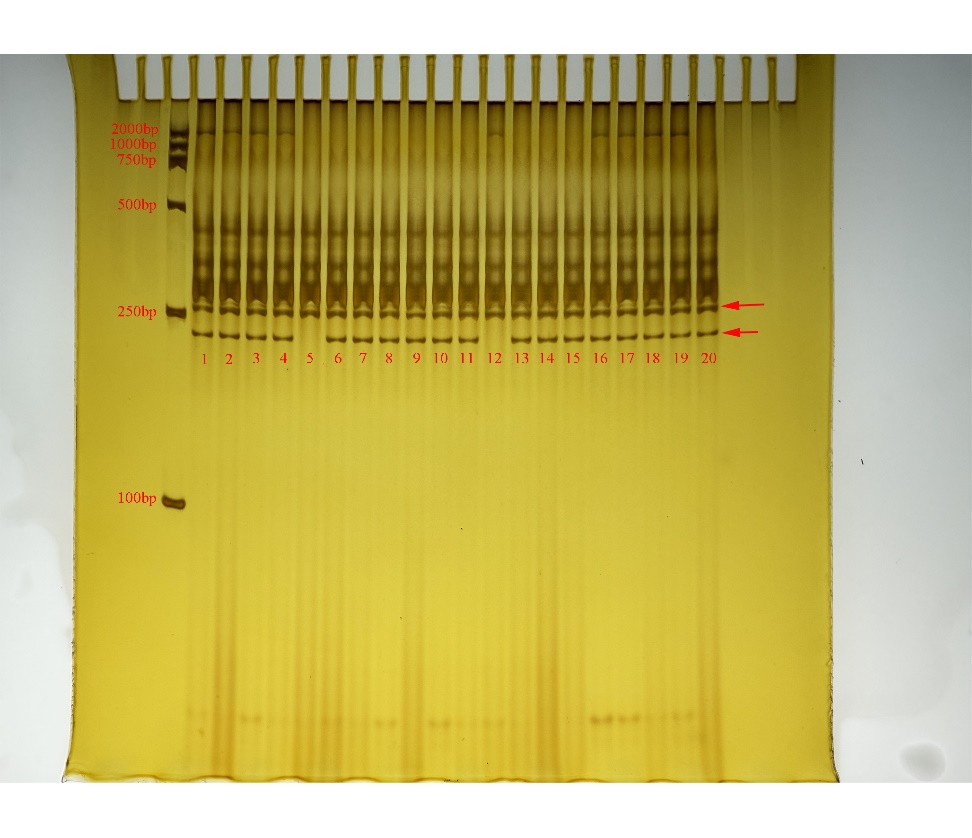BLF-79 |
| 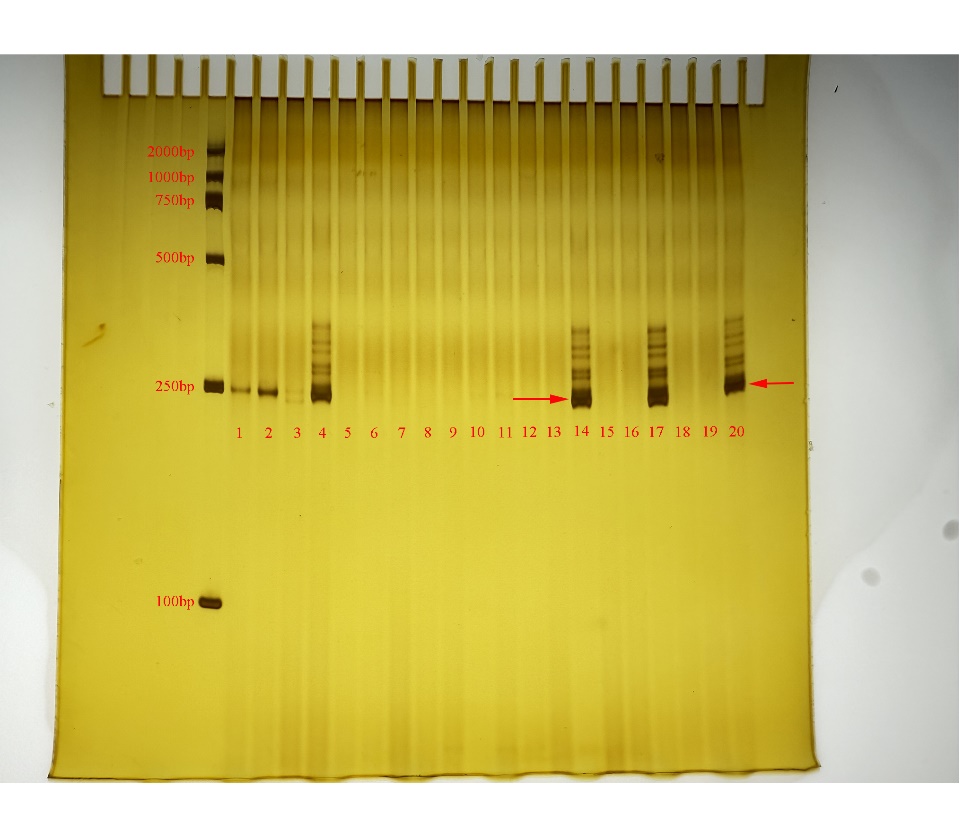BLF-80 |  |
| 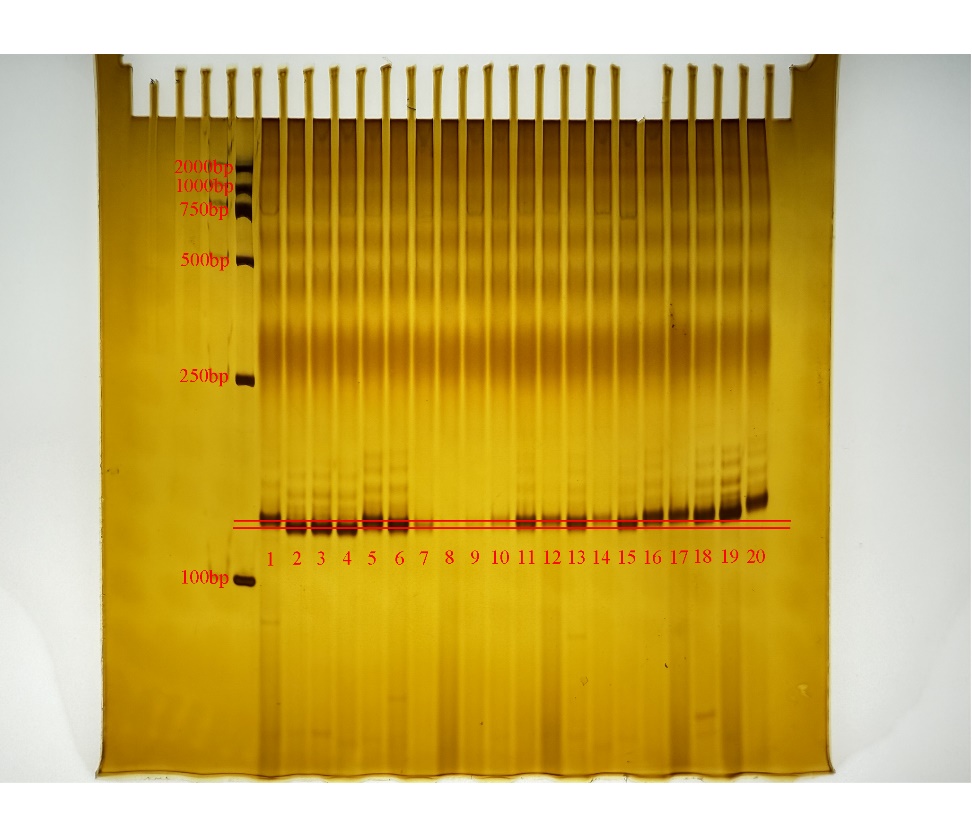BLF-27 | 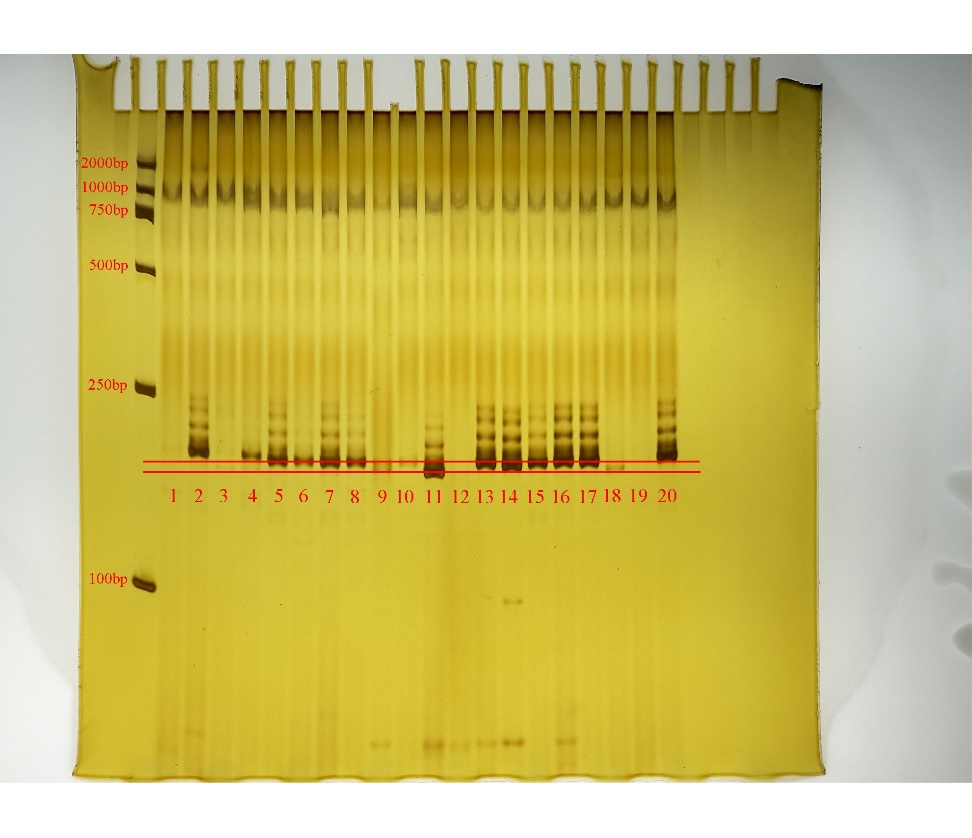BLF-47 |
