# Supplementary material for: Genetic diversity analysis of proso millet (Panicum miliaceum L.) germplasm resources based on phenotypic traits and SSR markers
Source: Front Plant Sci. 2025 Sep 8;16:1649200. doi: 10.3389/fpls.2025.1649200 (PMC12450881; doi:10.3389/fpls.2025.1649200)
Supplement: Supplementary file 4 [file Table4.docx]

**Figure S4 Amplification bands of 15 polymorphic SSR markers across 147 samples.**

| 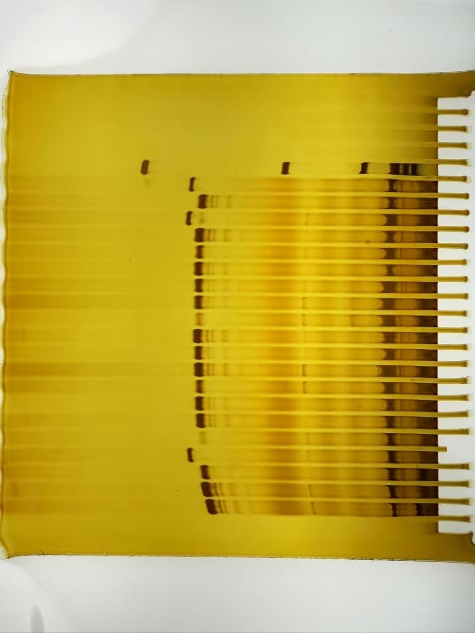BLF-41 1-20 | BLF-41 21-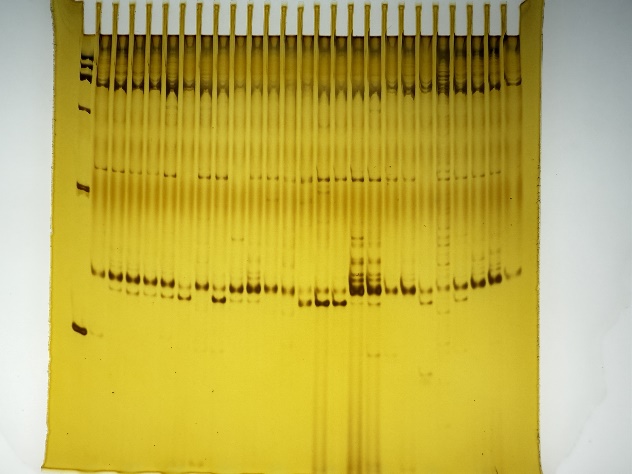45 |
| --- | --- |
| BLF-41 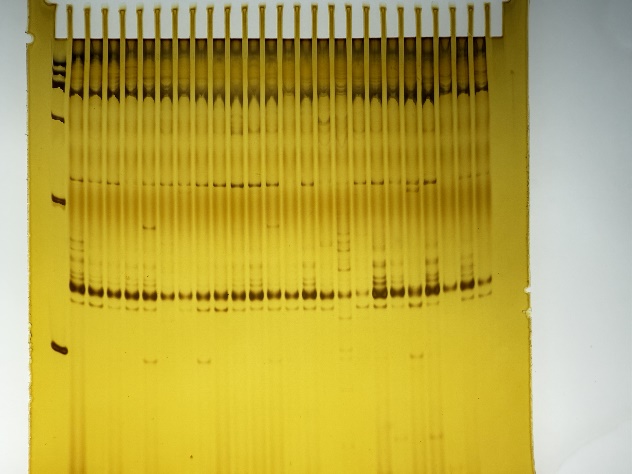46-69 | BLF-41 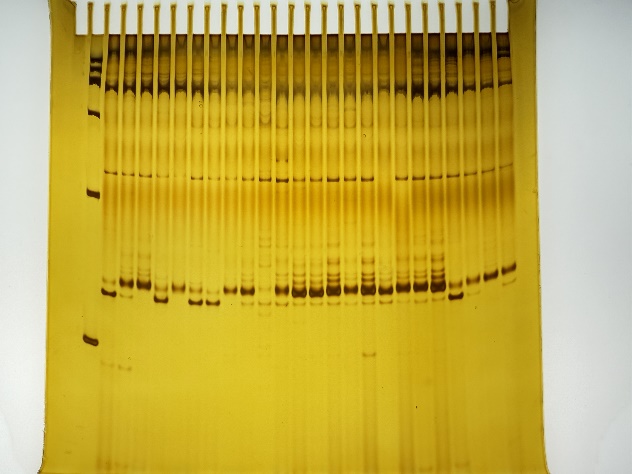70-93 |
| BLF-41 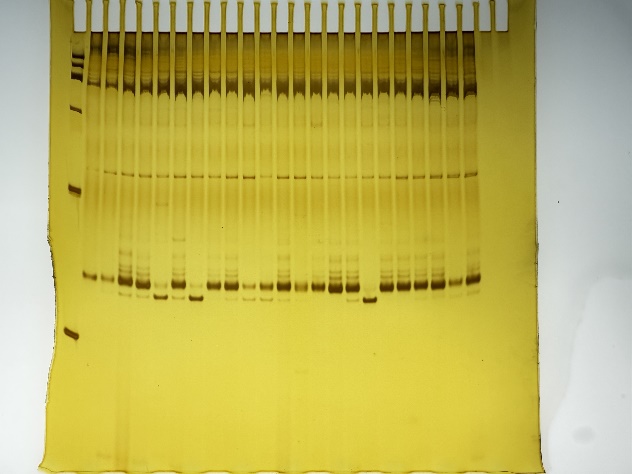94-116 | BLF-41 117-134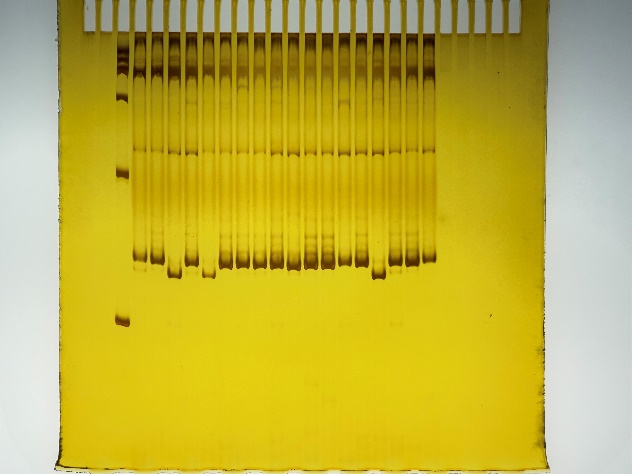 |
| BLF-41 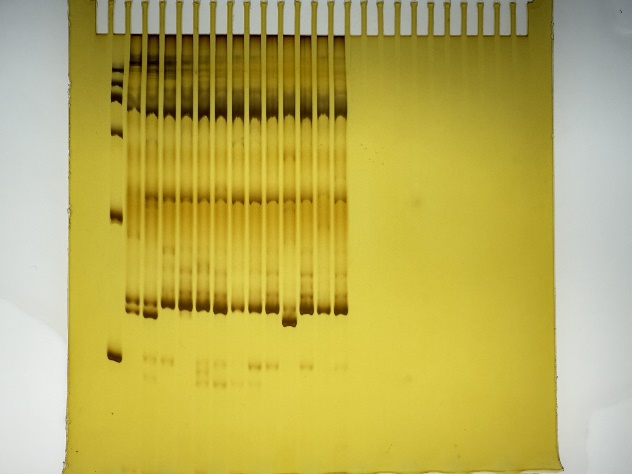135-147 |  |
| 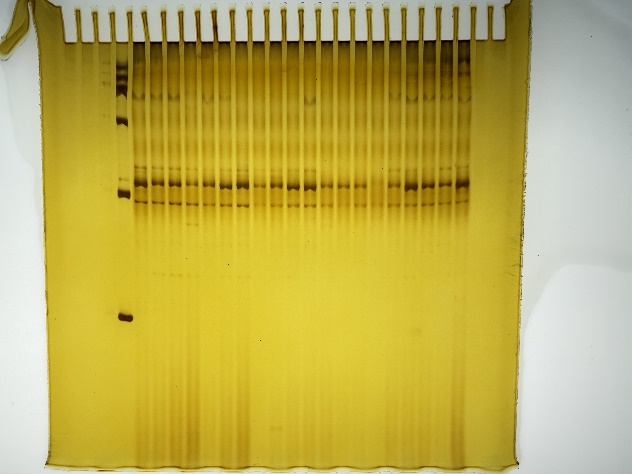BLF-4 1-20 | BLF-4 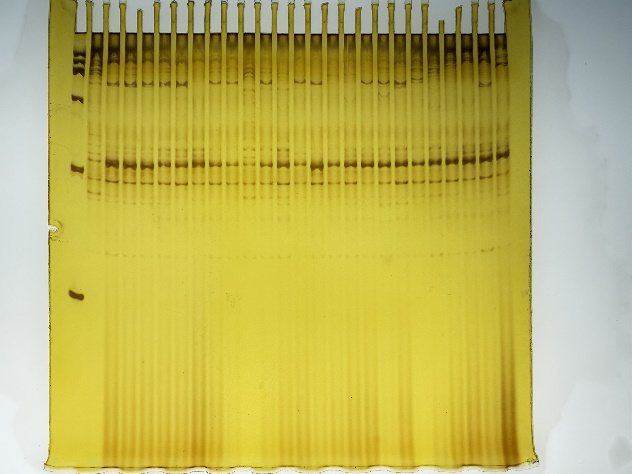21-45 |
| BLF-4 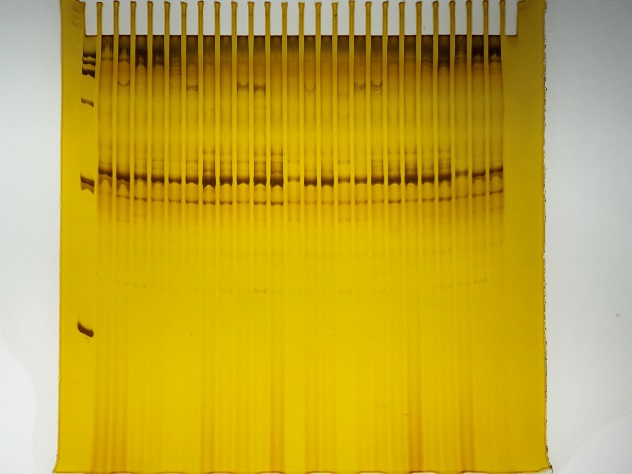46-69 | BLF-4 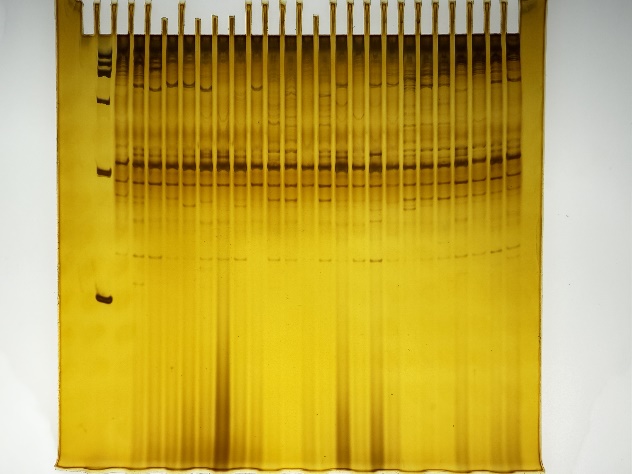70-93 |
| BLF-4 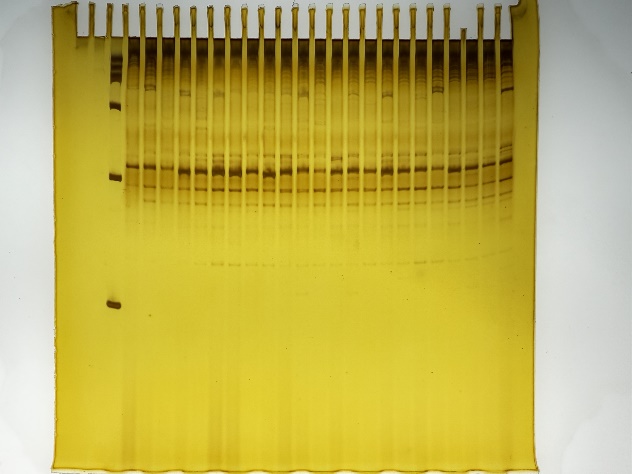94-116 | BLF-4 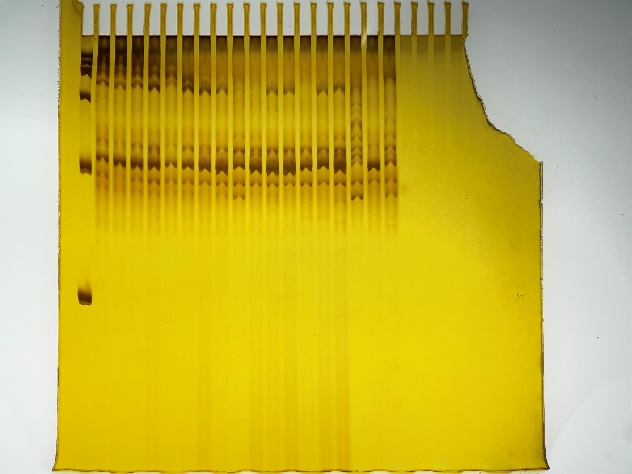117-134 |
| BLF-4 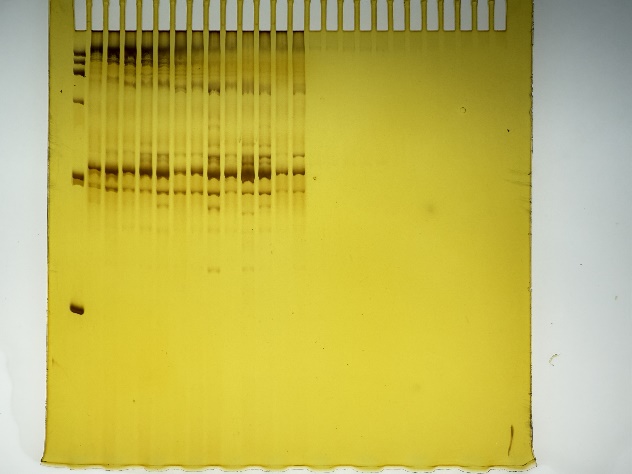135-147 |  |
| BLF-4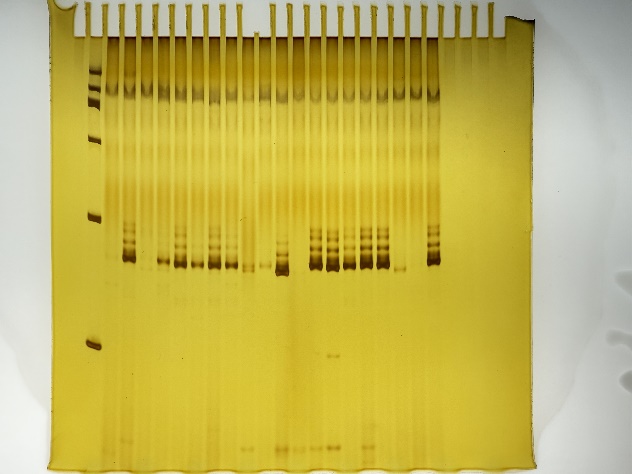7 1-20 | 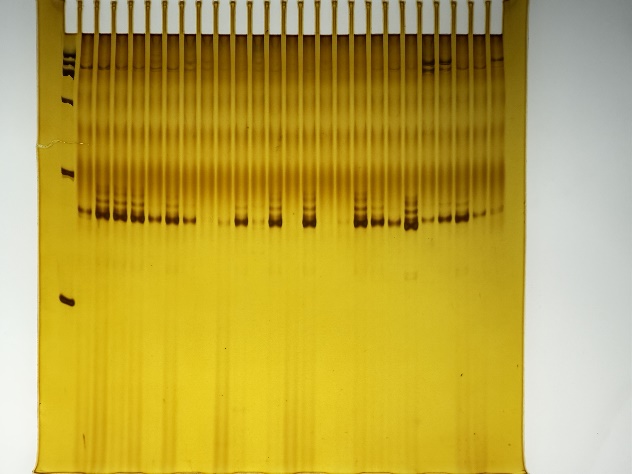BLF-47 21-45 |
| BLF-47 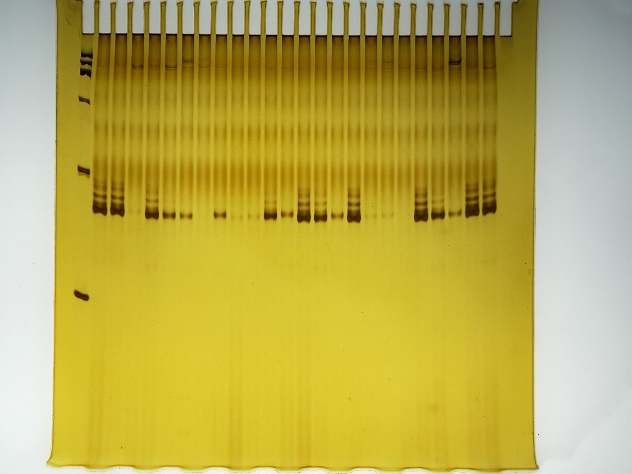46-69 | BLF-47 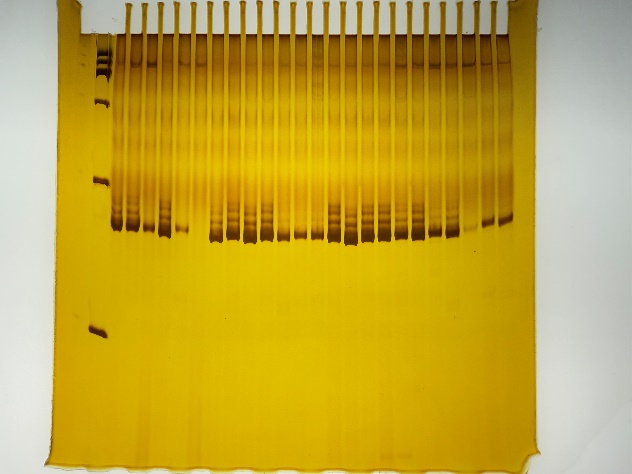70-93 |
| BLF-47 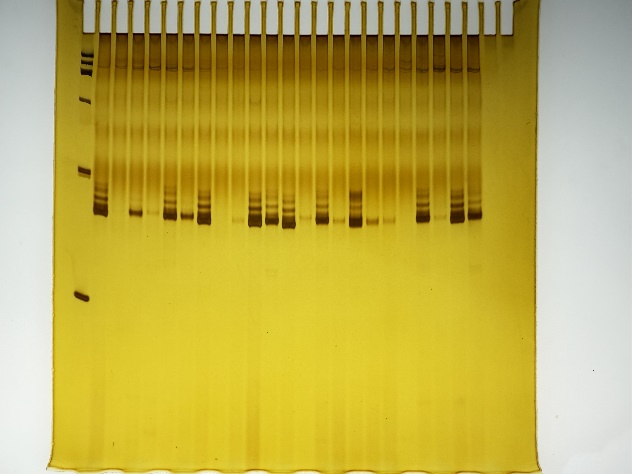94-116 | BLF-47 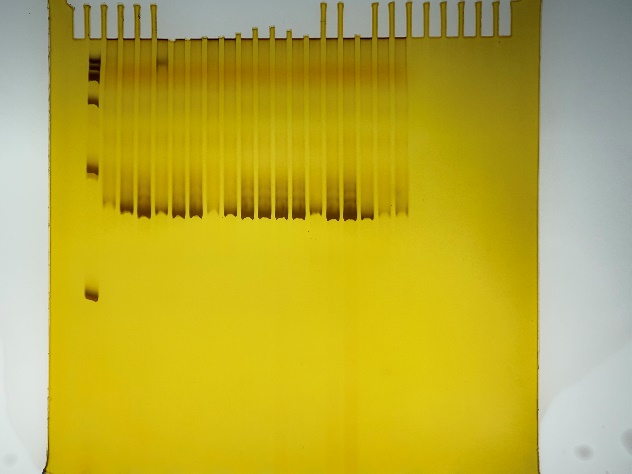117-134 |
| BLF-47 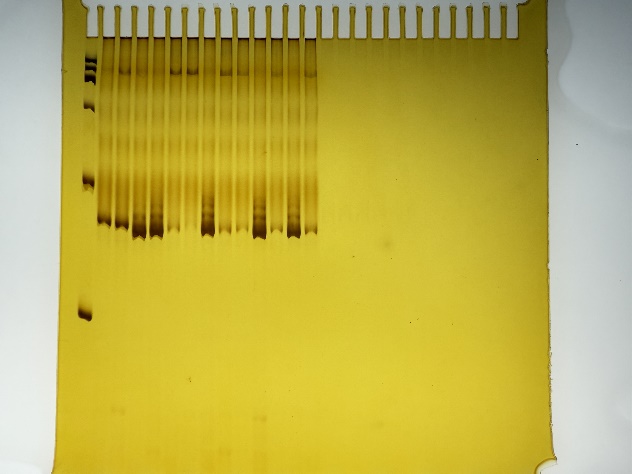135-147 |  |
| 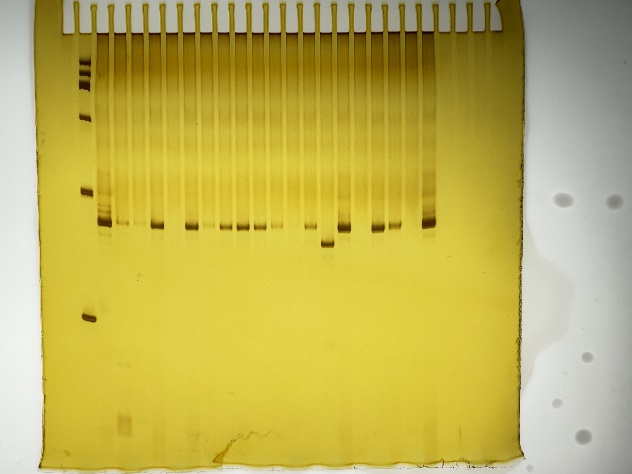BLF-8 1-20 | BLF-8 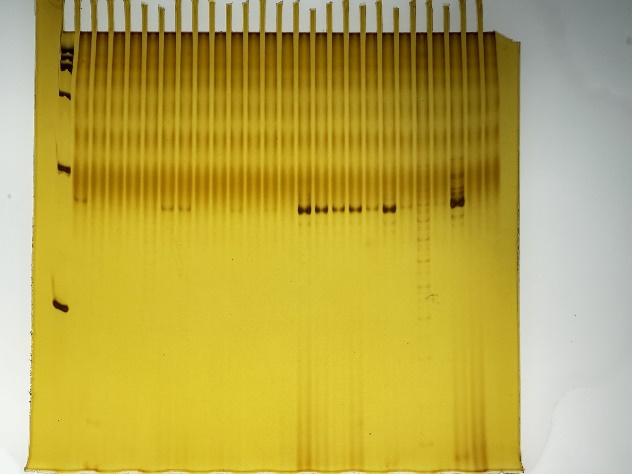21-45 |
| BLF-8 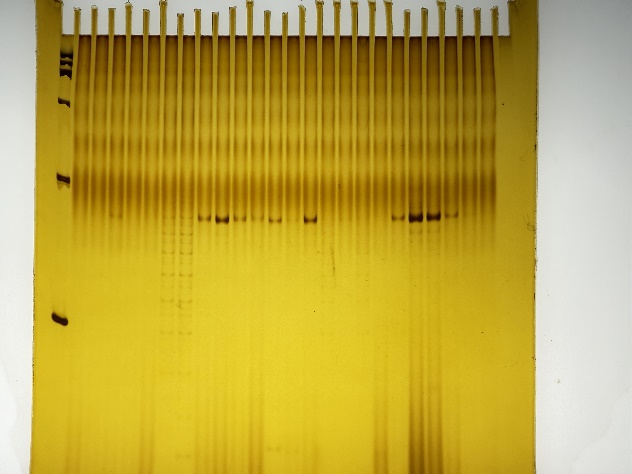46-69 | BLF-8 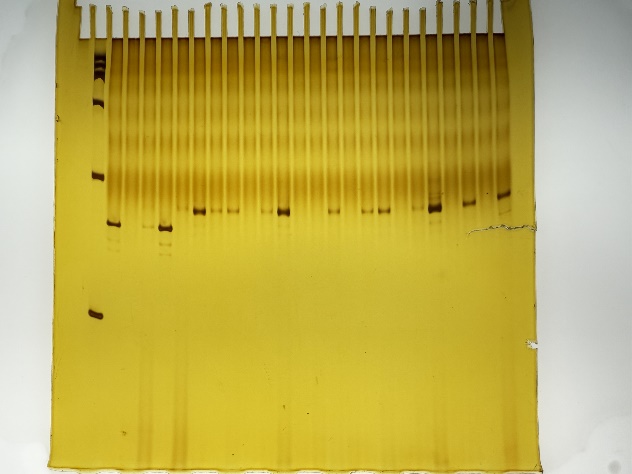70-93 |
| BLF-8 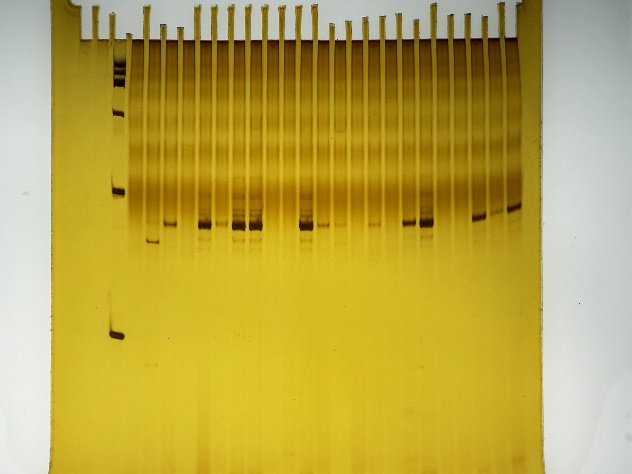94-116 | BLF-8 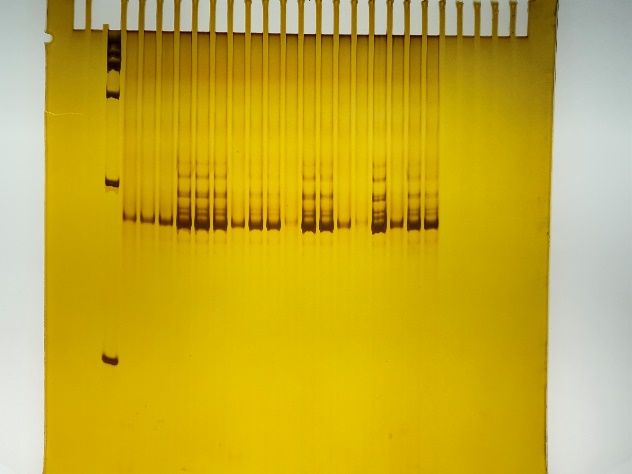117-134 |
| BLF-8 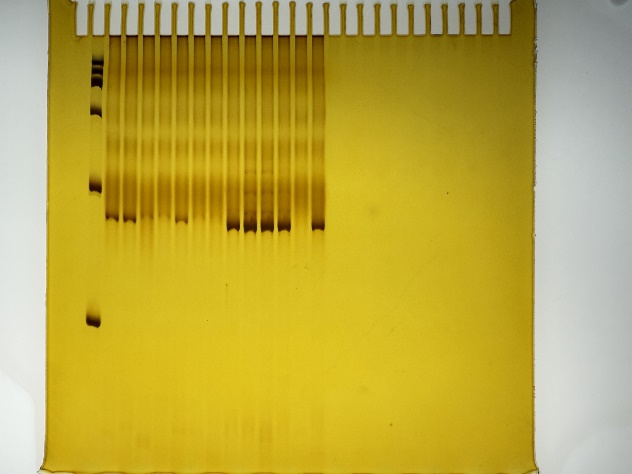135-147 |  |
| BLF-9 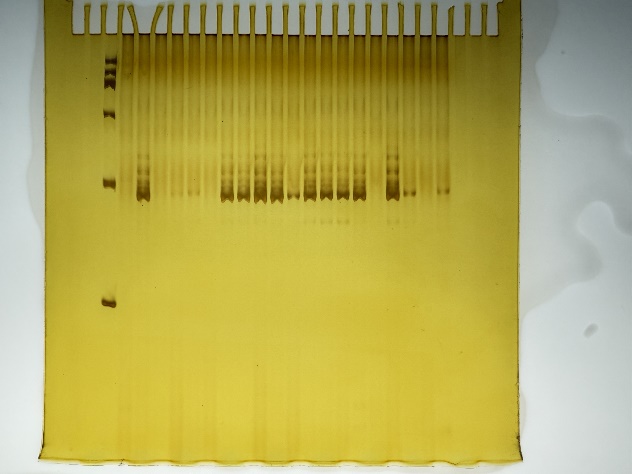1-20 | BLF-9 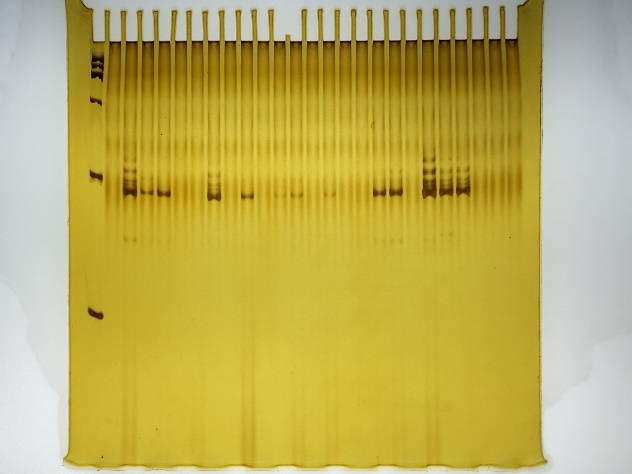21-45 |
| BLF-9 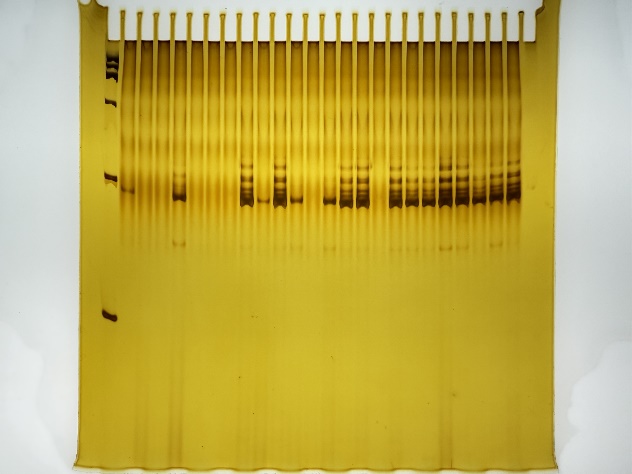46-69 | BLF-9 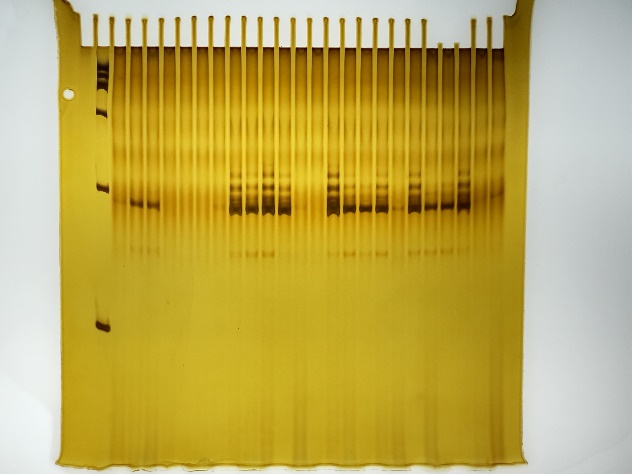70-93 |
| BLF-9 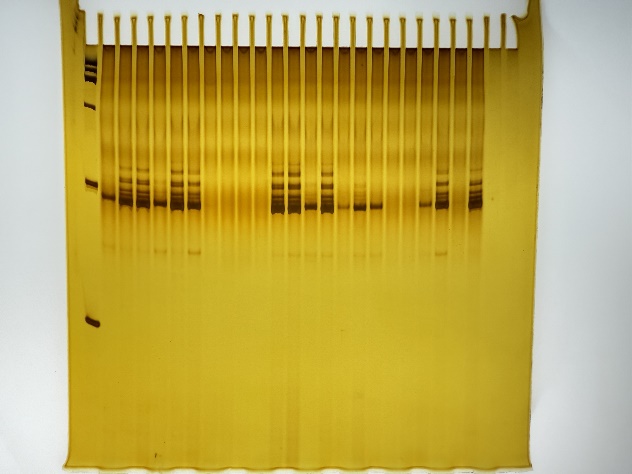94-116 | BLF-9 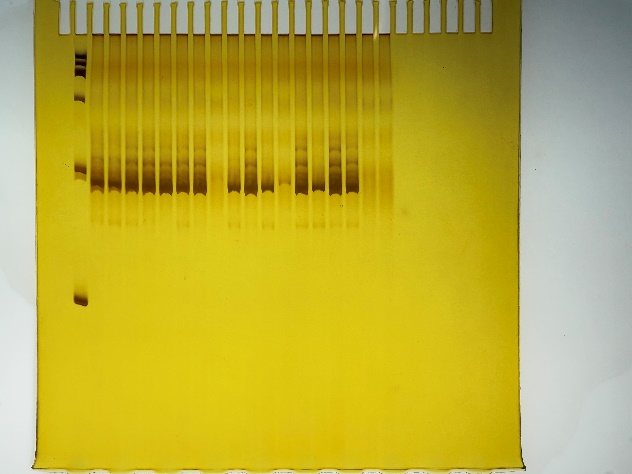117-134 |
| BLF-9 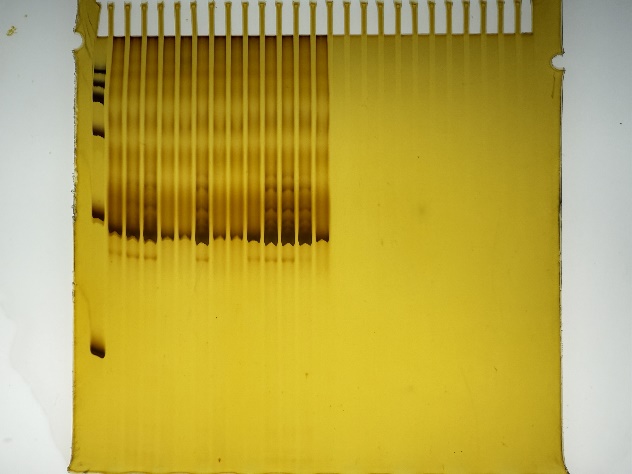135-147 |  |
| 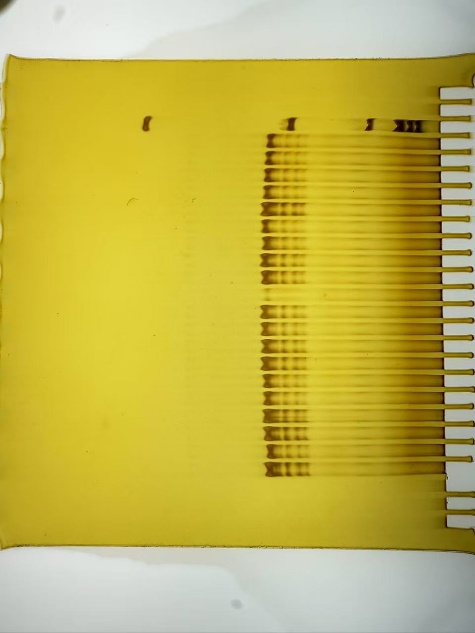BLF-51 1-20 | BLF-51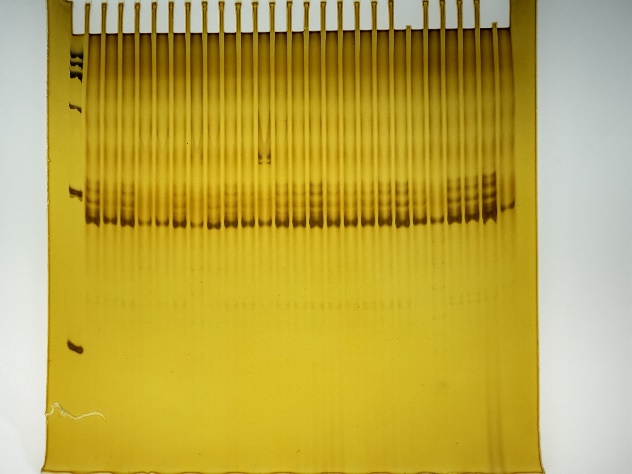 21-45 |
| BLF-51 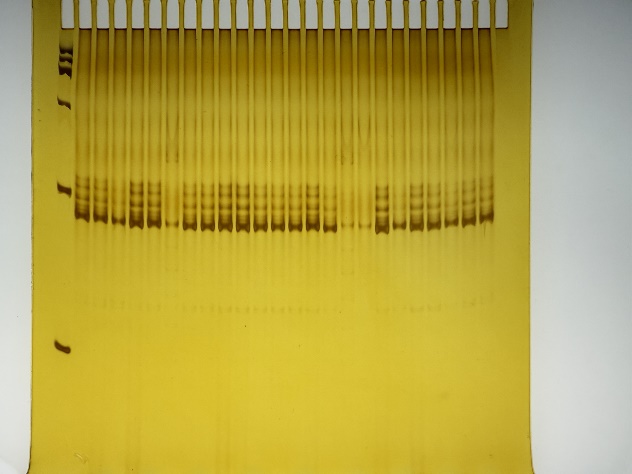46-69 | BLF-51 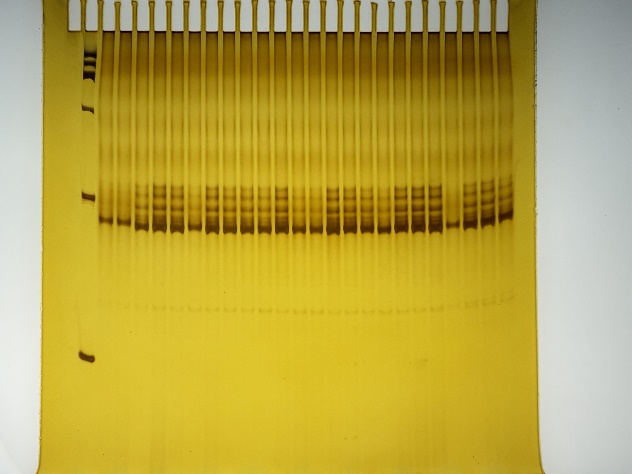70-93 |
| BLF-51 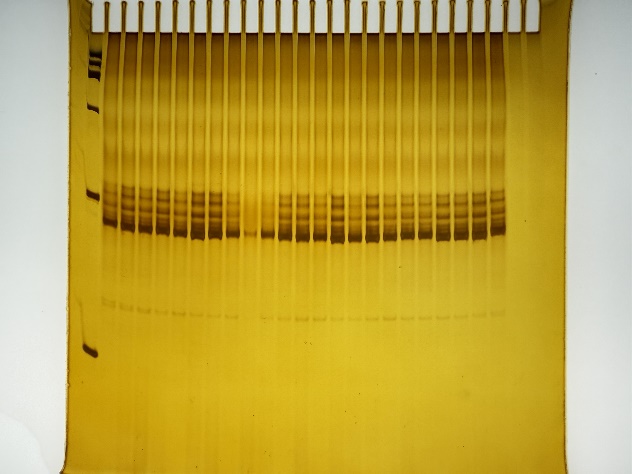94-116 | BLF-51 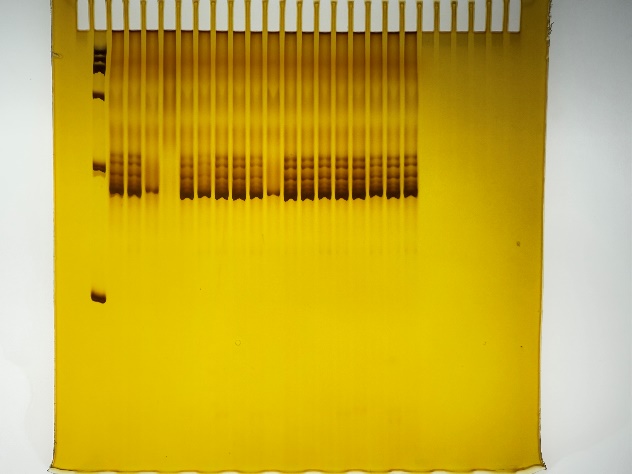117-134 |
| BLF-51 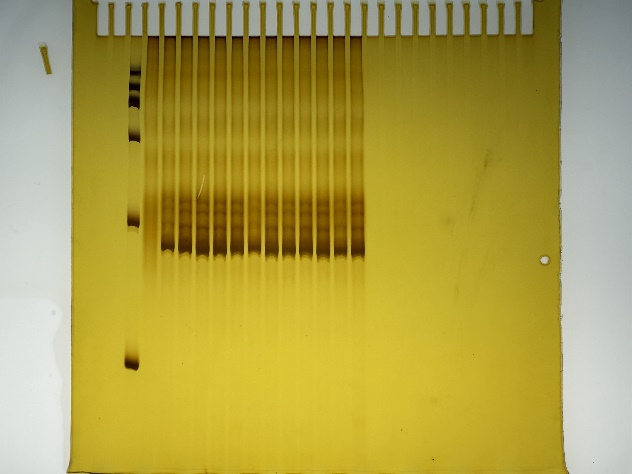135-147 |  |
| 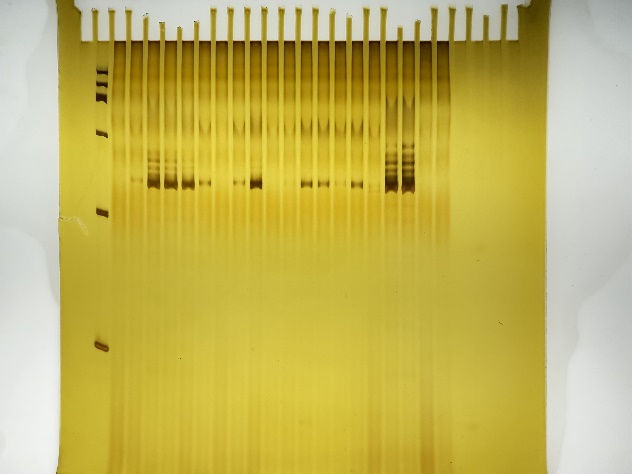BLF-52 1-20 | BLF-52 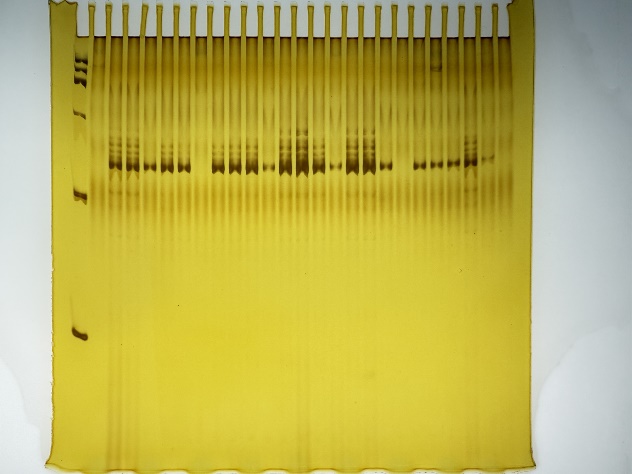21-45 |
| BLF-52 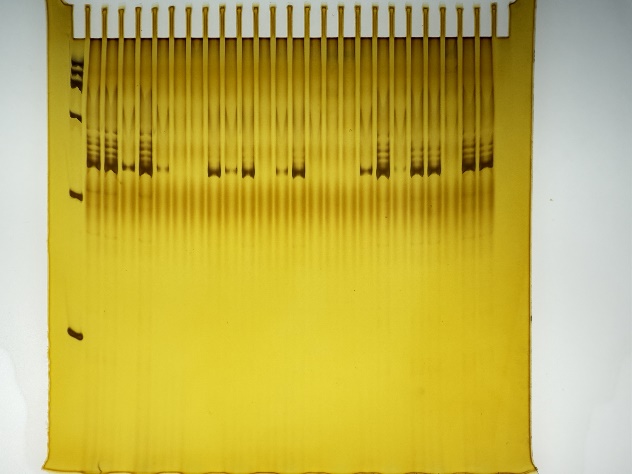46-69 | BLF-52 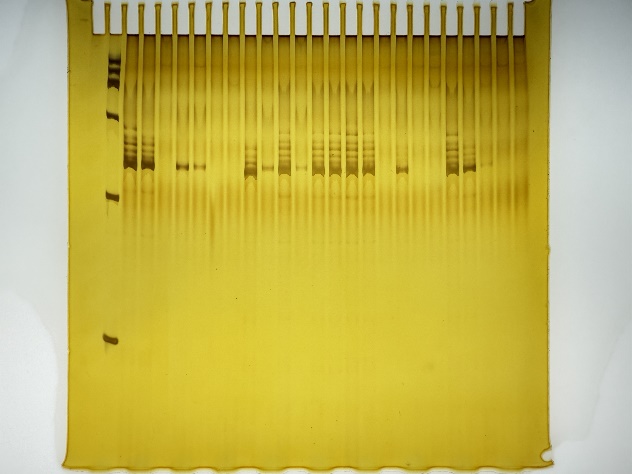70-93 |
| BLF-52 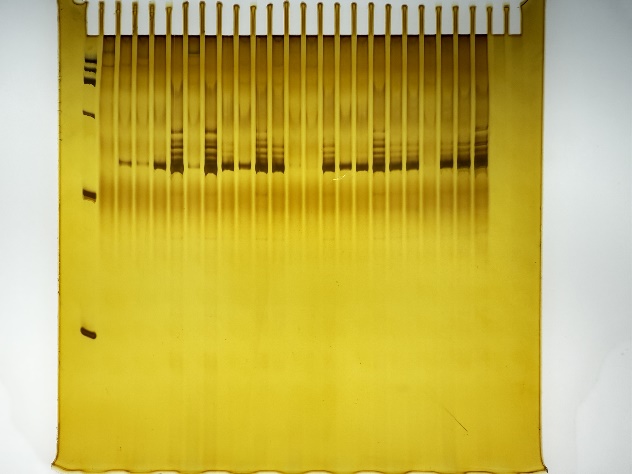94-116 | BLF-52 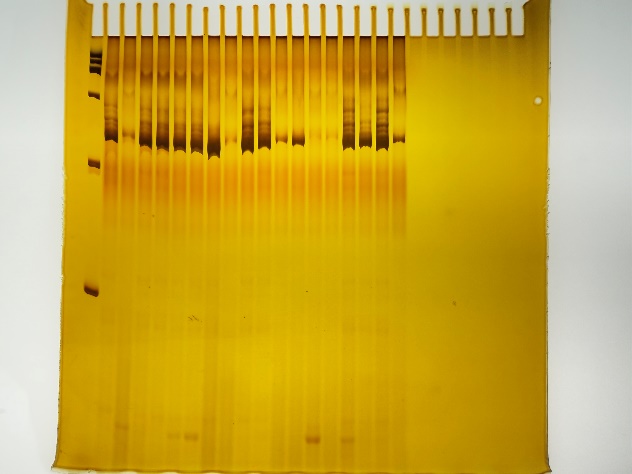117-134 |
| BLF-52 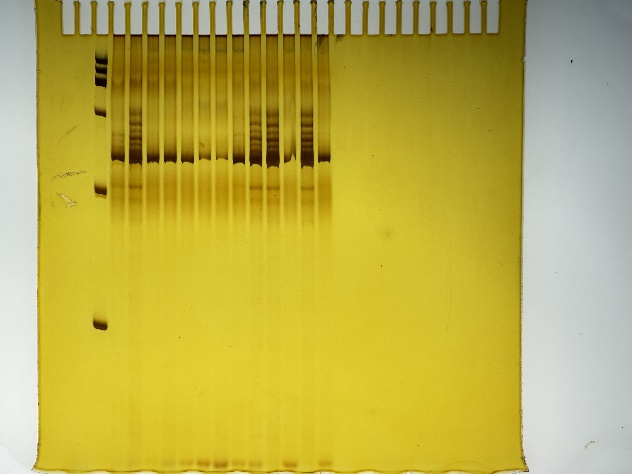135-147 |  |
| BLF-58 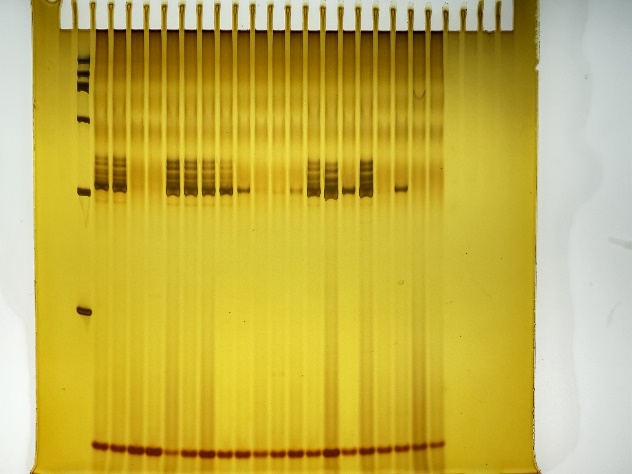1-20 | BLF-58 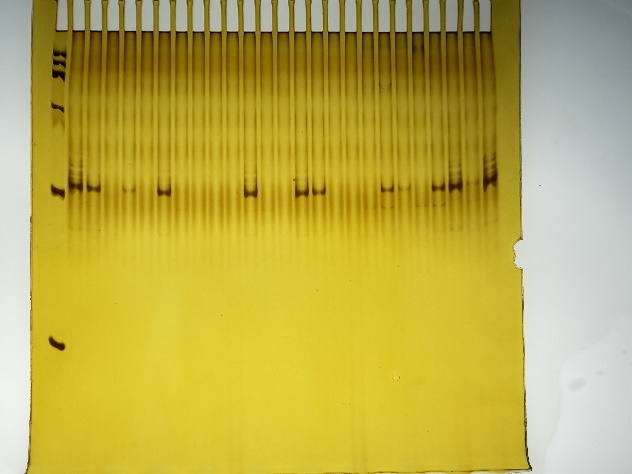21-45 |
| BLF-58 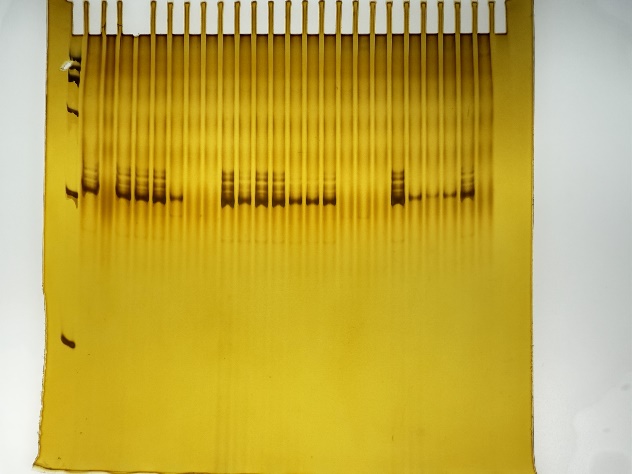46-69 | BLF-58 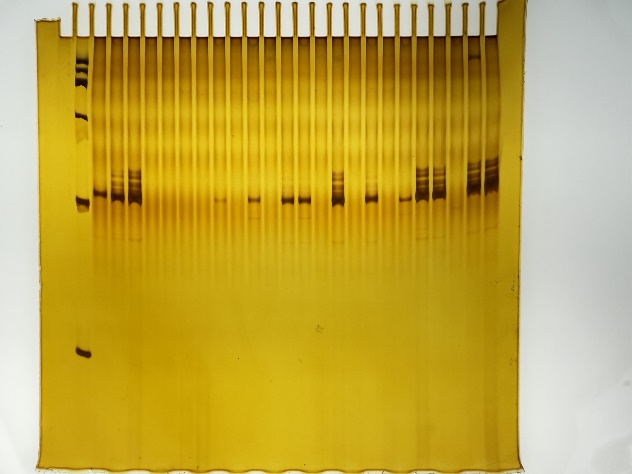70-93 |
| BLF-58 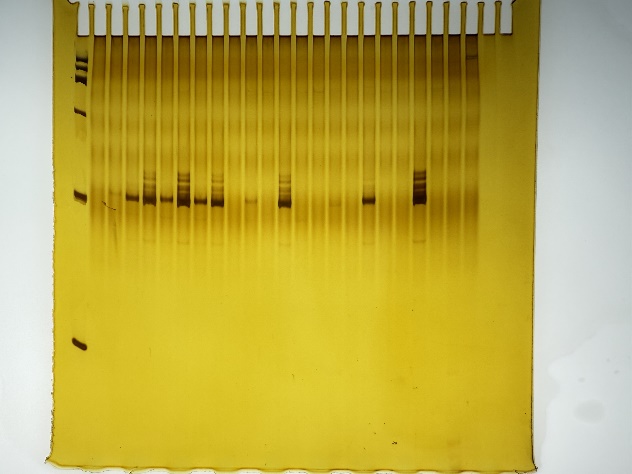94-116 | BLF-58 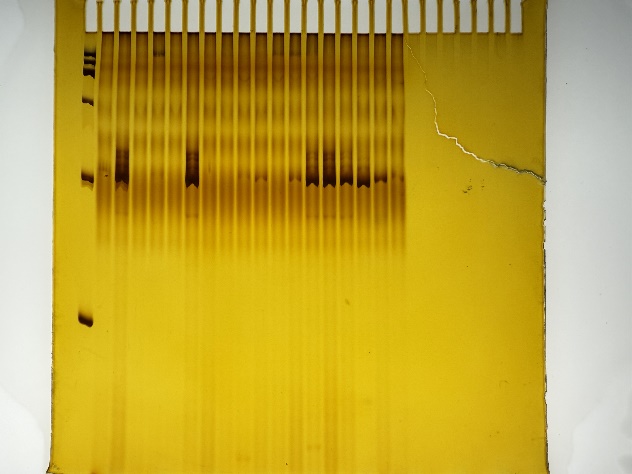117-134 |
| BLF-58 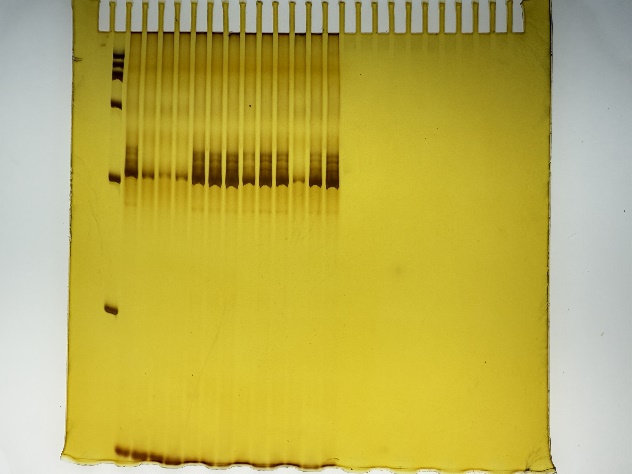135-147 |  |
| 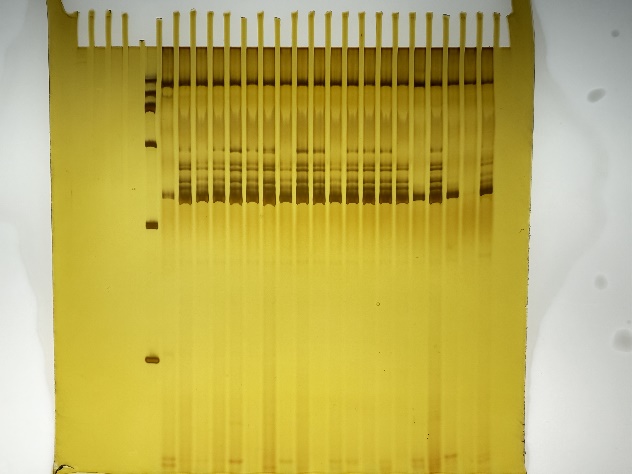BLF-59 1-20 | BLF-59 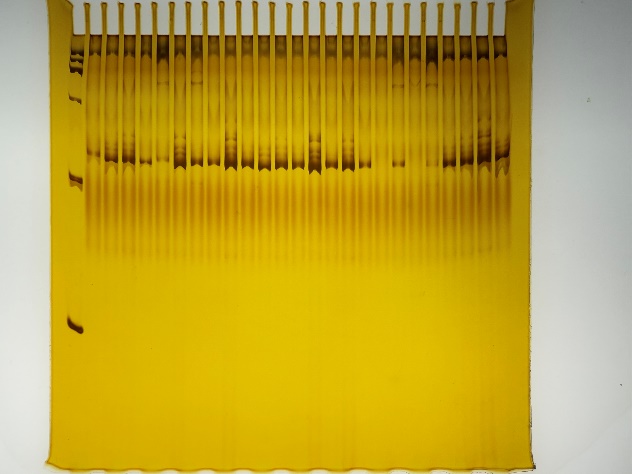21-45 |
| BLF-59 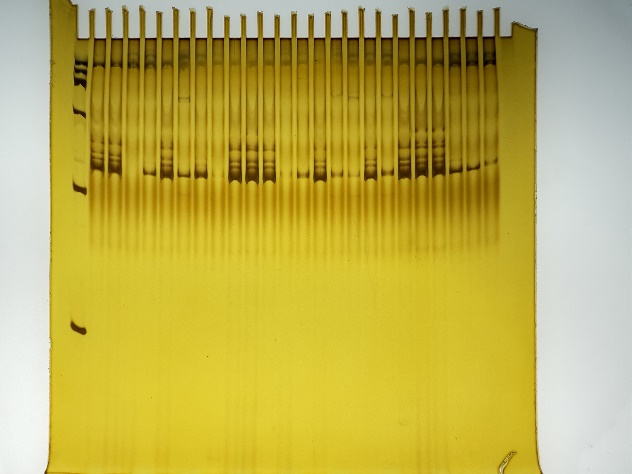46-69 | BLF-59 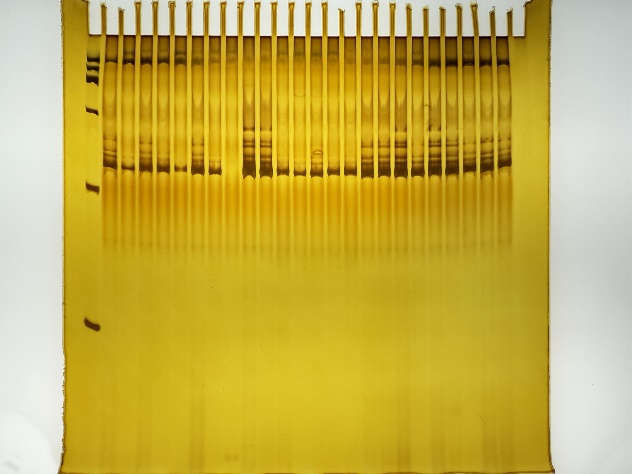70-93 |
| BLF-59 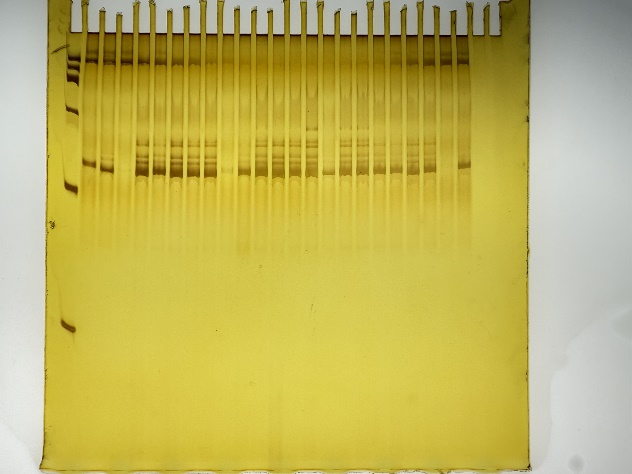94-116 | BLF-59 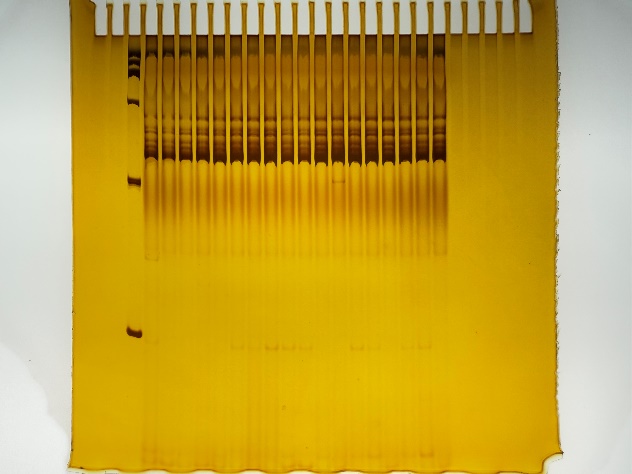117-134 |
| BLF-59 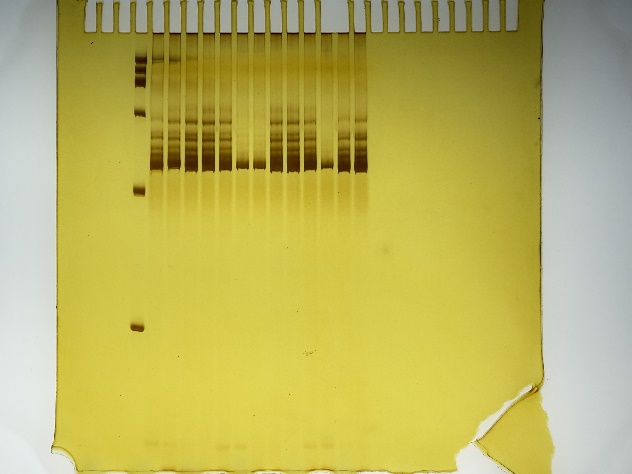135-147 |  |
| 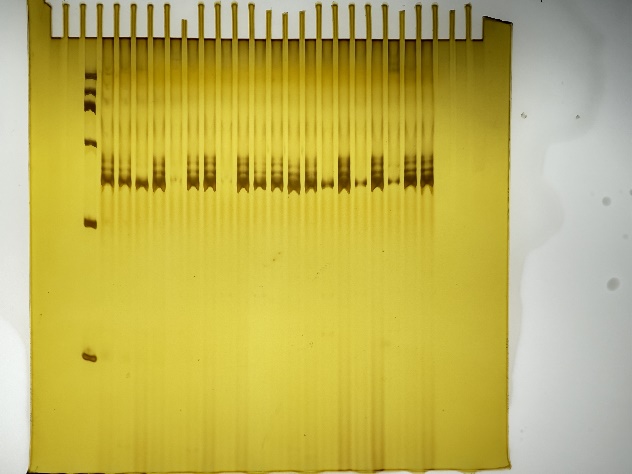BLF-61 1-20 | BLF-61 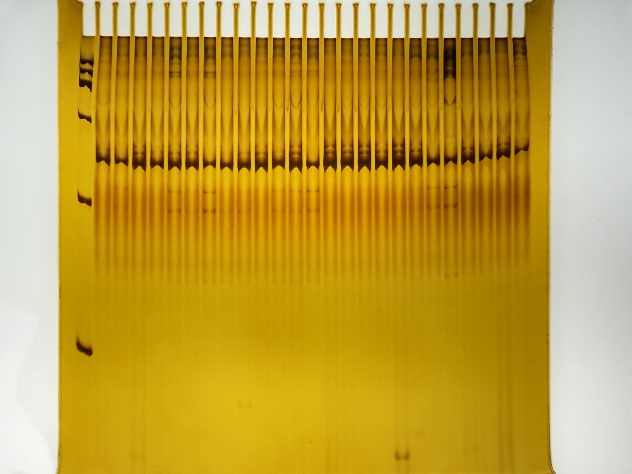21-45 |
| BLF-61 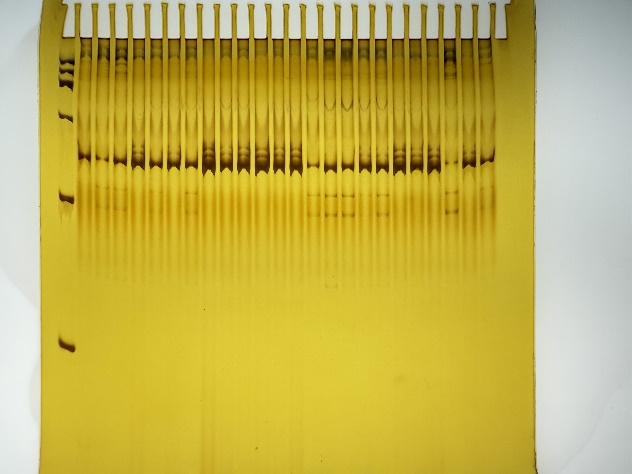46-69 | BLF-61 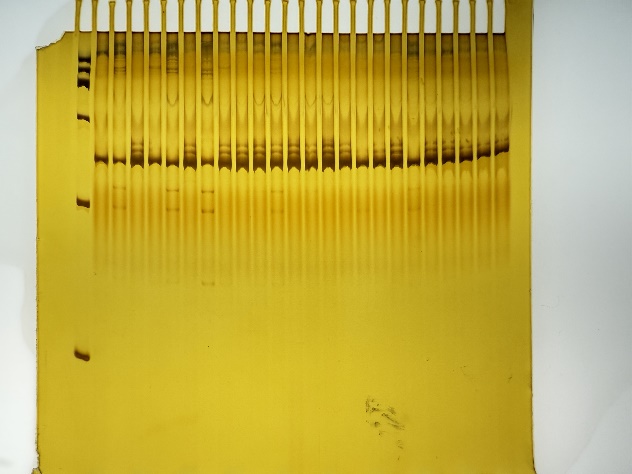70-93 |
| BLF-61 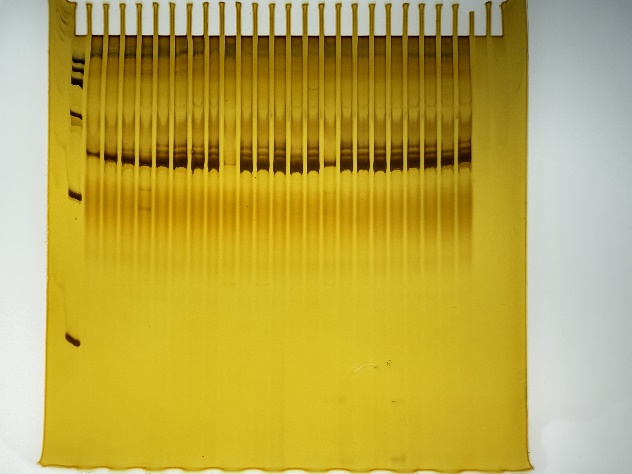94-116 | BLF-61 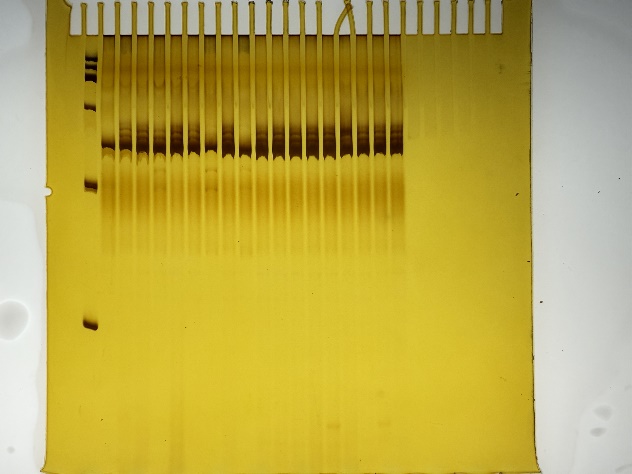117-134 |
| BLF-61 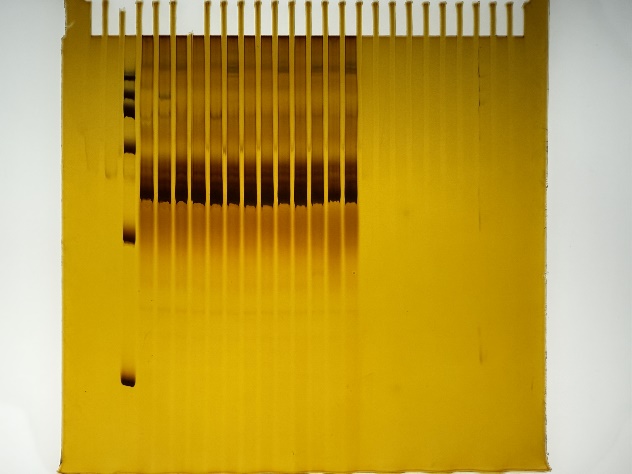135-147 |  |
| 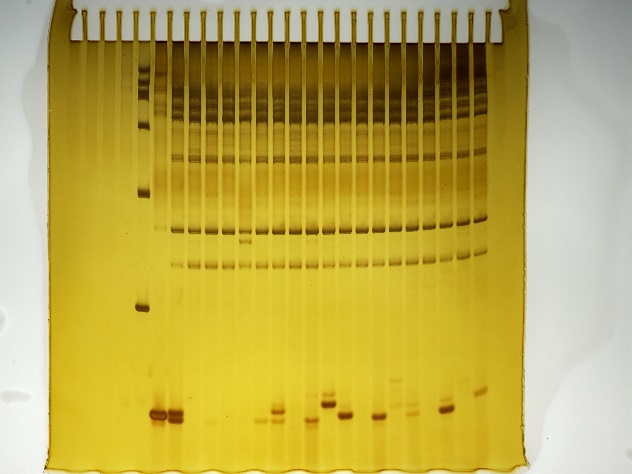BLF-21 1-20 | BLF-21 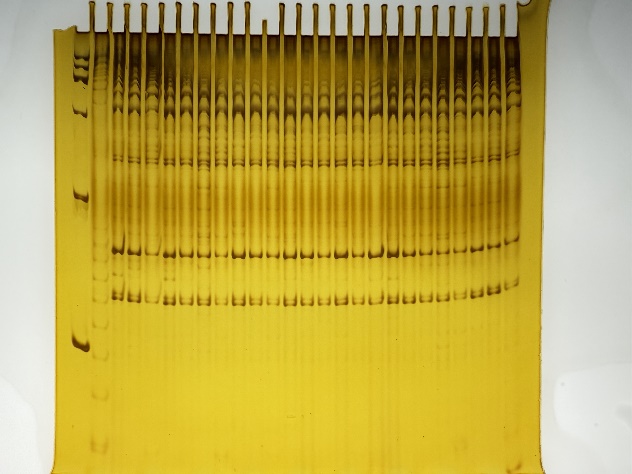21-45 |
| BLF-21 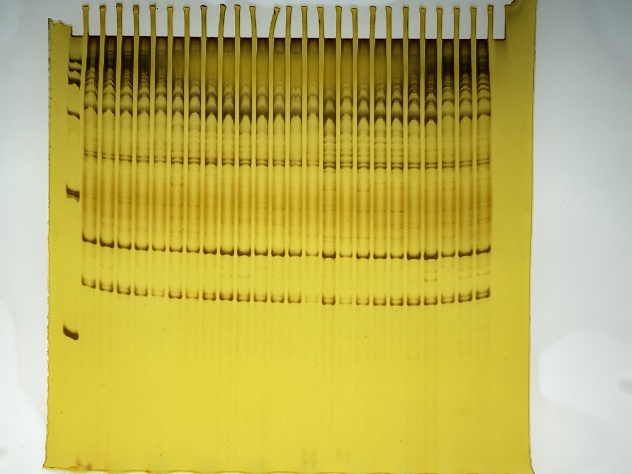46-69 | BLF-21 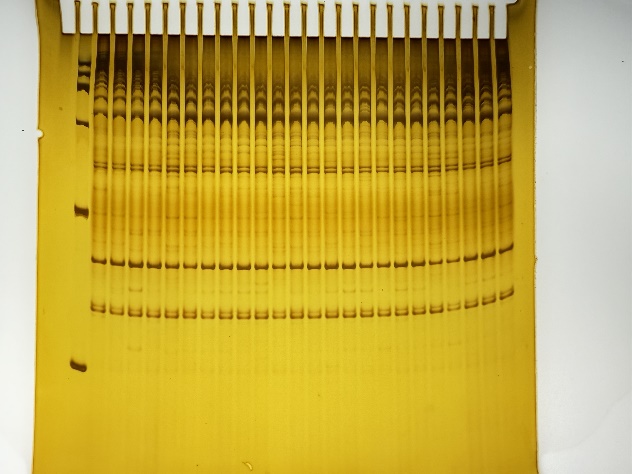70-93 |
| BLF-21 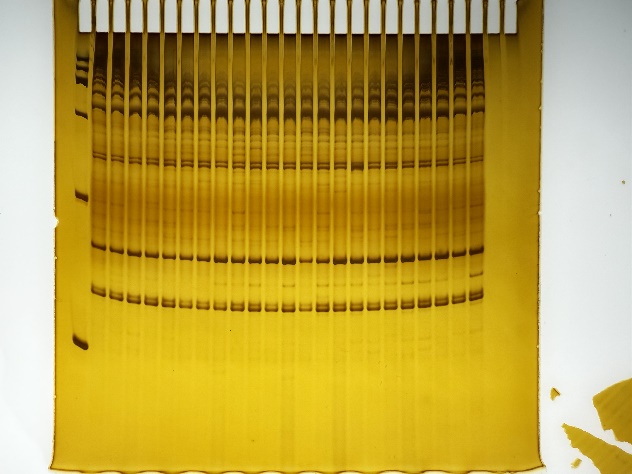94-116 | BLF-21 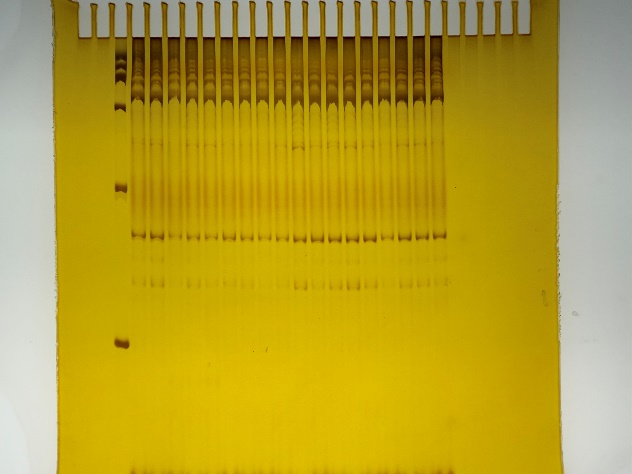117-134 |
| BLF-21 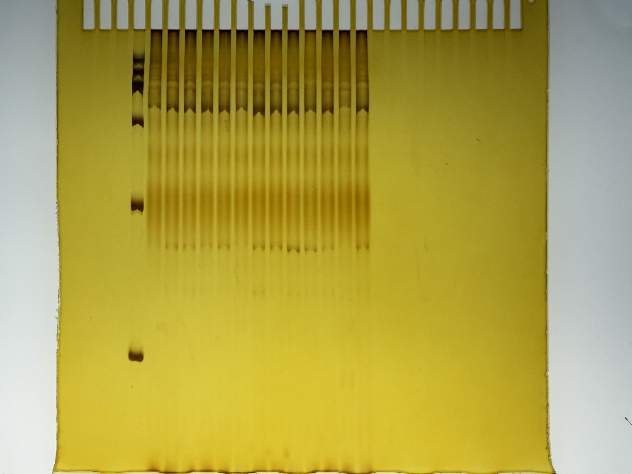135-147 |  |
| 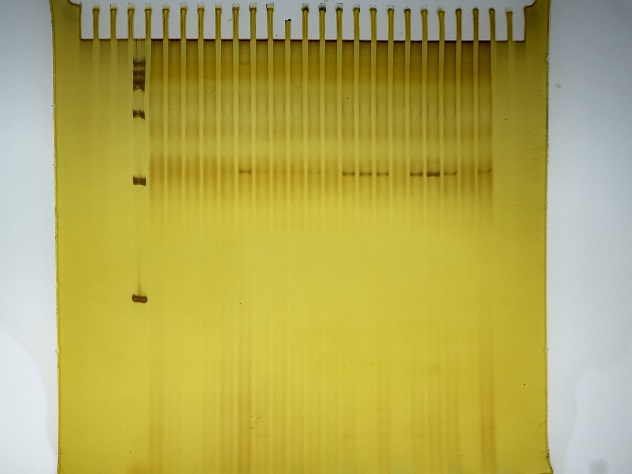BLF-66 1-20 | BLF-66 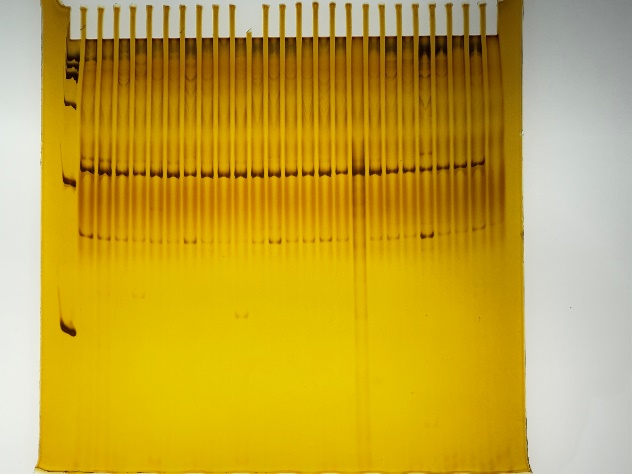21-45 |
| BLF-66 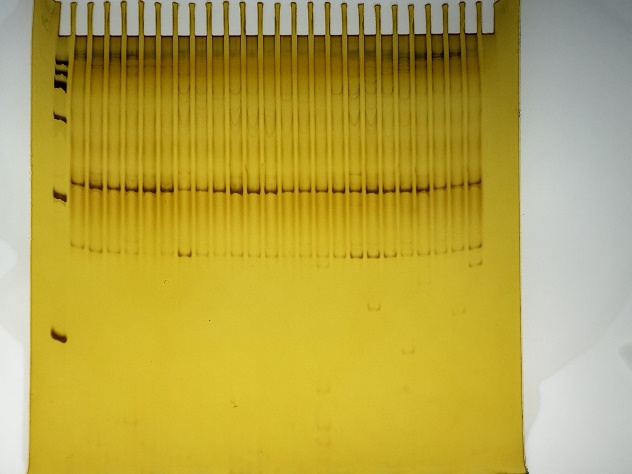46-69 | BLF-66 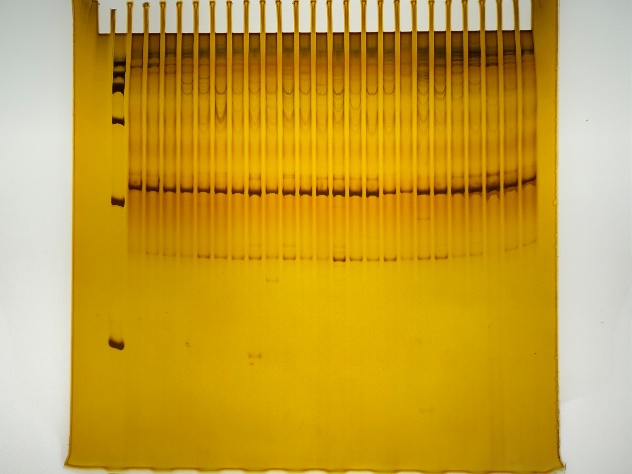70-93 |
| BLF-66 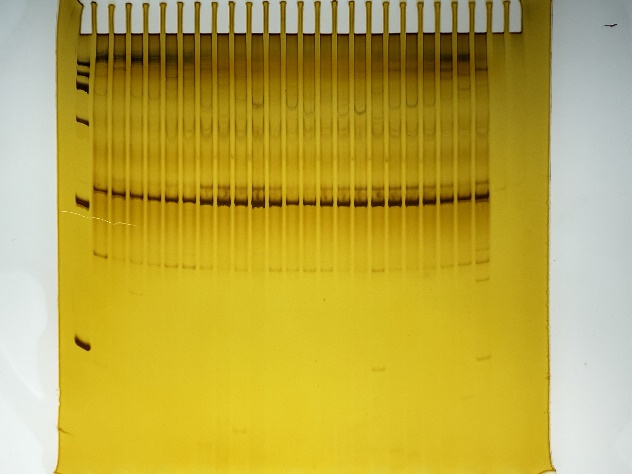94-116 | BLF-66 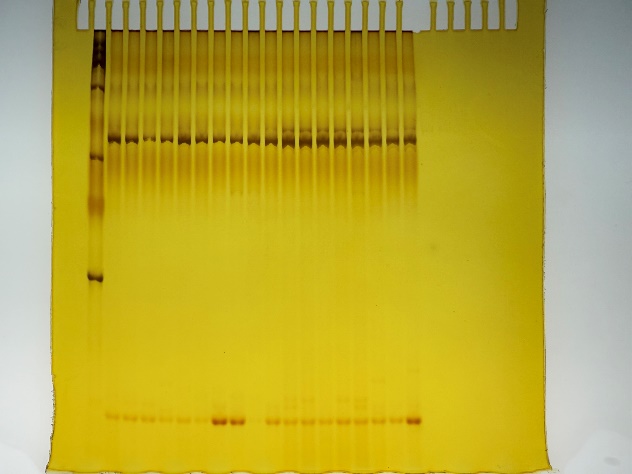117-134 |
| BLF-66 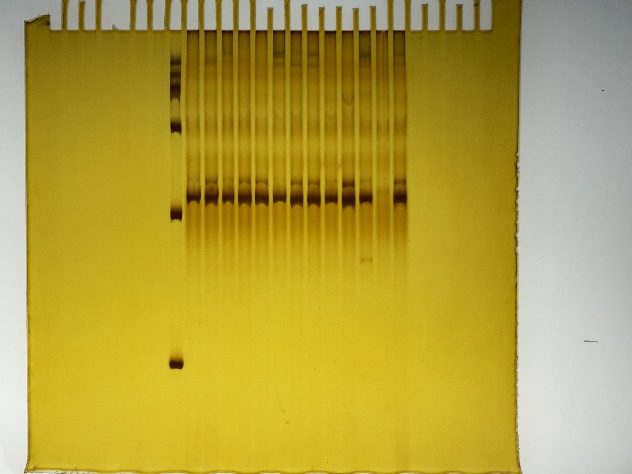135-147 |  |
| 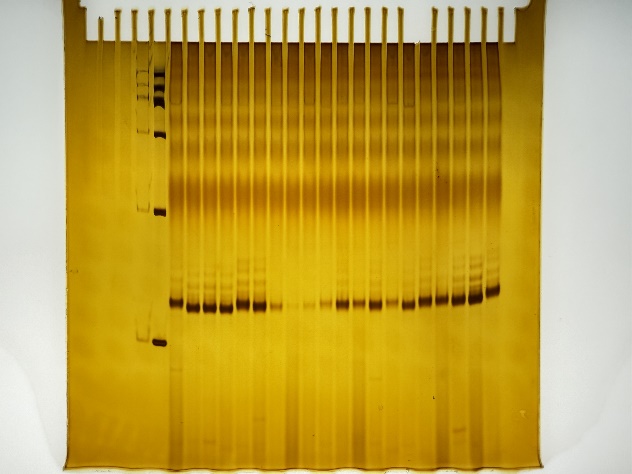BLF-27 1-20 | BLF-27 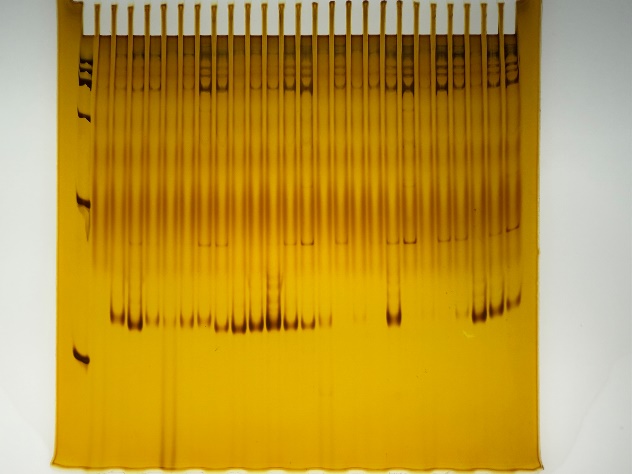21-45 |
| BLF-27 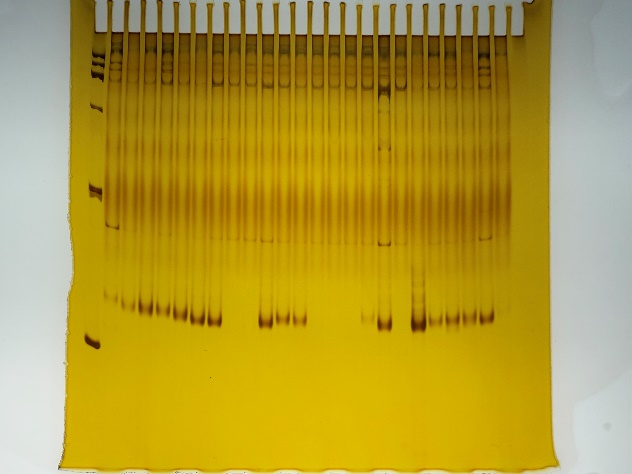46-69 | BLF-27 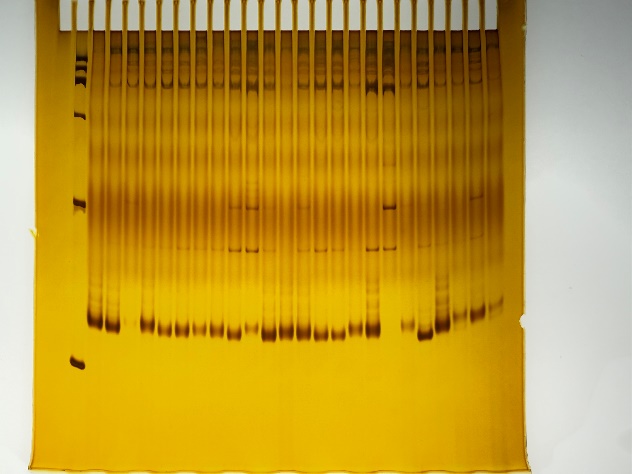70-93 |
| BLF-27 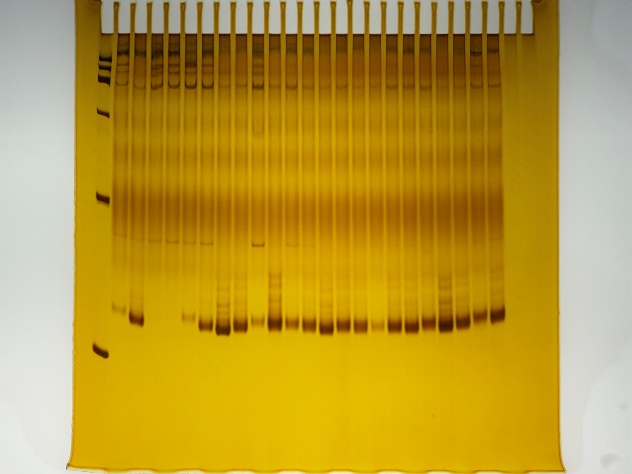94-116 | BLF-27 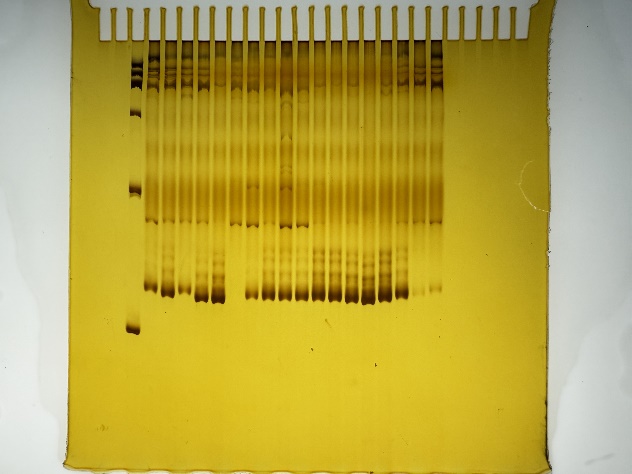117-134 |
| BLF-27 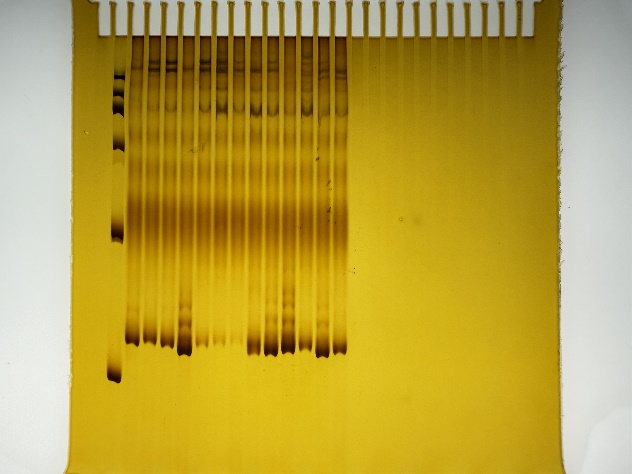135-147 |  |
| 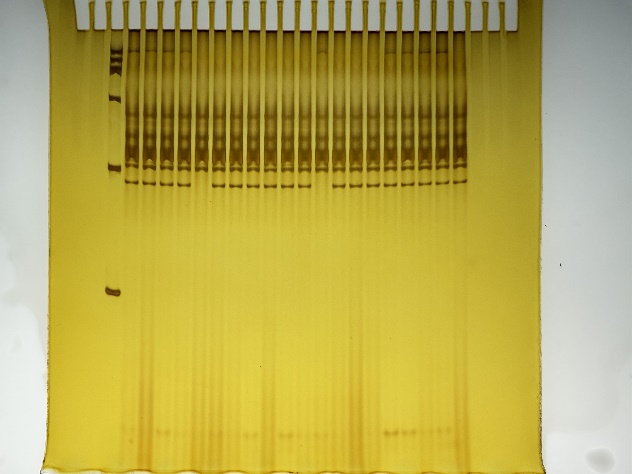BLF-79 1-20 | BLF-79 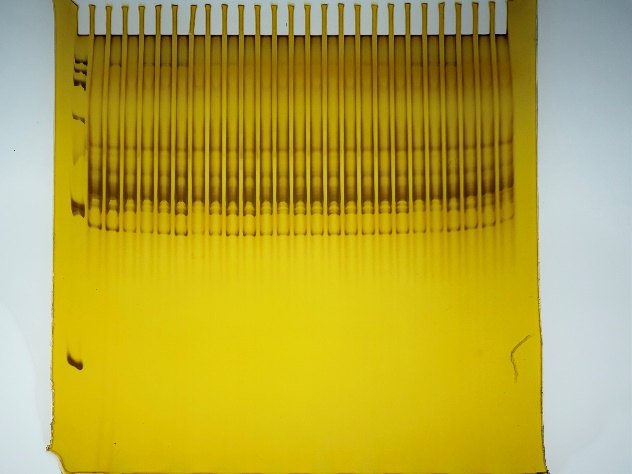21-45 |
| BLF-79 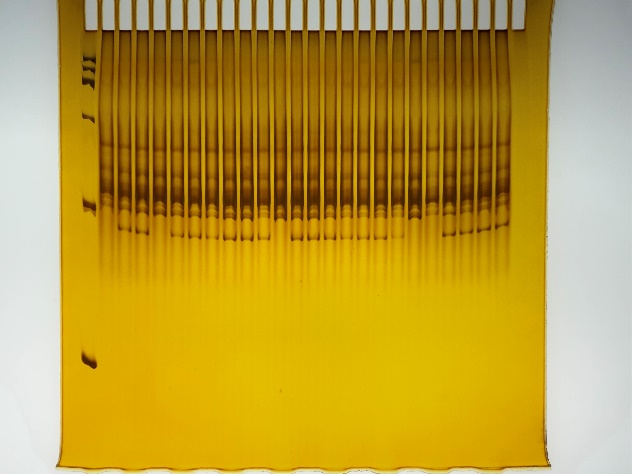46-69 | BLF-79 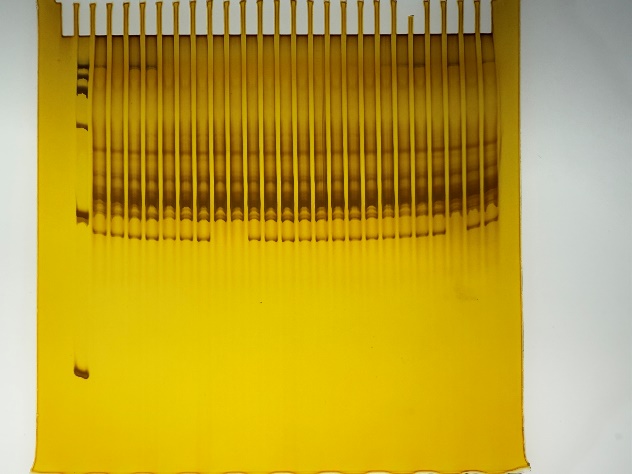70-93 |
| BLF-79 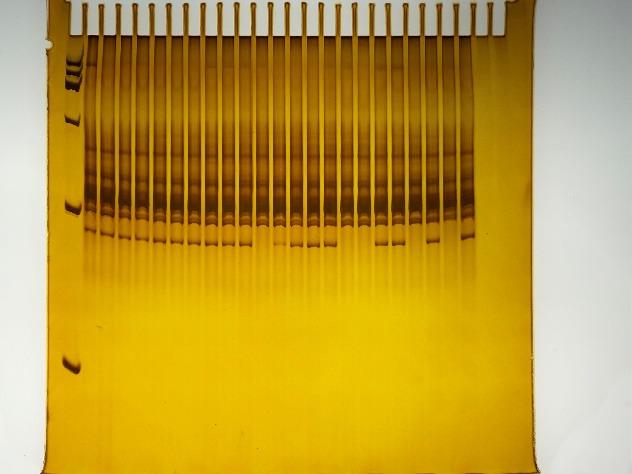94-116 | BLF-79 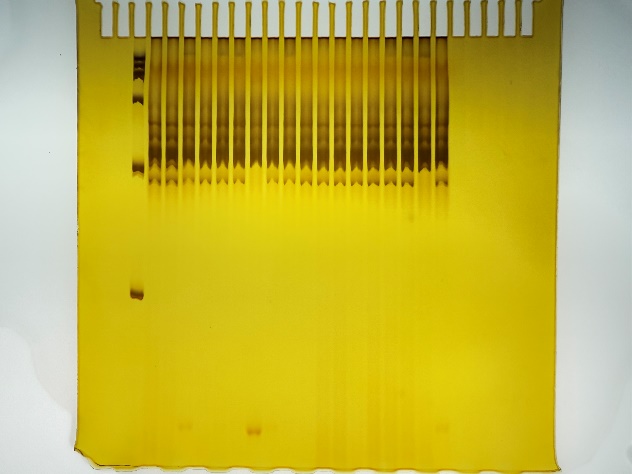117-134 |
| BLF-79 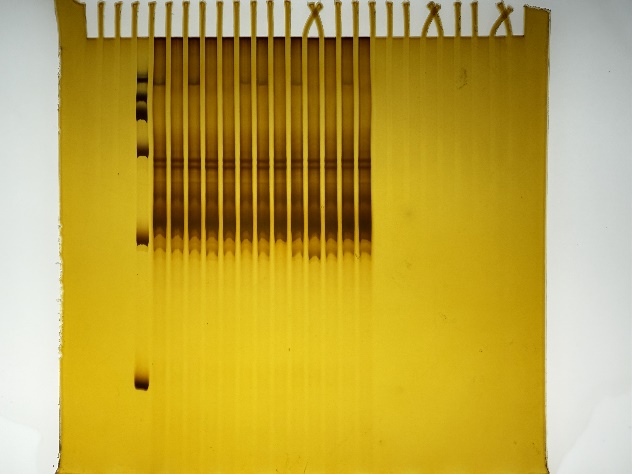135-147 |  |
| BLF-80 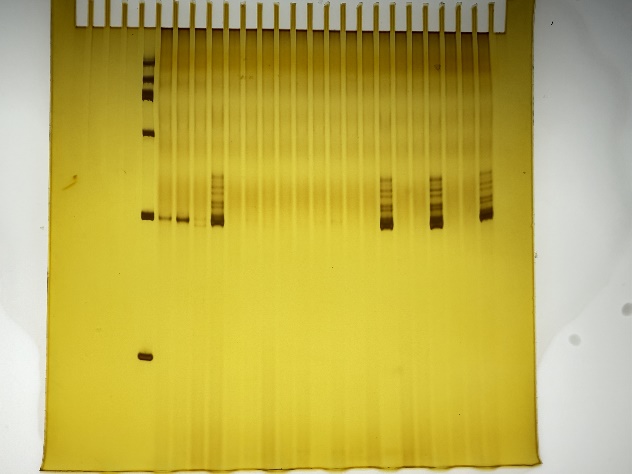1-20 | BLF-80 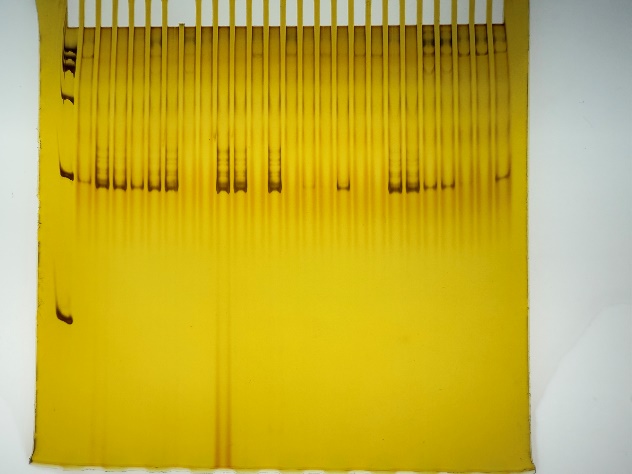21-45 |
| BLF-80 46-69 | BLF-80 70-93 |
| BLF-80 94-116 | BLF-80 117-134 |
| BLF-80 135-147 |  |
